# Cytokine production by activated plasmacytoid dendritic cells and natural killer cells is suppressed by an IRAK4 inhibitor

**DOI:** 10.1186/s13075-018-1702-0

**Published:** 2018-10-24

**Authors:** Karin Hjorton, Niklas Hagberg, Elisabeth Israelsson, Lisa Jinton, Olof Berggren, Johanna K. Sandling, Kristofer Thörn, John Mo, The DISSECT consortium, Maija-Leena Eloranta, Lars Rönnblom

**Affiliations:** 1 Department of Medical Sciences, Rheumatology, Science for Life Laboratory, Uppsala University, Rudbecklaboratoriet, Dag Hammarskjölds v 20, C11, 751 85 Uppsala, Sweden; 2 Respiratory, Inflammation and Autoimmunity, IMED Biotech Unit, AstraZeneca, Gothenburg, Sweden

**Keywords:** SLE, pDC, NK, HCQ, IRAK4

## Abstract

**Background:**

In systemic lupus erythematosus (SLE), immune complexes (ICs) containing self-derived nucleic acids trigger the synthesis of proinflammatory cytokines by immune cells. We asked how an interleukin (IL)-1 receptor-associated kinase 4 small molecule inhibitor (IRAK4i) affects RNA-IC-induced cytokine production compared with hydroxychloroquine (HCQ).

**Methods:**

Plasmacytoid dendritic cells (pDCs) and natural killer (NK) cells were isolated from peripheral blood mononuclear cells (PBMCs) of healthy individuals. PBMCs from SLE patients and healthy individuals were depleted of monocytes. Cells were stimulated with RNA-containing IC (RNA-IC) in the presence or absence of IRAK4i I92 or HCQ, and cytokines were measured by immunoassay or flow cytometry. Transcriptome sequencing was performed on RNA-IC-stimulated pDCs from healthy individuals to assess the effect of IRAK4i and HCQ.

**Results:**

In healthy individuals, RNA-IC induced interferon (IFN)-α, tumor necrosis factor (TNF)-α, IL-6, IL-8, IFN-γ, macrophage inflammatory protein (MIP)1-α, and MIP1-β production in pDC and NK cell cocultures. IFN-α production was selective for pDCs, whereas both pDCs and NK cells produced TNF-α. IRAK4i reduced the pDC and NK cell-derived cytokine production by 74–95%. HCQ interfered with cytokine production in pDCs but not in NK cells. In monocyte-depleted PBMCs, IRAK4i blocked cytokine production more efficiently than HCQ. Following RNA-IC activation of pDCs, 975 differentially expressed genes were observed (false discovery rate (FDR) < 0.05), with many connected to cytokine pathways, cell regulation, and apoptosis. IRAK4i altered the expression of a larger number of RNA-IC-induced genes than did HCQ (492 versus 65 genes).

**Conclusions:**

The IRAK4i I92 exhibits a broader inhibitory effect than HCQ on proinflammatory pathways triggered by RNA-IC, suggesting IRAK4 inhibition as a therapeutic option in SLE.

**Electronic supplementary material:**

The online version of this article (10.1186/s13075-018-1702-0) contains supplementary material, which is available to authorized users.

## Background

Systemic lupus erythematosus (SLE) is characterized by circulating immune complexes (ICs), an activation of the type I interferon (IFN) system, and production of proinflammatory cytokines and chemokines which cause an autoimmune reaction with organ inflammation [1]. The cellular and molecular mechanisms behind the ongoing inflammatory process in SLE have been partially clarified, and a number of different disease-associated pathways identified [2]. One important event is the induction of type I IFN production by plasmacytoid dendritic cells (pDCs) in response to ICs consisting of autoantibodies and apoptotic or necrotic cell-derived nucleic acids [3]. Such interferogenic ICs are internalized in pDCs via fragment crystallizable receptor IIA (FcγRIIA) and directed to the endosomes, where RNA and DNA interact with Toll-like receptor (TLR)7 and 9, respectively [4]. Activation of TLR7/9 triggers a signaling cascade, involving myeloid differentiation primary response protein 88 (MyD88), interleukin (IL)-1 receptor-associated kinase (IRAK)1, and IRAK4, that eventually leads to transcription of type I IFN genes. In addition to type I IFN production, MyD88 and IRAK4 signaling triggers the production of other proinflammatory cytokines, such as tumor necrosis factor (TNF)-α and IL-6, via activation of nuclear factor kappa-light-chain-enhancer of activated B cells (NFκB) or IFN regulatory factor (IRF) 5 [5]. Besides the pDCs, interferogenic ICs will also activate several other immune cells, such as natural killer (NK) cells, which contribute to enhanced cytokine production [6]. The final outcome in SLE is a complex inflammatory response that is difficult to bring into complete remission.

Current therapies in SLE aim to downregulate the autoimmune reaction. Treatment with antimalarials, such as hydroxychloroquine (HCQ), is considered the standard of care [7, 8]. The presumed central mechanism of action of HCQ is a reduction in the IFN-α production by inhibition of endosomal TLR signaling [9]. Studies have also shown that SLE patients treated with HCQ have a decreased type I IFN production after stimulation of pDCs with TLR ligands [10]. However, despite continuous HCQ treatment, few patients with SLE experience complete remission and flares still occur. A possible reason could be the limited number of disease-associated pathways affected by HCQ. Consequently, targeting a broader repertoire of inflammatory cytokines in SLE, yet avoiding severe infections, is needed. A potential therapeutic target in SLE is IRAK4 due to its essential role in MyD88 signaling [11]. IRAK4-deficient children are susceptible to life-threatening pyogenic infections that are reported to cease in adolescence, making IRAK4 inhibition an attractive therapeutic possibility [12].

In this study, we compared the effect of HCQ and the IRAK4 inhibitor (IRAK4i) I92 on the RNA-IC-induced cytokine production by pDCs and NK cells from healthy individuals and monocyte-depleted peripheral blood mononuclear cells (PBMCs) from SLE patients and healthy controls. Gene expression profiles of RNA-IC-stimulated pDCs treated with IRAK4i or HCQ were compared with nontreated cells to clarify the inflammatory response modulated by the drugs.

## Methods

### Patients and controls

All SLE patients (*n* = 15) fulfilled ≥ 4 of the American College of Rheumatology criteria for SLE [13]. Patients were a median 52 (range 32–81) years old with a disease duration of 15 (1–46) years (Additional file 1). Healthy age- and gender-matched controls (*n* = 12) were 53 (32–68) years old. The local ethics committee at Uppsala University approved the study and informed consent was obtained from all patients and controls.

### Cell isolation and culture conditions

PBMCs were prepared from healthy donor buffy coats by Ficoll density-gradient centrifugation. pDCs (25 × 10^3^/well) and NK cells (50 × 10^3^/well) were isolated and cultivated as previously described [6, 14]. Cell purity was > 95% for pDC (blood dendritic cell antigen (BDCA)2^+^) and NK cells (cluster of differentiation (CD)56^+^) as determined by flow cytometry. Cell viability as measured by flow cytometry after 20 h was approximately 90%.

### Interferon inducers

Necrotic material from U937 cell line and U1snRNP particles were prepared as previously described [3, 15]. Immunoglobulin (Ig)G was isolated from two Smith nuclear antigen (Sm) and ribonucleoprotein (RNP) antibody-containing SLE patient sera [14]. U1snRNP particles and SLE IgG were used at final concentrations of 2.5 μg/ml and 1 mg/ml, respectively.

### Drugs

The small molecule drug IRAK4i I92 (ND-2158, Nimbus Discovery) [16] and HCQ (Sigma-Aldrich) were pretitrated and used at final concentrations of 10 μM and 5.8 μM (pDCs and NK cells) or 7.8 μM (monocyte-depleted PBMCs) (Additional files 2, 3 and 4). The cells were preincubated with I92 or HCQ for 30 min at 5% CO_2_ and 37 °C before adding IFN inducers.

### Flow cytometry

pDCs and NK cells were cultivated for 5 or 9 h, with the final 4 h with brefeldin A. After gating live cells, singlets, and lymphocytes (Additional file 5), the cells were identified with anti-CD56-phycoerythrin (PE)Cy7 (NCAM 16.2, BD Biosciences) and BDCA-2-fluorescein isothiocyanate (FITC) (AC144, Miltenyi Biotech) monoclonal antibodies (mAbs). Intracellular cytokines were detected with anti-TNF-α-allophycocyanin (APC) (Mab11, BD Pharmingen) and anti-IFN-α-PE mAbs (LT27:295, Miltenyi Biotech). Isotype-matched irrelevant mAbs were used as controls. Live/dead Fixable Near-InfraRed Dead Cell Stain (Invitrogen) was used to distinguish live cells. Data were acquired with a FACS CantoII instrument and analyzed with Diva 6.1.3 software (BD Biosciences).

### Immunoassays

TNF-α, IL-6, IL-8, IFN-γ, macrophage inflammatory protein (MIP)1-α, and MIP1-β were measured after 20 h by multiplex immunoassays (Milliplex Human Cytokine/Chemokine (Millipore) or Luminex Screening Assay (R&D systems)). Lower limits of quantification (LLoQ) of cytokines were: TNF-α, 3.8 pg/ml; IL-6, 5.2 pg/ml; IL-8, 1.4 pg/ml; IFN-γ, 1.6 pg/ml; MIP1-α, 54.8; and MIP1-β, 162.2 pg/ml. IFN-α was measured by dissociation-enhanced lanthanide fluorescence immunoassay (DELFIA; LLoQ, 2 U/ml) [17].

### RNA sequencing

pDCs from four healthy individuals were stimulated with RNA-IC for 6 h. RNA (RNA integrity number (RIN) ≥ 8) was extracted by the RNeasy 96 plus kit (Qiagen). RNA libraries were prepared with the TruSeq Stranded mRNA kit and sequenced by NextSeq500 (Illumina). RNAseq fastq files were processed using bcbio-nextgen (v.0.9.7) and mapped to the human genome GRCh38.79 [18]. Gene-level quantifications were generated with featureCounts software (v.1.4.4) [19] and Sailfish (version 0.9.0) [20]. Pheatmap and ggplot2 (v.2.2.1, http://ggplot2.org/) were used for visualizations [21].

### Statistical analysis

Statistical analysis was performed using GraphPad Prism software 7.0. Differences were analyzed with Friedman’s test, and *p* values ≤ 0.05 were considered significant. For transcriptome analysis, a false discovery rate (FDR) < 0.05 was considered significant. Analyses were performed using R (version 3.3.3). Differential gene expression was assessed with DESeq2 (v.1.14.1) [22] using raw counts as input. Pathway enrichments were obtained from Pathway Studio® (Elsevier). A one-sided Mann-Whitney *U* test was performed to calculate the significance of the differences in distribution between the background (from the differential gene expression analysis) and the gene subnetworks (upstream regulators) or the gene sets (pathways).

## Results

### RNA-containing ICs induce TNF-α production more rapidly in NK cells than in pDCs

**Fig. 1:**
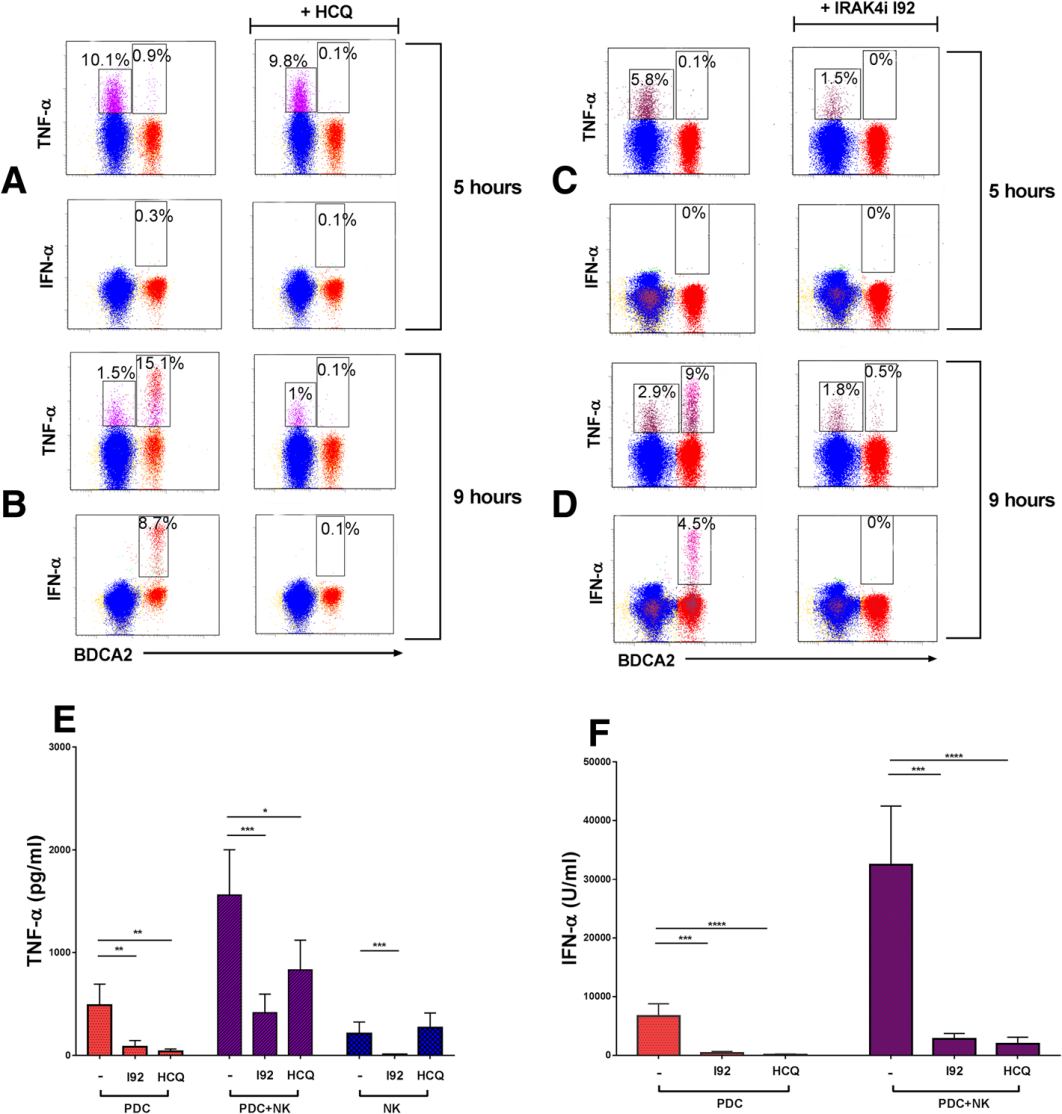
Regulatory effect of hydroxychloroquine (HCQ) and the interleukin-1 receptor associated kinase 4 inhibitor (IRAK4i) I92 on tumor necrosis factor (TNF)-α and interferon (IFN)-α production. **a**–**d** Cocultures of plasmacytoid dendritic cells (pDC) and natural killer (NK) cells from healthy donors were stimulated with RNA-containing immune complexes (RNA-IC) for 5 h (**a**,**b**) or 9 h (**c**,**d**) in the absence or presence of HCQ or I92. The frequencies of TNF-α- and IFN-α-producing NK cells (blue) and pDCs (red) were determined by flow cytometry. The dot plots represent one representative individual donor from two (HCQ) and four (IRAK4i) donors analyzed. **e**,**f** pDCs or NK cells were cultivated separately or in coculture in the presence of RNA-IC with or without I92 or HCQ. Cytokine levels were measured by immunoassays after 20 h. No IFN-α is produced by NK cells (data not shown). No cytokines were detected in the cell cultures in the absence of RNA-IC. Bars represent the mean with standard error of the mean (SEM) of nine donors from at least three independent experiments. Friedman’s test, uncorrected Dunn’s test; *p < 0.05, **p < 0.01, ***p < 0.001, ****p < 0.0001. BDCA blood dendritic cell antigen

TNF-α and IFN-α are important drivers of inflammation in SLE and large amounts are produced in RNA-IC-stimulated cocultures of pDCs and NK cells [6]. However, the cellular source and quantity produced by each cell type have not been determined. Therefore, we initially analyzed the frequency of TNF-α- and IFN-α-producing pDCs and NK cells in cocultures at 5 and 9 h, due to expected differences regarding peak cytokine production by the different cell types. A minority (< 20%) of both pDCs and NK cells produced TNF-α in response to RNA-IC (Fig. 1a, b; left panels). Furthermore, the TNF-α production was prominent in NK cells at 5 h, but occurred later in pDCs when the NK cell response had decreased.

Only pDCs produced IFN-α in response to RNA-IC (Fig. 1a, b; left panels), and synthesis of both TNF-α and IFN-α was most prominent at 9 h (Fig. 1b). Nearly all IFN-α-producing cells expressed TNF-α, whereas a fraction of the pDCs produced TNF-α only (Fig. 1b, left panel; Additional file 6). Almost no IFN-α-containing pDCs were detected at 5 h.

Therefore, RNA-containing ICs trigger the production of TNF-α in a fraction of pDCs and NK cells at different time points.

### IRAK4i I92 inhibits TNF-α production by activated pDCs and NK cells, while HCQ affects TNF-α production by pDCs only

Next, we asked if HCQ inhibits the RNA-IC-induced TNF-α production in pDC/NK cell cocultures. As shown in Fig. 1a, b (right panels), HCQ completely blocked TNF-α production in pDCs but not in NK cells at 9 h. At 20 h, HCQ significantly reduced TNF-α production in cultures of pDCs and pDC/NK cells, but not in NK cells (Fig. 1e). In contrast, IFN-α production was completely blocked by HCQ. Subsequently, we investigated if IRAK4i I92 could inhibit the cytokine response in the cell cultures. As shown in Fig. 1c, d (right panels), intracellular IFN-α and TNF-α production by pDCs was effectively blocked by I92. The drug reduced the early (5-h) TNF-α response by approximately 75% in NK cells, whereas the inhibitory effect at a later time point (9 h) was less prominent. At 20 h, I92 reduced the TNF-α production by 70–95% in all cell cultures (Fig. 1e). In addition, the IFN-α levels in RNA-IC-stimulated pDC and pDC-NK cell cocultures were reduced by > 90% (Fig. 1f). No IFN-α was produced by NK cells. Thus, HCQ blocked TNF-α and IFN-α production by pDCs, whereas I92 reduced TNF-α production in both pDCs and NK cells, as well as the IFN-α production by pDCs.

### IRAK4 inhibition reduces proinflammatory cytokine production by activated pDCs and NK cells more extensively than HCQ

**Fig. 2:**
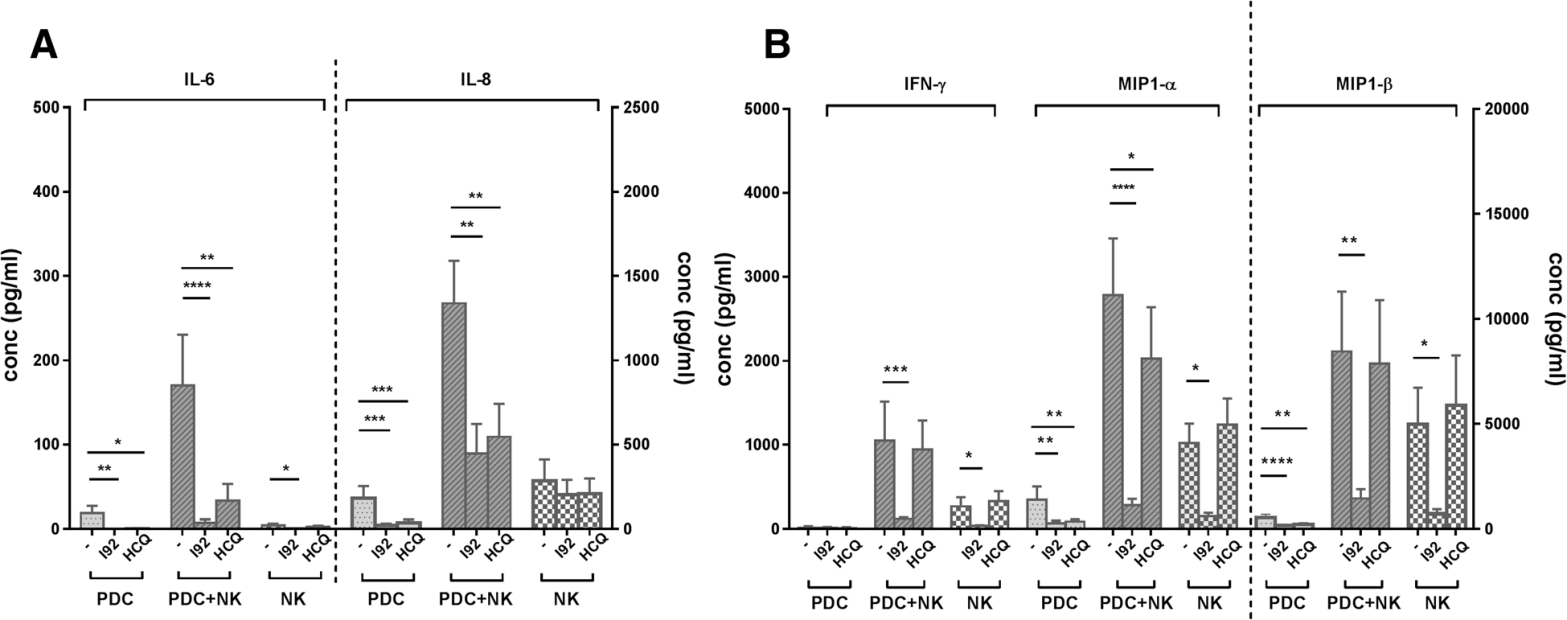
The interleukin-1 receptor associated kinase 4 inhibitor (IRAK4i) I92 displays a more prominent inhibitory effect than hydroxychloroquine (HCQ) on the production of proinflammatory cytokines in plasmacytoid dendritic cells (pDC) and natural killer (NK) cell cocultures and in NK cells alone. pDCs and NK cells from healthy donors were cultivated separately or in coculture in the presence of RNA-containing immune complexes (RNA-IC), with or without I92 or HCQ. The levels of **a** interleukin (IL)-6 and IL-8, and **b** interferon (IFN)-γ, macrophage inflammatory protein (MIP)1-α, and MIP1-β were measured by immunoassays after 20 h. Bars represent the mean with standard error of the mean (SEM) of nine donors from at least three independent experiments. Friedman’s test, uncorrected Dunn’s test; *p < 0.05, **p < 0.01, ***p < 0.001, ****p < 0.0001

To investigate whether IRAK4 inhibition affects production of other proinflammatory cytokines, levels of IL-6, IL-8, IFN-γ, MIP1-α, and MIP1-β were measured in RNA-IC-stimulated cultures with pDCs, NK cells, or pDC/NK cell cocultures in the presence or absence of I92 or HCQ (Fig. 2). In pDC cultures and pDC/NK cocultures, both I92 and HCQ blocked IL-6, IL-8, and MIP1-α, whereas I92 also blocked IFN-γ and MIP1-β production. In NK cell cultures, I92 significantly reduced IL-6, IFN-γ, MIP1-α, and MIP1-β levels, whereas IL-8 was unaffected. HCQ did not inhibit cytokine production by NK cells. Hence, I92 showed a broader inhibitory effect than HCQ on proinflammatory cytokines produced by RNA-IC-stimulated pDCs and NK cells.

### IRAK4 inhibition blocks production of proinflammatory cytokines by PBMCs from SLE patients

**Fig. 3:**
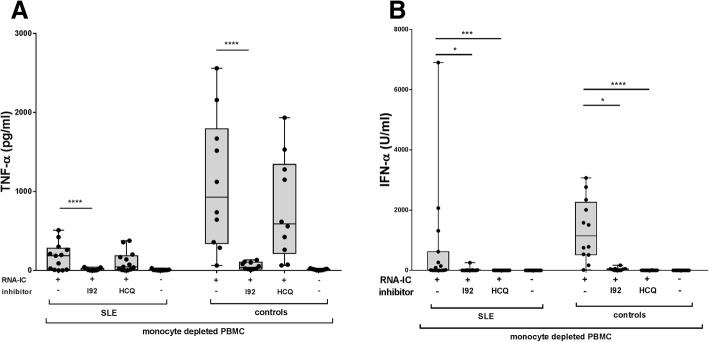
The interleukin-1 receptor associated kinase 4 inhibitor (IRAK4i) I92 reduces both **a** tumor necrosis factor (TNF)-α and **b** interferon (IFN)-α production by monocyte-depleted peripheral blood mononuclear cells (PBMCs) from systemic lupus erythematosus (SLE) patients and healthy controls. The cells were stimulated with RNA-containing immune complexes (RNA-IC) in the presence or absence of I92 or hydroxychloroquine (HCQ), or were mock stimulated. TNF-α and IFN-α production in cell cultures was measured after 20 h by immunoassays. Box plots show median with interquartile range, based on 10–15 donors, in at least 10 independent experiments. Friedman’s test, uncorrected Dunn’s test; *p < 0.05, **p < 0.01, ***p < 0.001, ****p < 0.0001

**Fig. 4:**
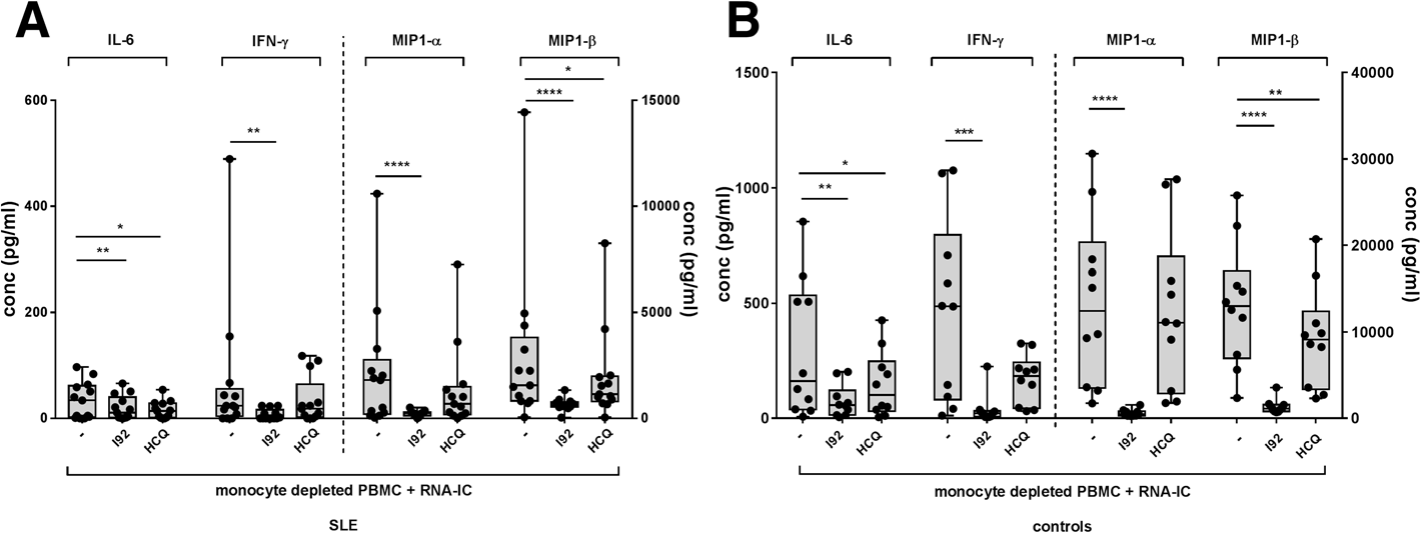
The interleukin-1 receptor associated kinase 4 inhibitor (IRAK4i) I92 exhibits a broader inhibitory effect than hydroxychloroquine (HCQ) on cytokine production by monocyte-depleted peripheral blood mononuclear cells (PBMCs) from **a** systemic lupus erythematosus (SLE) patients and **b** healthy controls. Monocyte-depleted PBMCs were stimulated with RNA-containing immune complexes (RNA-IC) in the presence or absence of I92 or HCQ. Levels of interleukin (IL)-6, interferon (IFN)-γ, macrophage inflammatory protein (MIP)1-α, and MIP1-β in the cell cultures were measured after 20 h by immunoassays. Box plots show median with interquartile range, based on 10–13 donors from 10 independent experiments. Friedman’s test, uncorrected Dunn’s test; *p < 0.05, **p < 0.01, ***p < 0.001, ****p < 0.0001

As healthy individuals and SLE patients may respond differently, we investigated if cytokine production in cells from patients with SLE also could be targeted with an IRAK4i. PBMCs from patients with SLE or healthy controls were stimulated after depletion of monocytes, due to their suppressive effect on the IFN-α response [14]. I92 inhibited TNF-α and IFN-α production by 80% and 97%, respectively, whereas HCQ interfered significantly only with IFN-α production (99%) (Fig. 3a, b). Furthermore, IL-6, IFN-γ, MIP1-α, and MIP1-β production was inhibited by I92, whereas only IL-6 and MIP1-β were significantly inhibited by HCQ (Fig. 4a, b). HCQ and I92 displayed the same inhibitory profile in monocyte-depleted PBMCs from healthy individuals. In summary, I92 reduced cytokine production in SLE patients and demonstrated a more extensive inhibitory profile on proinflammatory cytokines than did HCQ.

### Gene expression changes in RNA-IC-stimulated pDCs are reversed by both IRAK4 inhibition and HCQ

**Fig. 5:**
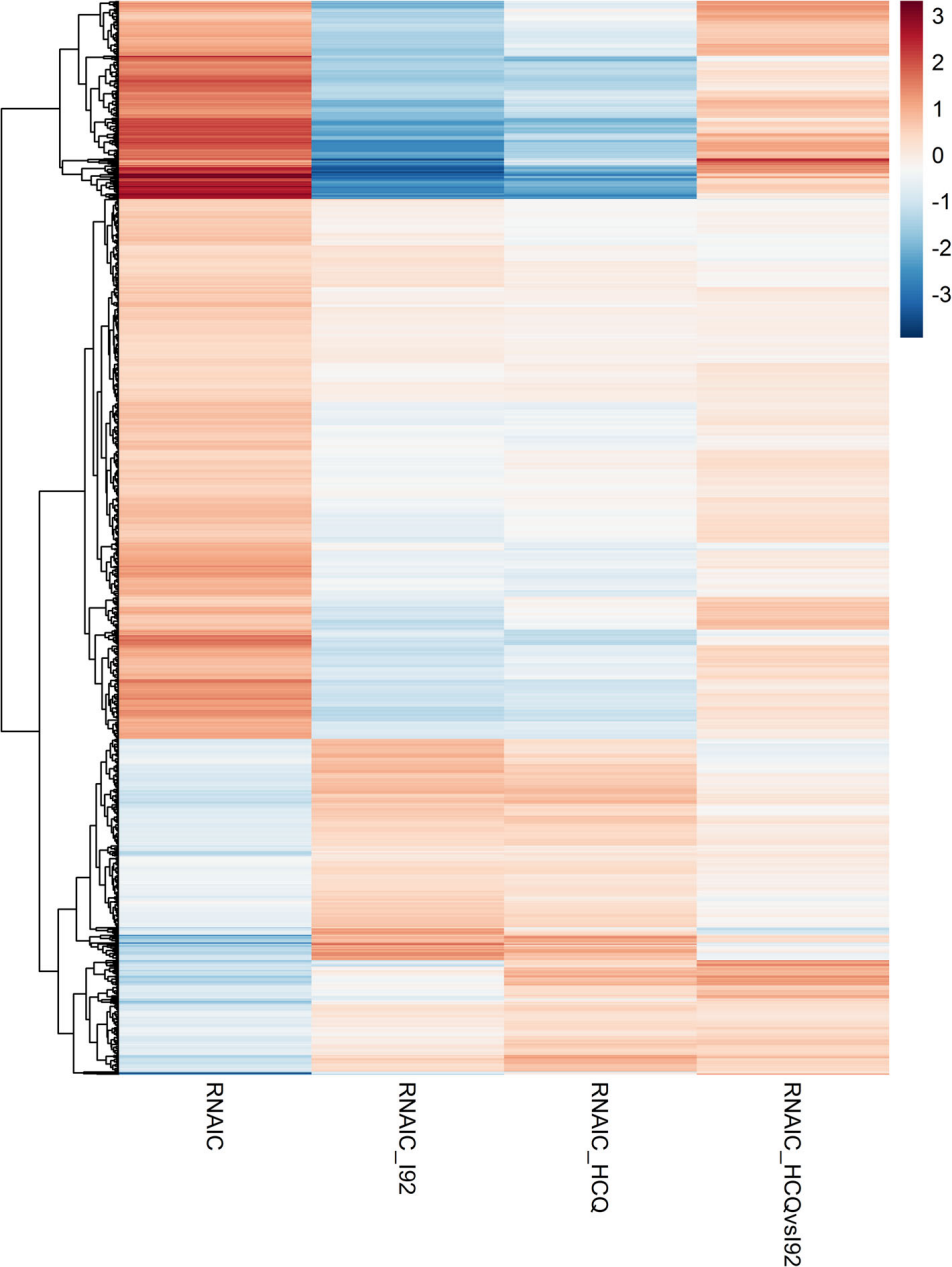
Differential effects on gene expression in RNA-containing immune complex (RNA-IC)-stimulated plasmacytoid dendritic cells (pDCs) by the interleukin-1 receptor associated kinase 4 inhibitor (IRAK4i) I92 and hydroxychloroquine (HCQ). RNA-sequencing analysis was performed on RNA-IC-stimulated pDCs from healthy donors (*n* = 4), cultivated for 6 h in the presence or absence of I92 or HCQ. The heat map showing the mean log2 fold change values for the 975 activation signature genes from RNA-IC versus mock-stimulated cells (left), RNA-IC I92 versus RNA-IC (middle left), RNA-IC HCQ versus RNA-IC (middle right), and the difference between the two compounds RNA-IC I92 versus RNA-IC HCQ treated cultures (right)

**Fig. 6:**
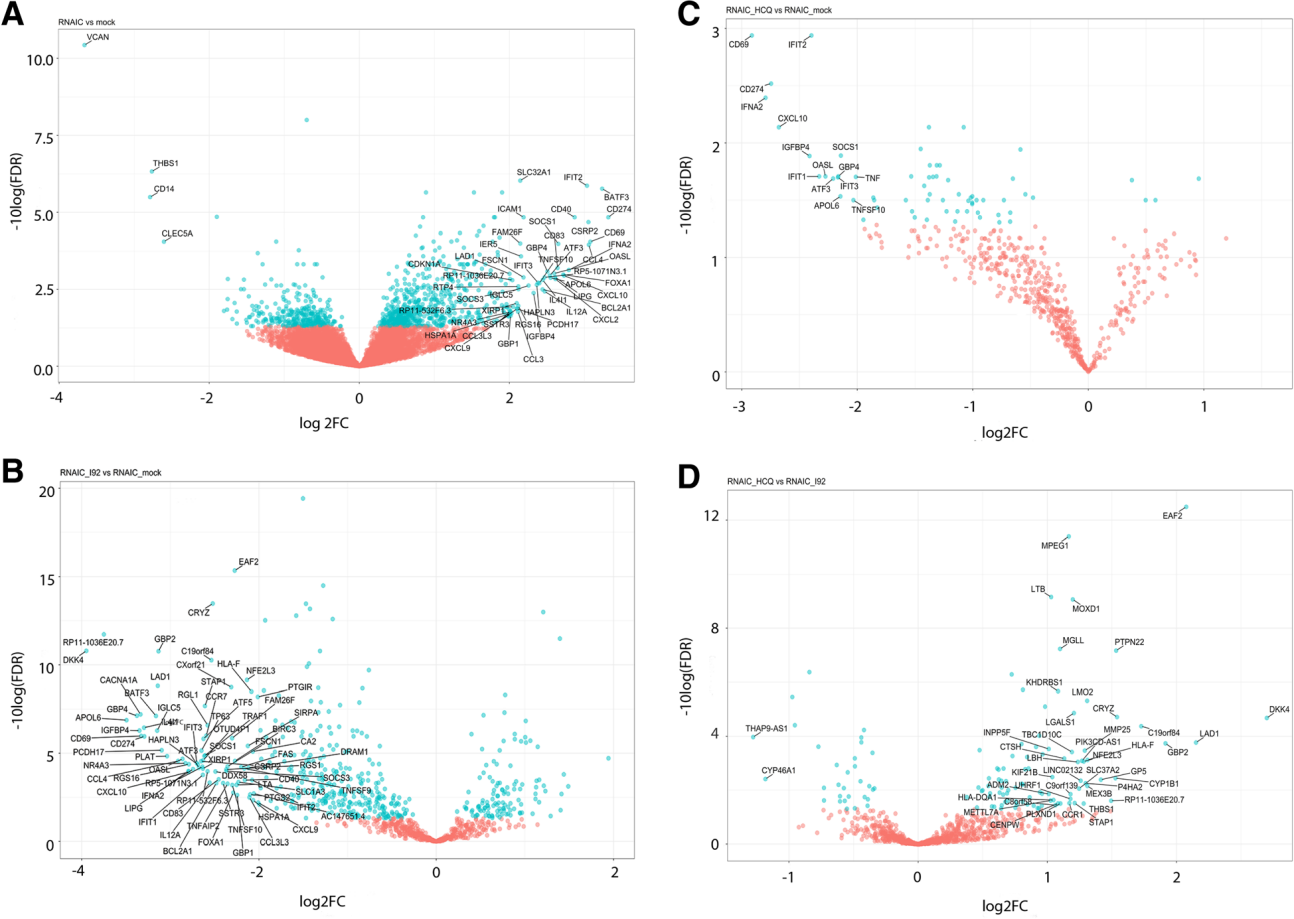
The effect of interleukin-1 receptor associated kinase 4 inhibitor (IRAK4i) I92 or hydroxychloroquine (HCQ) treatment on gene expression in plasmacytoid dendritic cells (pDCs) stimulated with RNA-containing immune complex (RNA-IC). RNA sequencing analysis was performed on RNA-IC-stimulated pDCs from healthy donors (*n* = 4), cultivated for 6 h in the presence or absence of I92 or HCQ. Volcano plots comparing false discovery rate (FDR) versus log2 fold change (FC) for genes from **a** RNA-IC-stimulated pDCs relative to mock-treated cells, **b** RNA-IC I92 relative to RNA-IC-treated cells, **c** RNA-IC HCQ relative to RNA-IC-treated cells, or **d** RNA-IC HCQ relative to RNA-IC I92-treated cells. Genes labeled in blue are statistically significantly different between the two treatments (FDR < 0.05)

To clarify the pathways affected by I92 and HCQ, we performed transcriptome sequencing (RNA-seq) of RNA-IC-stimulated pDCs. An RNA-IC activation signature was identified consisting of 975 differentially expressed genes (DEGs) compared with unstimulated pDCs (RNA-IC-DEGs, FDR < 0.05; Fig. 5, Additional file 7). A majority of the responding genes showed an upregulation after RNA-IC stimulation (*n* = 670), with 48 genes increased at least fourfold (Fig. 6a, Additional file 7). Among the 48 top RNA-IC-DEGs were genes mapping to the type I IFN signaling pathway (*IFNA2*, *IFIT1–3*, *GBP1*, and *OASL*), clearance of apoptotic material (*BCL2A1*, *CDKN1A*, and *TNFSF10*), and chemokine genes (*CXCL2*, *CXCL9*, *CXCL10*, and *CCL4*). Several regulators of inflammation and activation (e.g., *IRF1*, *IRF3*, *STAT1*, *STAT3*, *NFκB*, *RELA/B*, and *SP1*) were predicted to drive the RNA-IC-DEGs (Additional file 8).

I92 significantly altered the expression of almost 4000 genes (FDR < 0.05) in RNA-IC-activated pDCs, of which 492 overlapped with the RNA-IC-DEGs. In contrast, HCQ significantly altered only 73 genes, with 65 overlapping with the RNA-IC-DEGs (FDR < 0.05) (Fig. 5, Additional file 9). More RNA-IC-DEGs were strongly downregulated (log2 fold change > 2) by I92 (*n* = 73) than by HCQ (*n* = 15) (Fig. 6b, c). The expression of several top upregulated genes in the RNA-IC-DEGs was reversed by both I92 and HCQ, including *IFNA2*, *IFIT2–3*, *OASL*, *CXCL10*, *CD274*, *TNFSF10*, and *APOL6*.

Between HCQ- and I92-treated, RNA-IC-stimulated pDCs, 125 genes were differentially expressed. The greatest relative difference in expression was observed for *DKK4*, *LAD1*, and *EAF2* (Fig. 6d), which were more strongly downregulated by I92 than by HCQ. These genes have mainly been studied in the context of tumorigenesis [23–25]. The top enriched biological function pathway for these genes was macroautophagy decline, represented by *ATG14*, *AMBRA1*, and *BECN1*, contributing to the regulation of autophagy, autophagosomal maturation, endocytosis, and apoptosis (Additional file 10). Several STAT-related signaling pathways were more suppressed by I92 than by HCQ (Additional file 11). The expression of cytokine genes *TNF*, *IFNA2*, *CXCL8*, *CCL3*, and *CCL4* was significantly suppressed by I92, compared with only *TNF* and *IFNA2* being suppressed by HCQ (Additional file 12). In conclusion, both I92 and HCQ reversed the effects of RNA-IC stimulation on the pDC gene expression profile, but I92 more extensively affected gene expression and modulated more cellular pathways than HCQ.

## Discussion

This study demonstrates that the cytokine production by RNA-IC-stimulated pDCs and NK cells can be suppressed by HCQ and, more profoundly, by an IRAK4 inhibitor. The strong TNF-α induction by RNA-IC is interesting since TNF-α plays a critical role in several SLE disease manifestations, such as nephritis, skin lesions, and arthritis, all characterized by tissue deposition of ICs [26–28]. Increased IC formation precedes SLE flares and our findings may therefore partly explain the observed association between increased serum TNF-α levels and disease activity in SLE [29]. The difference in the TNF-α production rate between pDCs and NK cells indicates that RNA-ICs activate different induction pathways for TNF-α synthesis in these two cell types. Supporting this conjecture is the observed difference between pDCs and NK cells in response to HCQ. pDCs are mainly activated by ligation of endosomal TLRs [2] and this pathway is inhibited by HCQ [7]. TNF-α production by NK cells, on the other hand, can be induced by a number of different receptors, including TLR7 [30–32]. However, RNA-IC-induced production of cytokines and chemokines from NK cells was not dependent on endosomal TLR signaling since HCQ had no inhibitory effect. Consistent with a TLR7-independent activation of NK cells, heat-aggregated IgG was as efficient as RNA-IC in inducing TNF-α from purified NK cells, and a synthetic TLR7 agonist (DSR6434) did not induce TNF-α in NK cells (Additional file 13). Although no studies were performed regarding specific RNA-IC-responding NK cell receptors, the fact that TNF-α production was observed only in the CD56^dim^, CD16-expressing NK cell population (Additional file 14) suggests NK cell activation by RNA-IC via CD16. The prominent effect of the IRAK4 inhibitor I92 on the TNF-α production by NK cells implies that NFκB-mediated and/or mitogen-activated protein kinase activation was involved in the NK cell response [33]. However, we cannot exclude that other protein kinases were also affected by I92, despite the previously demonstrated high selectivity for IRAK4 by this drug [16].

When investigating the effect of HCQ and IRAK4 inhibitor I92 on other RNA-IC-induced cytokines, we noted that HCQ almost completely blocked the production of all investigated cytokines by pDCs. This was in stark contrast to the lack of effect on the cytokine response in NK cells. Conversely, HCQ markedly reduced the production of most cytokines in the pDC/NK cell cocultures. The reason for this is unclear, but an optimal cytokine production in cell cocultures depends on both cell types since RNA-IC-activated pDC and NK cells promote the function of each other [6]. Consequently, inhibition of the pDC function in pDC/NK cell cocultures will also reduce the NK cell cytokine-producing capacity. However, in pDC/NK cocultures the production of IFN-γ and MIP-1β was not affected by HCQ, suggesting a pDC-independent production by NK cells, although the exact cellular source of these cytokines was not investigated. Nevertheless, this observation indicates the need for a therapeutic agent with broader effects than HCQ to achieve better control of IC-driven inflammatory processes.

The IRAK4 inhibitor I92 blocked the NK cell production of all cytokines in healthy individuals, except for IL-8. This could imply yet another induction mechanism for IL-8 production in NK cells. In fact, IL-8 production was also remarkably high in monocyte-depleted PBMC cultures from SLE patients (Additional file 15) but, due to a shortage of patient material, the effects of I92 and HCQ on the IL-8 production could not be clarified. Studies have shown that patients with SLE have increased serum levels of IL-8 despite continuous standard treatment and being in remission [34]. An association between IL-8 gene polymorphisms and SLE further supports a role for IL-8 in SLE [35]. Additional studies are needed to determine the regulation of IL-8 production in patients with SLE, and some are now in progress. Notably, I92 inhibited all other investigated cytokines produced by RNA-IC-stimulated cells from SLE patients, whereas HCQ only reduced IL-6 and MIP1-β production significantly.

The RNA-IC activation signature in pDCs revealed an enrichment of pathways with connection to the IFN signaling system, antigen presentation, and apoptosis. This demonstrates that nucleic acid containing ICs elicit a powerful inflammatory response, but also trigger other cellular processes of importance in SLE. Both I92 and HCQ largely reversed the RNA-IC activation in pDCs, although some differences were observed. I92 increased the expression of genes involved in protein degradation and the autophagy process, in contrast to HCQ which downregulated these genes. The most strongly downregulated genes by I92 compared with HCQ were *DKK4*, *LAD1*, and *EAF2*, and suppression of these genes could have several effects on the SLE disease process. DKK4 is an inhibitor of the canonical Wnt signaling pathway, which has been suggested to contribute to disrupted T effector cell differentiation and the immune dysfunction in SLE [36, 37]. Ladinin 1, encoded by *LAD1*, modulates the EGF to ERK pathway and increased ERK activation is associated with organ damage in SLE [24, 38]. EAF2, on the other hand, is selectively upregulated in germinal center B cells and promotes their apoptosis [39]. Possibly, inhibition of EAF2 could therefore increase autoantibody production. The increased activation of the autophagy pathway by I92 might be beneficial in SLE since autophagy is reduced in SLE regulatory T cells and enhanced autophagy has been shown to improve both murine and human SLE [40]. On the other hand, activation of autophagy favors plasmablast development, enabling expansion of self-reactive B cells in SLE, as well as type I IFN production by facilitating intracellular IC transport [41, 42]. These observations merit further studies of the effects of I92 on different cell types, not least considering that IRAK4 inhibition ameliorates experimental murine lupus, suggesting a favorable effect also in human SLE [43]. Translating results of in-vitro studies of pharmaceutical compounds to potential drug effects in vivo has limitations. However, the approach to investigate drug candidates in cell cultures can be useful to determine the effects on central immune cells in the disease process [44]. Thus, we consider our system with IC-stimulated immune cells from SLE patients as one relevant model for an initial screening of potential drugs that target disease-associated pathways in SLE.

## Conclusions

In conclusion, the IRAK4 inhibitor I92 reduced a number of proinflammatory cytokines triggered by RNA-IC that are involved in the immune pathogenesis of SLE equally, or more effectively, than HCQ. For the first time, we show the effects of an IRAK4 inhibitor on both transcription and protein synthesis in RNA-IC-activated pDCs, which demonstrates that IRAK4 inhibition affects many cellular pathways of importance in an autoimmune disease process.

## Additional files

Additional file 1: **Table S1.** Patient clinical characteristics. (PDF 36 kb)

Additional file 2: **Figure S1.** Titration of IRAK4 inhibitor (I92) on cytokine production by cocultured plasmacytoid dendritic cells and NK cells. (PDF 194 kb)

Additional file 3: **Figure S2.** Titration of hydroxychloroquine in cocultured plasmacytoid dendritic cells and NK cells with regard to interferon-α production. (PDF 126 kb)

Additional file 4: **Figure S3.** Titration of hydroxychloroquine in cocultured plasmacytoid dendritic cells and NK cells. (PDF 221 kb)

Additional file 5: **Figure S4.** Flow cytometric gating strategy of stimulated plasmacytoid dendritic cells and NK cells. (PDF 211 kb)

Additional file 6: **Figure S5.** Flow cytometry showing total proportion of cytokine-producing cells in RNA-IC-stimulated pDC and NK cells. (PDF 282 kb)

Additional file 7: **Table S2.** Gene list of 975 differentially expressed genes. (PDF 767 kb)

Additional file 8: **Table S3.** Upstream regulators. (PDF 291 kb)

Additional file 9: **Figure S6.** Overlap of differentially expressed genes in plasmacytoid dendritic cells. (PDF 135 kb)

Additional file 10: **Table S4.** Enriched biological function pathways. (PDF 249 kb)

Additional file 11: **Table S5.** Enriched signal processing pathways. (PDF 251 kb)

Additional file 12: **Figure S7.** RNA-seq analysis of cytokine expression in plasmacytoid dendritic cells stimulated for 6 h in the presence of IRAK4 inhibitor or hydroxychloroquine. (PDF 186 kb)

Additional file 13: **Figure S8.** TNF-α production in NK cell cultures and NK cell/pDC cocultures. (PDF 179 kb)

Additional file 14: **Figure S9.** Flow cytometric analysis of TNF-α in NK cells. (PDF 165 kb)

Additional file 15: **Figure S10.** Interleukin-8 production by stimulated blood cells from SLE patients. (PDF 104 kb)

Additional file 16: **Table S6.** Gene expression in plasmacytoid dendritic cells (pDCs) from healthy donors. (XLSX 4030 kb)

## Abbreviations

*AMBRA1*: Autophagy and beclin 1 regulator 1
APC: Allophycocyanin
*APOL6*: Apolipoprotein L6
*ATG14*: Autophagy related 14
*BCL2A1*: BCL2 related protein A1
BDCA: Blood dendritic cell antigen
*BECN1*: Beclin 1
*CCL*: C-C motif chemokine ligand
CD: Cluster of differentiation
*CD274*: CD274 molecule
*CDKN1A*: Cyclin dependent kinase inhibitor 1A
*CXCL*: C-X-C motif chemokine ligand
DEG: Differentially expressed gene
DELFIA: Dissociation-enhanced lanthanide fluorescence immunoassay
*DKK4*: Dickkopf WNT signaling pathway inhibitor 4
*EAF2*: ELL associated factor 2
FcγRIIA: Fragment crystallizable receptor IIA
FDR: False discovery rate
FITC: Fluorescein isothiocyanate
HCQ: Hydroxychloroquine
IC: Immune complex
*IFIT*: Interferon induced protein with tetratricopeptide repeats
IFN: Interferon
*IFNA2*: Interferon alpha 2
Ig: Immunoglobulin
IL: Interleukin
IRAK: Interleukin-1 receptor-associated kinase
IRAK4i: Interleukin-1 receptor-associated kinase 4 inhibitor
IRF: Interferon regulatory factor
*LAD1*: Ladinin 1
LLoQ: Lower limits of quantification
mAb: Monoclonal antibody
MIP: Macrophage inflammatory protein
MyD88: Myeloid differentiation primary response protein 88
NFκB: Nuclear factor kappa-light-chain-enhancer of activated B cells
NK: Natural killer
*OASL*: 2′-5′-Oligoadenylate synthetase like protein
PBMC: Peripheral blood mononuclear cell
pDC: Plasmacytoid dendritic cell
PE: Phycoerythrin
*RELA*: RELA proto-oncogene, NF-κB subunit
*RELB*: RELB proto-oncogene, NF-κB subunit
RIN: RNA integrity number
RNA-IC: Ribonucleic acid containing immune complex
RNP: Ribonucleoprotein
SLE: Systemic lupus erythematosus
Sm: Smith nuclear antigen
snRNP: Small nuclear ribonucleoprotein
*SP1*: Specificity protein 1 transcription factor
*STAT*: Signal transducer and activator of transcription
TLR: Toll-like receptor
TNF: Tumor necrosis factor
*TNFSF10*: TNF superfamily member 10
