# Supplementary material for: Cytokine production by activated plasmacytoid dendritic cells and natural killer cells is suppressed by an IRAK4 inhibitor
Source: Arthritis Res Ther. 2018 Oct 24;20:238. doi: 10.1186/s13075-018-1702-0 (PMC6235225; doi:10.1186/s13075-018-1702-0)
Supplement: Supplementary file 1 — Table S1. Patient clinical characteristics. (PDF 36 kb) [file 13075_2018_1702_MOESM1_ESM.pdf]

Additional file 1. Patient clinical characteristics

Table S1 Clinical characteristics of SLE patients and their treatment at the time of blood sampling.

**Patient characteristics**	
Female n (%)	13 (86)
Male n (%)	2 (14)
Age years, median (range)	53 (32-81)
Disease duration years, median (range)	15 (1-46)
SLEDAI 2K* 0p	9 (60)
SLEDAI 2K 1-3p	3 (20)
SLEDAI 2K >4p	3 (20)
Anti-malarials n (%)	8 (53)
Mycophenolate mophetil n (%)	4 (27)
Azathioprine n (%)	3 (20)
Prednisone n (%)	9 (60)
Methotrexate n (%)	2 (14)
No immunosuppression n (%)	2 (14)

*SLEDAI 2K SLE Disease Activity Index 2000 [1]

1. Gladman DD, Ibanez D, Urowitz MB. Systemic lupus erythematosus disease activity index 2000. *J Rheumatol* 2002;**29**:288-91.
