# Supplementary material for: Cytokine production by activated plasmacytoid dendritic cells and natural killer cells is suppressed by an IRAK4 inhibitor
Source: Arthritis Res Ther. 2018 Oct 24;20:238. doi: 10.1186/s13075-018-1702-0 (PMC6235225; doi:10.1186/s13075-018-1702-0)
Supplement: Supplementary file 2 — Figure S1. Titration of IRAK4 inhibitor (I92) on cytokine production by cocultured plasmacytoid dendritic cells and NK cells. (PDF 194 kb) [file 13075_2018_1702_MOESM2_ESM.pdf]

Additional file 2. Titration of IRAK4 inhibitor (I92) on cytokine production by co-cultured plasmacytoid dendritic cells and NK cells

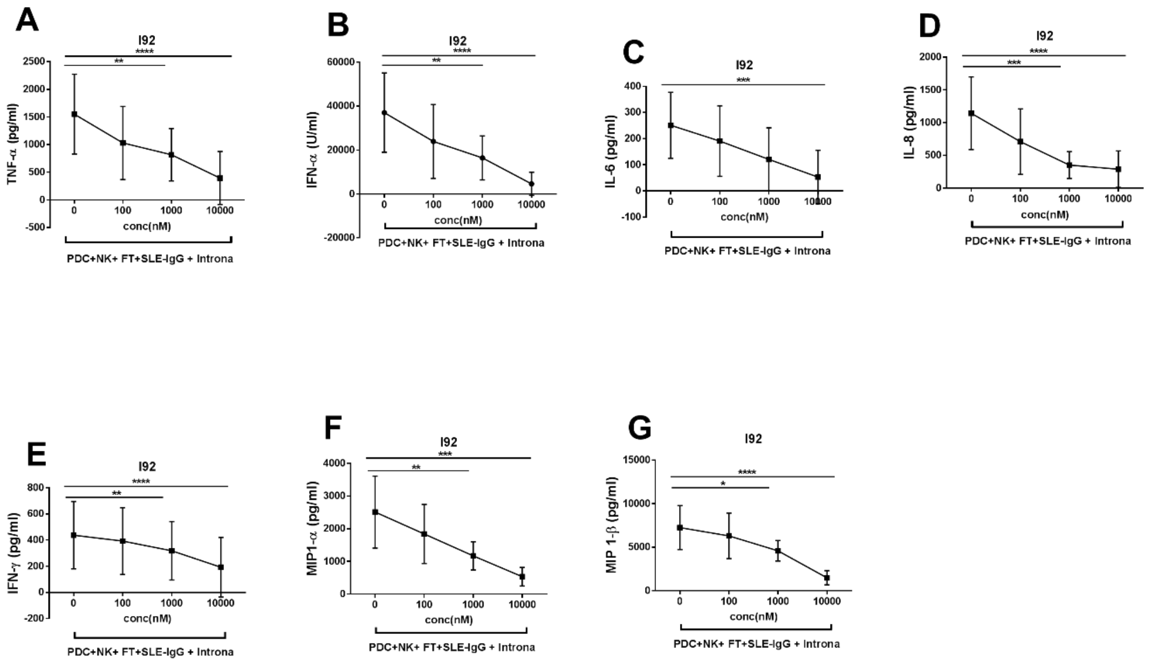

Additional figure S1. Effect of different concentrations of IRAK4 inhibitor I92 on cytokine production by activated plasmacytoid dendritic cells (pDCs) and NK cells. The cells from healthy individuals were co-cultured and stimulated with SLE-IgG, freeze-thawed necrotic cell material (FT) and supplemented with IFN-α2b (IntronA) in the presence of Interleukin 1 Receptor Associated Kinase (IRAK) 4 inhibitor I92. The cytokine production in the cell cultures were analyzed after 20 h by immunoassays. **A** significant reduction of cytokine levels was observed for (A) tumor necrosis factor (TNF) -α (B) interferon (IFN) -α (C) IL-6 (D) IL-8 (E) IFN-γ (F) macrophage inflammatory protein (MIP1)-α (G) MIP-1β when using the IRAK4 inhibitor I92 in a concentration range of 1000 to 10000 nM. Based on these results the concentration of 10000nM was chosen for further use. Data represent the mean with standard deviation based on 6 donors in two independent experiments. (Friedman´s test, Dunn’s test for multiple comparisons, * refers to $p< 0.05$, ** $p<0.01$, *** $p<0.001$ and **** $p<0.0001$)
