# Supplementary material for: Cytokine production by activated plasmacytoid dendritic cells and natural killer cells is suppressed by an IRAK4 inhibitor
Source: Arthritis Res Ther. 2018 Oct 24;20:238. doi: 10.1186/s13075-018-1702-0 (PMC6235225; doi:10.1186/s13075-018-1702-0)
Supplement: Supplementary file 3 — Figure S2. Titration of hydroxychloroquine in cocultured plasmacytoid dendritic cells and NK cells with regard to interferon-α production. (PDF 126 kb) [file 13075_2018_1702_MOESM3_ESM.pdf]

Additional file 3. Titration of hydroxychloroquine in co-cultured plasmacytoid dendritic cells and NK cells with regard to interferon-α production.

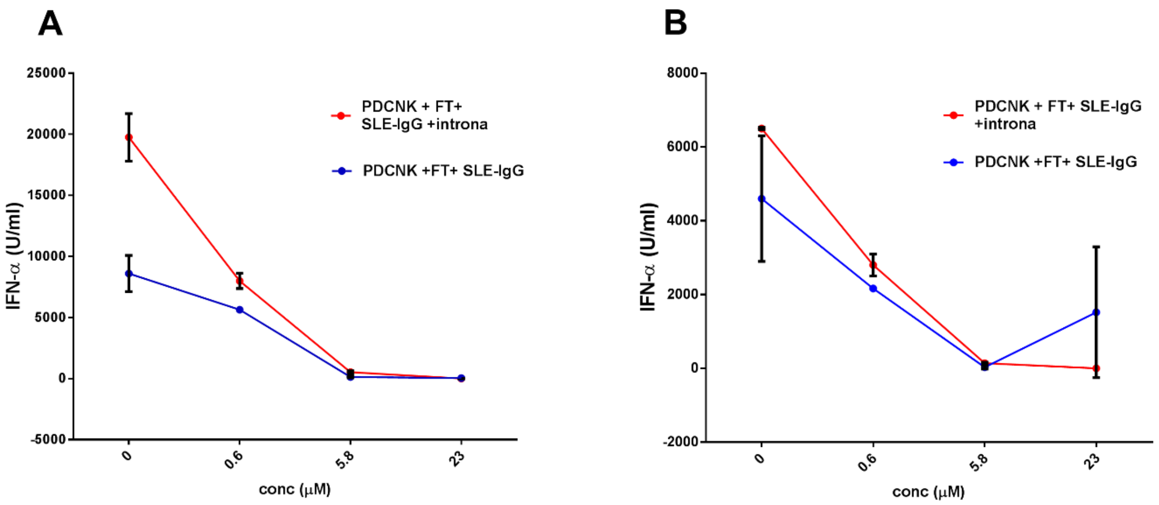

Additional figure S2. Effect of different hydroxychloroquine (HCQ) concentrations on interferon (IFN)-α production by plasmacytoid dendritic cells (pDCs) co-cultured with NK cells and stimulated with SLE-IgG and freeze-thawed necrotic cell material (FT). The graphs show IFN-α levels in 20h in cell culture supernatants from co-cultured pDCs and NK cells from two healthy donors (A, B). Red and blue lines represent IFN-α production in the cultures supplemented with and without 500 U/ml IFN-α2b (IntronA), respectively. Complete inhibition by HCQ was reached at a concentration of 5.8 μM. Data represent the mean with standard deviation based on duplicate values for each donor.
