# Supplementary material for: Cytokine production by activated plasmacytoid dendritic cells and natural killer cells is suppressed by an IRAK4 inhibitor
Source: Arthritis Res Ther. 2018 Oct 24;20:238. doi: 10.1186/s13075-018-1702-0 (PMC6235225; doi:10.1186/s13075-018-1702-0)
Supplement: Supplementary file 4 — Figure S3. Titration of hydroxychloroquine in cocultured plasmacytoid dendritic cells and NK cells. (PDF 221 kb) [file 13075_2018_1702_MOESM4_ESM.pdf]

Additional file 4. Titration of hydroxychloroquine in co-cultured plasmacytoid dendritic cells and NK cells

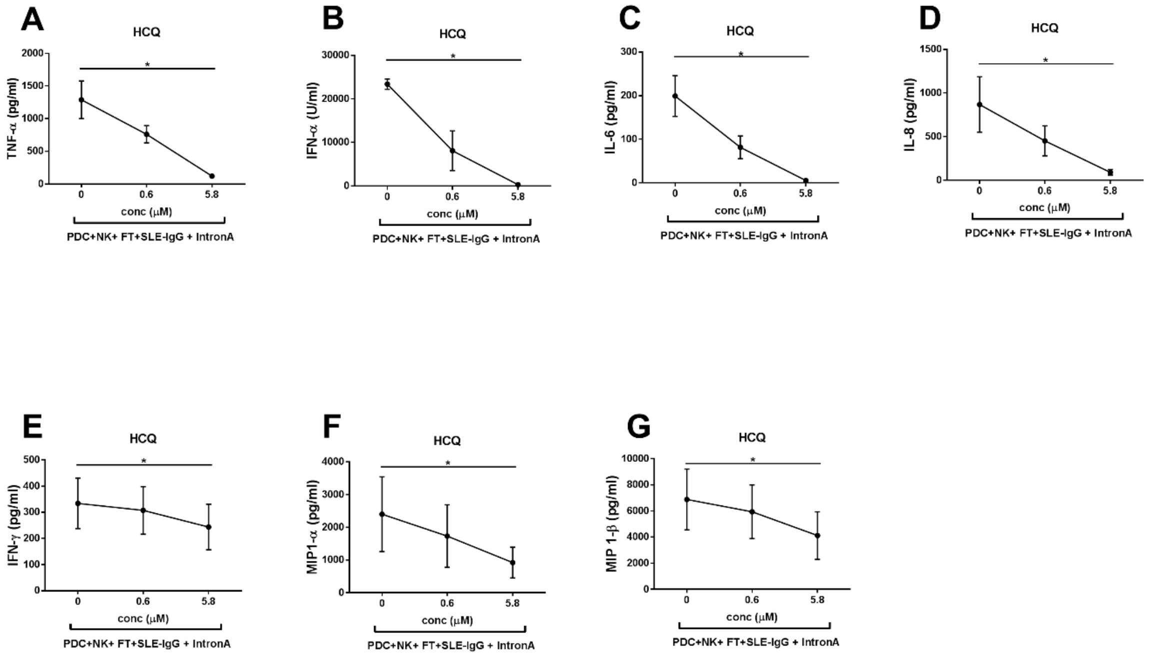

Additional figure S3. Effect of different concentrations of hydroxychloroquine (HCQ) on cytokine production by activated plasmacytoid dendritic cells (pDCs) and NK cells. The cells from healthy individuals were stimulated with SLE-IgG, freeze-thawed necrotic cell material and supplemented with IFN-α2b (IntronA) in the presence of three concentrations of HCQ. The cytokine production in the cell cultures was analyzed after 20 h by immunoassays. A significant reduction of cytokine levels was observed for (A) TNF-α (B) IFN-α (C) IL-6 (D) IL-8 (E) IFN-γ (F) macrophage inflammatory protein (MIP)1-α (G) MIP-1β when the cultures were supplemented with 5.8 µM HCQ. Data represent the mean with standard deviation based on 3 donors from one experiment. (Friedman´s test, Dunn’s test for multiple comparisons, * refers to $p < 0.05$)
