# Supplementary material for: Cytokine production by activated plasmacytoid dendritic cells and natural killer cells is suppressed by an IRAK4 inhibitor
Source: Arthritis Res Ther. 2018 Oct 24;20:238. doi: 10.1186/s13075-018-1702-0 (PMC6235225; doi:10.1186/s13075-018-1702-0)
Supplement: Supplementary file 5 — Figure S4. Flow cytometric gating strategy of stimulated plasmacytoid dendritic cells and NK cells. (PDF 211 kb) [file 13075_2018_1702_MOESM5_ESM.pdf]

Additional file 5. Flow cytometric gating strategy of stimulated plasmacytoid dendritic cells and NK cells

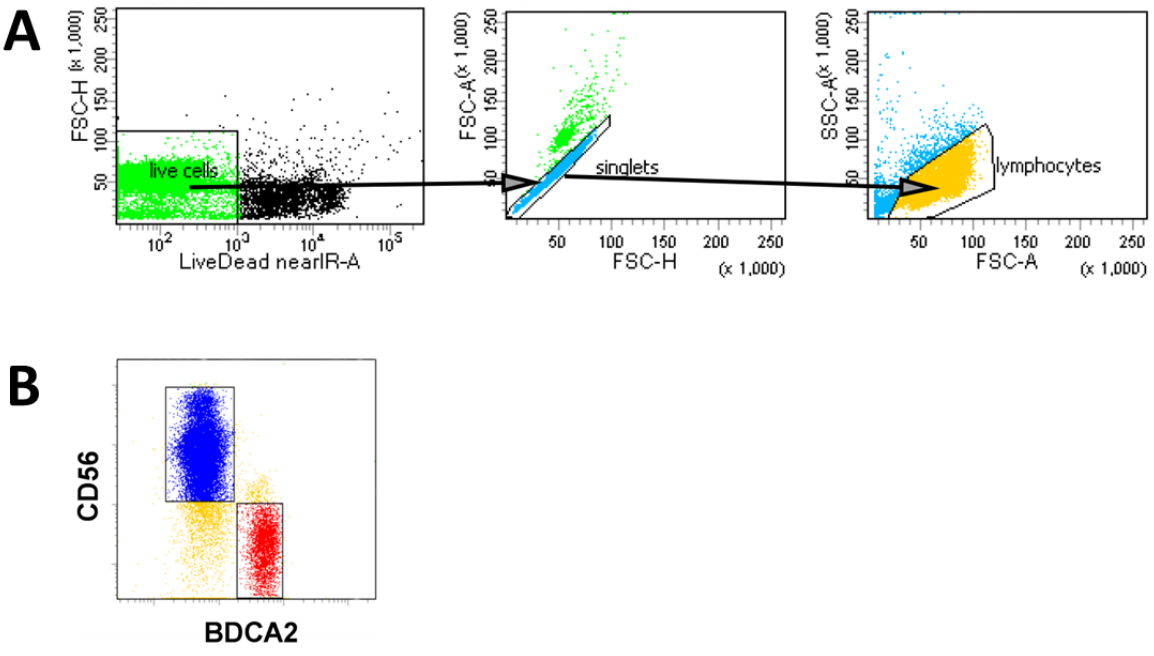

Additional figure S4. Plasmacytoid dendritic cells (pDCs) and NK cells stimulated in co-cultures with RNA-containing immune complexes (RNA-IC). (A) Cells were consecutively gated for live cells, singlets and lymphocytes. (B) Cells were stained with monoclonal antibodies to blood dendritic cell antigen (BDCA) 2 and CD56 and analyzed by flow cytometry to identify pDC (red) and NK cells (blue), respectively.
