# Supplementary material for: Cytokine production by activated plasmacytoid dendritic cells and natural killer cells is suppressed by an IRAK4 inhibitor
Source: Arthritis Res Ther. 2018 Oct 24;20:238. doi: 10.1186/s13075-018-1702-0 (PMC6235225; doi:10.1186/s13075-018-1702-0)
Supplement: Supplementary file 6 — Figure S5. Flow cytometry showing total proportion of cytokine-producing cells in RNA-IC-stimulated pDC and NK cells. (PDF 282 kb) [file 13075_2018_1702_MOESM6_ESM.pdf]

Additional file 6. Flow cytometry showing total proportion of cytokine producing cells and summary flow data of RNA-IC stimulated pDC and NK cells.

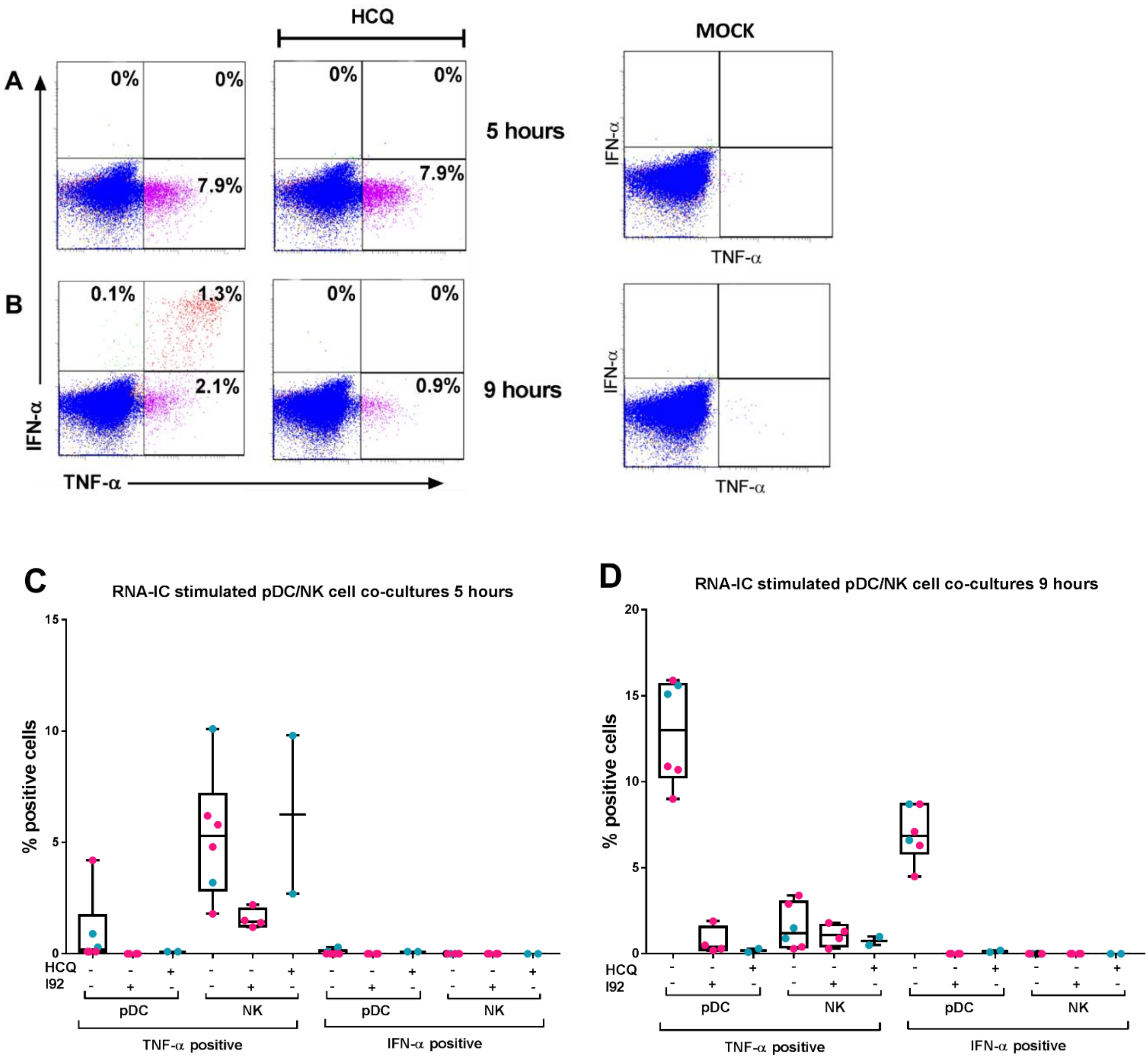

Additional figure S5. pDC and NK cells were isolated from healthy blood donor PBMCs and stimulated in co-cultures with RNA containing immune complexes (RNA-IC) and analyzed by flow cytometry. Figures show total proportion of TNF-α (lower right), IFN-α (upper left), IFN-α and TNF-α (upper right) positive cells at (A) five, and (B) nine hours in the lymphocyte gate (see add. file 5) Middle column shows HCQ treated, and right column unstimulated cell cultures (mock). Data from one representative of at least two individual donors. (C, D) Proportion (%) of TNF-α and IFN-α positive pDC and NK cells, at 5 and 9 hours. Each dot represents cells from one individual donor treated with and without HCQ (blue, n=2) and I92 (pink, n=4) respectively. Boxplots show median with interquartile range. For gating strategy please see additional file 5.
