# Supplementary material for: Cytokine production by activated plasmacytoid dendritic cells and natural killer cells is suppressed by an IRAK4 inhibitor
Source: Arthritis Res Ther. 2018 Oct 24;20:238. doi: 10.1186/s13075-018-1702-0 (PMC6235225; doi:10.1186/s13075-018-1702-0)
Supplement: Supplementary file 7 — Table S2. Gene list of 975 differentially expressed genes. (PDF 767 kb) [file 13075_2018_1702_MOESM7_ESM.pdf]

Additional file 7 Gene list 975 differentially expressed genes

Table S2. Gene list 975 differentially expressed genes (DEGs) in pDCs stimulated by RNA-IC vs mock stimulated pDCs (FDR<0.05). Effects of I92 and HCQ on gene expression in RNA-IC stimulated pDCs. Chr; chromosome, FC; Fold Change (of gene expression between compared conditions), padj; adjusted p-value, FDR. *FDR; False Discovery Rate, Benjamini Hochberg. List sorted in declining order according to log2FC of gene expression in RNA-IC compared to mock treated pDCs.

EnsemblGeneID	Gene, name	Chr	log2FC RNAIC vs MOCK	padj RNAIC vs MOCK	log2FC RNAIC_I92 vs RNAIC	padj RNAIC_I92 vs RNAIC	log2FC RNAIC_HCQ vs RNAIC	padj RNAIC_HCQ vs RNAIC	Gene, description	Gene, type
ENSG00000120217	CD274	9	3.32	1.46E-05	-3.29	1.11E-06	-2.75	3.02E-03	CD274 molecule	protein coding
ENSG00000123685	BATF3	1	3.23	1.71E-06	-3.16	7.64E-08	-1.89	NA	basic leucine zipper ATF-like transcription factor 3	protein coding
ENSG00000110848	CD69	12	3.08	8.85E-05	-3.33	1.05E-06	-2.91	1.15E-03	CD69 molecule	protein coding
ENSG00000275302	CCL4	17	3.06	1.11E-04	-2.91	2.76E-05	-1.89	0.06	C-C motif chemokine ligand 4	protein coding
ENSG00000175183	CSRP2	12	3.05	2.09E-05	-2.65	2.85E-05	-2.03	NA	cysteine and glycine rich protein 2	protein coding
ENSG00000119922	IFIT2	10	3.03	1.37E-06	-2.08	3.31E-04	-2.40	1.15E-03	interferon induced protein with tetratricopeptide repeats 2	protein coding
ENSG00000101017	CD40	20	2.87	1.46E-05	-2.16	3.34E-04	-1.86	0.03	CD40 molecule	protein coding
ENSG00000188379	IFNA2	9	2.79	7.37E-04	-2.80	1.02E-04	-2.79	4.02E-03	interferon alpha 2	protein coding
ENSG00000169245	CXCL10	4	2.73	1.12E-03	-2.79	1.15E-04	-2.68	0.01	C-X-C motif chemokine ligand 10	protein coding
ENSG00000129514	FOXA1	14	2.72	1.04E-03	-2.29	1.73E-03	-2.31	NA	forkhead box A1	protein coding
ENSG00000185338	SOCS1	16	2.65	1.04E-04	-2.61	1.33E-05	-2.14	0.01	suppressor of cytokine signaling 1	protein coding
ENSG00000112149	CD83	6	2.64	6.33E-04	-2.45	3.03E-04	-1.95	0.05	CD83 molecule	protein coding
ENSG00000081041	CXCL2	4	2.61	1.45E-03	-1.23	0.12	-1.56	NA	C-X-C motif chemokine ligand 2	protein coding
ENSG00000101670	LIPG	18	2.61	1.27E-03	-2.74	7.99E-05	-2.30	NA	lipase G, endothelial type	protein coding
ENSG00000135114	OASL	12	2.60	9.55E-04	-2.86	2.10E-05	-2.28	0.02	2'-5'-oligoadenylate synthetase like	protein coding
ENSG00000221963	APOL6	22	2.55	1.32E-03	-3.49	1.33E-07	-2.15	0.03	apolipoprotein L6	protein coding
ENSG00000162772	ATF3	1	2.54	1.12E-03	-2.68	6.75E-05	-2.21	0.02	activating transcription factor 3	protein coding
ENSG00000162654	GBP4	1	2.51	8.25E-04	-3.38	7.43E-08	-2.16	0.02	guanylate binding protein 4	protein coding
ENSG00000168811	IL12A	3	2.47	3.66E-03	-2.56	4.68E-04	-2.55	NA	interleukin 12A	protein coding
ENSG00000231105	RP5-1071N3,1	1	2.47	1.28E-03	-2.64	5.81E-05	-2.07	NA		antisense
ENSG00000104951	IL4I1	19	2.44	1.55E-03	-3.30	3.54E-07	-1.81	0.06	interleukin 4 induced 1	protein coding
ENSG00000140379	BCL2A1	15	2.44	3.16E-03	-2.48	4.97E-04	-1.88	0.07	BCL2 related protein A1	protein coding
ENSG00000121858	TNFSF10	3	2.40	1.98E-03	-2.30	6.41E-04	-2.03	0.03	tumor necrosis factor superfamily member 10	protein coding
ENSG00000119917	IFIT3	10	2.38	1.92E-03	-2.70	3.56E-05	-2.17	0.02	interferon induced protein with tetratricopeptide repeats 3	protein coding
ENSG00000140511	HAPLN3	15	2.36	2.23E-03	-2.69	4.34E-05	-1.56	0.10	hyaluronan and proteoglycan link protein 3	protein coding
ENSG00000141753	IGFBP4	17	2.32	4.12E-03	-3.35	5.19E-07	-2.41	0.01	insulin like growth factor binding protein 4	protein coding
ENSG00000254030	IGLC5	22	2.26	2.36E-03	-3.15	5.19E-07	-1.95	NA	immunoglobulin lambda constant 5 (pseudogene)	IG C pseudogene
ENSG00000090339	ICAM1	19	2.19	1.46E-05	-1.88	3.31E-05	-1.53	0.02	intercellular adhesion molecule 1	protein coding
ENSG00000075618	FSCN1	7	2.19	1.28E-03	-2.35	5.61E-05	-1.15	0.15	fascin actin-bundling protein 1	protein coding
ENSG00000162783	IER5	1	2.15	2.65E-04	-1.60	2.51E-03	-1.58	0.03	immediate early response 5	protein coding
ENSG00000188820	FAM26F	6	2.14	1.02E-04	-2.30	1.40E-06	-1.38	NA	family with sequence similarity 26 member F	protein coding
ENSG00000101438	SLC32A1	20	2.14	9.34E-07	-1.71	1.28E-05	-1.05	0.07	solute carrier family 32 member 1	protein coding
ENSG00000184557	SOCS3	17	2.12	3.11E-03	-2.37	1.03E-04	-1.83	0.04	suppressor of cytokine signaling 3	protein coding
ENSG00000143333	RGS16	1	2.12	0.01	-2.79	4.20E-05	-2.51	NA	regulator of G-protein signaling 16	protein coding
ENSG00000136514	RTP4	3	2.12	2.52E-03	-1.98	9.40E-04	-2.11	NA	receptor transporter protein 4	protein coding
ENSG00000277632	CCL3	17	2.11	0.02	-2.00	0.01	-1.45	NA	C-C motif chemokine ligand 3	protein coding
ENSG00000276085	CCL3L3	17	2.10	0.01	-2.11	3.18E-03	-1.73	NA	C-C motif chemokine ligand 3 like 3	protein coding
ENSG00000168334	XIRP1	3	2.10	0.01	-2.63	7.43E-05	-1.81	NA	xin actin binding repeat containing 1	protein coding

ENSG00000118946	PCDH17	13	2.10	0.01	-3.10	6.77E-06	-2.72	NA	protocadherin 17	protein coding
ENSG00000272463	RP11-532F6,3	6	2.05	0.01	-2.58	1.06E-04	-1.41	NA		lincRNA
ENSG00000124762	CDKN1A	6	2.04	1.58E-03	-1.62	4.90E-03	-1.39	0.08	cyclin dependent kinase inhibitor 1A	protein coding
ENSG00000204389	HSPA1A	6	2.03	0.02	-2.07	4.60E-03	-1.92	NA	heat shock protein family A (Hsp70) member 1A	protein coding
ENSG00000117228	GBP1	1	2.01	0.02	-2.25	2.37E-03	-1.91	NA	guanylate binding protein 1	protein coding
ENSG00000278195	SSTR3	22	2.01	0.02	-2.40	4.95E-04	-2.12	NA	somatostatin receptor 3	protein coding
ENSG00000138755	CXCL9	4	2.01	0.02	-2.01	0.01	-1.79	0.08	C-X-C motif chemokine ligand 9	protein coding
ENSG00000119508	NR4A3	9	2.01	0.02	-2.83	3.68E-05	-1.94	0.06	nuclear receptor subfamily 4 group A member 3	protein coding
ENSG00000254704	RP11-1036E20,7	11	2.01	1.45E-03	-3.75	1.89E-12	-2.26	NA		processed pseudogene
ENSG00000159166	LAD1	1	2.00	9.80E-04	-3.14	1.56E-09	-0.99	NA	ladinin 1	protein coding
ENSG00000185215	TNFAIP2	14	2.00	0.01	-2.35	5.39E-04	-1.84	0.06	TNF alpha induced protein 2	protein coding
ENSG00000185745	IFIT1	10	1.99	0.02	-2.63	1.70E-04	-2.33	0.02	interferon induced protein with tetratricopeptide repeats 1	protein coding
ENSG00000163273	NPPC	2	1.99	0.02	-1.35	0.09	-1.94	NA	natriuretic peptide C	protein coding
ENSG00000104368	PLAT	8	1.98	0.02	-3.04	1.46E-05	-1.69	NA	plasminogen activator, tissue type	protein coding
ENSG00000158050	DUSP2	2	1.97	0.01	-1.87	4.96E-03	-1.28	NA	dual specificity phosphatase 2	protein coding
ENSG00000107201	DDX58	9	1.96	0.01	-2.49	1.09E-04	-1.83	0.05	DExD/H-box helicase 58	protein coding
ENSG00000118976	OTUD4P1	12	1.96	0.01	-2.63	2.55E-05	-1.60	NA	OTUD4 pseudogene 1	processed pseudogene
ENSG00000126353	CCR7	17	1.95	2.13E-03	-2.59	1.01E-06	-1.51	0.06	C-C motif chemokine receptor 7	protein coding
ENSG00000073756	PTGS2	1	1.93	0.02	-2.10	2.20E-03	-2.04	NA	prostaglandin-endoperoxide synthase 2	protein coding
ENSG00000079215	SLC1A3	5	1.92	0.01	-2.23	4.37E-04	-1.17	NA	solute carrier family 1 member 3	protein coding
ENSG00000226979	LTA	6	1.92	0.02	-2.25	6.22E-04	-1.82	NA	lymphotoxin alpha	protein coding
ENSG00000169136	ATF5	19	1.91	3.77E-03	-2.63	1.52E-06	-1.47	0.07	activating transcription factor 5	protein coding
ENSG00000125347	IRF1	5	1.90	2.27E-06	-1.42	1.31E-04	-1.38	0.01	interferon regulatory factor 1	protein coding
ENSG00000056558	TRAF1	9	1.90	0.01	-2.62	1.42E-05	-1.55	0.08	TNF receptor associated factor 1	protein coding
ENSG00000143344	RGL1	1	1.89	1.58E-03	-2.58	2.56E-07	-1.50	0.04	ral guanine nucleotide dissociation stimulator like 1	protein coding
ENSG00000102096	PIM2	X	1.87	6.51E-05	-0.67	0.16	-1.17	0.06	Pim-2 proto-oncogene, serine/threonine kinase	protein coding
ENSG00000109320	NFKB1	4	1.85	2.48E-04	-1.62	2.78E-04	-1.42	0.02	nuclear factor kappa B subunit 1	protein coding
ENSG00000104312	RIPK2	8	1.84	1.94E-04	-1.40	1.65E-03	-1.37	0.03	receptor interacting serine/threonine kinase 2	protein coding
ENSG00000115267	IFIH1	2	1.84	0.01	-1.63	0.01	-1.46	0.07	interferon induced with helicase C domain 1	protein coding
ENSG00000260314	MRC1	10	1.83	0.03	-1.47	0.05	-1.39	NA	mannose receptor C-type 1	protein coding
ENSG00000118971	CCND2	12	1.83	0.01	-1.59	0.01	-1.85	0.03	cyclin D2	protein coding
ENSG00000100906	NFKBIA	14	1.81	1.46E-05	-0.99	0.01	-1.21	0.02	NFKB inhibitor alpha	protein coding
ENSG00000111846	GCNT2	6	1.80	0.01	-1.71	3.38E-03	-1.51	NA	glucosaminyl (N-acetyl) transferase 2, I-branching enzyme (I blood group)	protein coding
ENSG00000171855	IFNB1	9	1.80	0.04	-1.80	0.01	-1.79	0.07	interferon beta 1	protein coding
ENSG00000170340	B3GNT2	2	1.79	1.46E-05	-1.29	7.00E-04	-1.29	0.02	UDP-GlcNAc:betaGal beta-1,3-N-acetylglucosaminyltransferase 2	protein coding
ENSG00000120280	CXorf21	X	1.78	8.85E-05	-2.31	1.83E-09	-1.45	0.01	chromosome X open reading frame 21	protein coding
ENSG00000087085	ACHE	7	1.77	0.01	-1.80	9.67E-04	-0.99	NA	acetylcholinesterase (Cartwright blood group)	protein coding
ENSG00000152778	IFIT5	10	1.75	7.63E-04	-1.32	4.83E-03	-1.39	0.03	interferon induced protein with tetratricopeptide repeats 5	protein coding
ENSG00000232810	TNF	6	1.75	0.02	-1.70	0.01	-2.01	0.02	tumor necrosis factor	protein coding
ENSG00000131669	NINJ1	9	1.74	3.98E-03	-1.22	0.03	-1.31	0.08	ninjurin 1	protein coding

ENSG00000137193	PIM1	6	1.74	1.01E-03	-1.28	0.01	-1.00	0.12	Pim-1 proto-oncogene, serine/threonine kinase	protein coding
ENSG00000171223	JUNB	19	1.73	4.50E-03	-1.75	7.95E-04	-1.53	0.04	JunB proto-oncogene, AP-1 transcription factor subunit	protein coding
ENSG00000184060	ADAP2	17	1.72	0.03	-1.62	0.01	-2.05	NA	ArfGAP with dual PH domains 2	protein coding
ENSG00000164181	ELOVL7	5	1.70	0.01	-1.33	0.02	-0.91	NA	ELOVL fatty acid elongase 7	protein coding
ENSG00000141682	PMAIP1	18	1.68	4.81E-04	-1.25	3.72E-03	-1.38	0.02	phorbol-12-myristate-13-acetate-induced protein 1	protein coding
ENSG00000026103	FAS	10	1.67	0.01	-2.20	6.02E-05	-1.53	0.06	Fas cell surface death receptor	protein coding
ENSG00000146232	NFKBIE	6	1.65	2.75E-05	-1.23	6.06E-04	-1.05	0.04	NFKB inhibitor epsilon	protein coding
ENSG00000237181	AC147651,4	7	1.65	0.04	-2.06	1.94E-03	-1.15	NA		antisense
ENSG00000272941	RP11-	7	1.64	0.03	-1.64	0.01	-1.40	NA		antisense
ENSG00000110047	EHD1	11	1.63	6.74E-04	-1.03	0.02	-0.98	0.10	EH domain containing 1	protein coding
ENSG00000162623	TYW3	1	1.62	2.15E-03	-1.20	0.01	-1.12	0.08	tRNA-yW synthesizing protein 3 homolog	protein coding
ENSG00000073282	TP63	3	1.62	0.03	-2.65	7.04E-06	-1.62	NA	tumor protein p63	protein coding
ENSG00000107796	ACTA2	10	1.61	0.02	-1.04	0.09	-1.29	NA	actin, alpha 2, smooth muscle, aorta	protein coding
ENSG00000185947	ZNF267	16	1.60	2.33E-04	-1.21	1.99E-03	-1.31	0.02	zinc finger protein 267	protein coding
ENSG00000174004	NRROS	3	1.60	4.09E-05	-1.50	1.17E-05	-0.92	0.07	negative regulator of reactive oxygen species	protein coding
ENSG00000104371	DKK4	8	1.59	0.03	-3.95	1.62E-11	-1.25	NA	dickkopf WNT signaling pathway inhibitor 4	protein coding
ENSG00000165997	ARL5B	10	1.59	0.02	-1.11	0.07	-1.40	0.08	ADP ribosylation factor like GTPase 5B	protein coding
ENSG00000124145	SDC4	20	1.59	3.35E-03	-1.46	1.69E-03	-0.81	NA	syndecan 4	protein coding
ENSG00000125657	TNFSF9	19	1.59	0.02	-2.17	7.27E-05	-1.40	NA	tumor necrosis factor superfamily member 9	protein coding
ENSG00000090104	RGS1	1	1.59	0.03	-2.37	8.30E-05	-1.47	0.09	regulator of G-protein signaling 1	protein coding
ENSG00000136048	DRAM1	12	1.59	0.01	-2.12	6.70E-05	-1.44	NA	DNA damage regulated autophagy modulator 1	protein coding
ENSG00000105559	PLEKHA4	19	1.58	0.03	-1.04	0.10	-1.28	NA	pleckstrin homology domain containing A4	protein coding
ENSG00000141837	CACNA1A	19	1.57	0.04	-3.34	6.21E-08	-1.97	NA	calcium voltage-gated channel subunit alpha1 A	protein coding
ENSG00000117280	RAB29	1	1.57	1.05E-03	-1.87	4.03E-06	-1.23	0.04	RAB29, member RAS oncogene family	protein coding
ENSG00000135925	WNT10A	2	1.57	5.59E-05	-1.29	2.23E-04	-0.71	0.14	Wnt family member 10A	protein coding
ENSG00000163947	ARHGEF3	3	1.56	0.02	-1.41	0.01	-1.26	0.10	Rho guanine nucleotide exchange factor 3	protein coding
ENSG00000171522	PTGER4	5	1.56	3.90E-04	-0.89	0.03	-1.13	0.04	prostaglandin E receptor 4	protein coding
ENSG00000272269	RP11-	6	1.55	1.58E-03	-1.39	1.09E-03	-1.26	NA		antisense
ENSG00000106537	TSPAN13	7	1.53	4.25E-04	-1.30	6.96E-04	-0.94	0.08	tetraspanin 13	protein coding
ENSG00000198053	SIRPA	20	1.53	0.01	-2.13	3.84E-06	-1.32	0.05	signal regulatory protein alpha	protein coding
ENSG00000132109	TRIM21	11	1.53	4.81E-04	-1.10	0.01	-0.93	0.09	tripartite motif containing 21	protein coding
ENSG00000104856	RELB	19	1.53	2.27E-06	-1.27	1.49E-05	-0.91	0.03	RELB proto-oncogene, NF-kB subunit	protein coding
ENSG00000023445	BIRC3	11	1.51	0.03	-2.27	2.76E-05	-1.47	0.07	baculoviral IAP repeat containing 3	protein coding
ENSG00000169429	CXCL8	4	1.50	0.03	-1.41	0.02	-1.23	0.12	C-X-C motif chemokine ligand 8	protein coding
ENSG00000035720	STAP1	4	1.49	0.01	-2.61	2.09E-08	-1.40	0.05	signal transducing adaptor family member 1	protein coding
ENSG00000136867	SLC31A2	9	1.49	1.03E-03	-1.07	0.01	-1.18	NA	solute carrier family 31 member 2	protein coding
ENSG00000099968	BCL2L13	22	1.48	2.09E-03	-0.98	0.02	-1.08	0.07	BCL2 like 13	protein coding
ENSG00000135604	STX11	6	1.47	2.27E-03	-1.71	2.91E-05	-0.98	NA	syntaxin 11	protein coding
ENSG00000077150	NFKB2	10	1.47	2.70E-04	-1.35	1.58E-04	-1.12	0.03	nuclear factor kappa B subunit 2	protein coding
ENSG00000137393	RNF144B	6	1.47	1.28E-03	-1.38	5.07E-04	-1.31	0.02	ring finger protein 144B	protein coding
ENSG00000146425	DYNLT1	6	1.46	0.01	-1.49	1.27E-03	-1.19	0.07	dynein light chain Tctex-type 1	protein coding
ENSG00000149289	ZC3H12C	11	1.46	0.02	-1.92	2.40E-04	-0.98	NA	zinc finger CCCH-type containing 12C	protein coding
ENSG00000184378	ACTRT3	3	1.46	0.02	-1.55	2.38E-03	-1.23	NA	actin related protein T3	protein coding

ENSG00000003402	CFLAR	2	1.46	0.02	-1.39	0.01	-1.42	0.05	CASP8 and FADD like apoptosis regulator	protein coding
ENSG00000050344	NFE2L3	7	1.45	4.81E-04	-2.14	7.13E-10	-0.85	0.10	nuclear factor, erythroid 2 like 3	protein coding
ENSG00000095794	CREM	10	1.44	0.02	-1.63	2.23E-03	-1.23	0.10	cAMP responsive element modulator	protein coding
ENSG00000160013	PTGIR	19	1.44	4.81E-04	-2.01	6.38E-09	-1.24	NA	prostaglandin I2 (prostacyclin) receptor (IP)	protein coding
ENSG00000135148	TRAFD1	12	1.44	4.81E-04	-1.17	1.51E-03	-0.93	0.08	TRAF-type zinc finger domain containing 1	protein coding
ENSG00000166192	SENP8	15	1.43	0.04	-0.65	0.30	-0.42	NA	SUMO/sentrin peptidase family member, NEDD8 specific	protein coding
ENSG00000183386	FHL3	1	1.43	3.41E-03	-1.82	8.47E-06	-1.32	NA	four and a half LIM domains 3	protein coding
ENSG00000114737	CISH	3	1.42	1.44E-03	-1.65	1.30E-05	-1.26	NA	cytokine inducible SH2 containing protein	protein coding
ENSG00000130202	NECTIN2	19	1.41	0.01	-1.85	1.35E-05	-1.29	NA	nectin cell adhesion molecule 2	protein coding
ENSG00000103966	EHD4	15	1.41	0.03	-1.08	0.05	-1.03	0.15	EH domain containing 4	protein coding
ENSG00000272016	NA	NA	1.41	0.01	-1.38	2.89E-03	-1.02	0.11	NA	NA
ENSG00000123737	EXOSC9	4	1.41	3.28E-03	-1.51	2.27E-04	-1.34	0.02	exosome component 9	protein coding
ENSG00000116663	FBXO6	1	1.40	2.99E-03	-1.10	0.01	-0.73	0.19	F-box protein 6	protein coding
ENSG00000164611	PTTG1	5	1.39	1.38E-04	-1.64	1.57E-07	-0.77	NA	pituitary tumor-transforming 1	protein coding
ENSG00000116791	CRYZ	1	1.39	6.15E-04	-2.52	3.36E-14	-0.98	0.06	crystallin zeta	protein coding
ENSG00000160050	CCDC28B	1	1.39	4.81E-04	-1.03	4.00E-03	-0.55	0.25	coiled-coil domain containing 28B	protein coding
ENSG00000168394	TAP1	6	1.38	9.55E-04	-1.06	4.84E-03	-0.62	0.21	transporter 1, ATP binding cassette subfamily B member	protein coding
ENSG00000104267	CA2	8	1.37	0.02	-2.07	8.00E-06	-1.13	NA	carbonic anhydrase 2	protein coding
ENSG00000103642	LACTB	15	1.36	4.81E-04	-1.29	1.52E-04	-1.07	NA	lactamase beta	protein coding
ENSG00000140464	PML	15	1.36	2.36E-03	-1.28	1.04E-03	-1.08	0.05	promyelocytic leukemia	protein coding
ENSG00000117298	ECE1	1	1.36	0.01	-1.36	1.72E-03	-1.15	0.06	endothelin converting enzyme 1	protein coding
ENSG00000277443	MARCKS	6	1.35	0.03	-1.34	0.01	-0.85	NA	myristoylated alanine rich protein kinase C substrate	protein coding
ENSG00000181359	HSP90AA6P	4	1.34	0.01	-0.81	0.08	-1.23	NA	heat shock protein 90 alpha family class A member 6, pseudogene	transcribed processed pseudogene
ENSG00000124201	ZNFX1	20	1.34	4.68E-03	-1.31	1.41E-03	-1.15	0.05	zinc finger NFX1-type containing 1	protein coding
ENSG00000112096	SOD2	6	1.34	0.01	-1.16	0.01	-0.93	0.11	superoxide dismutase 2	protein coding
ENSG00000168421	RHOH	4	1.34	4.66E-05	-0.94	1.68E-03	-0.82	0.06	ras homolog family member H	protein coding
ENSG00000123975	CKS2	9	1.34	4.81E-04	-0.68	0.06	-0.75	0.11	CDC28 protein kinase regulatory subunit 2	protein coding
ENSG00000064989	CALCRL	2	1.34	0.04	-1.55	3.27E-03	-0.99	0.17	calcitonin receptor like receptor	protein coding
ENSG00000087074	PPP1R15A	19	1.33	0.04	-1.40	0.01	-1.37	0.07	protein phosphatase 1 regulatory subunit 15A	protein coding
ENSG00000100644	HIF1A	14	1.32	2.81E-03	-1.05	0.01	-1.04	0.06	hypoxia inducible factor 1 alpha subunit	protein coding
ENSG00000136810	TXN	9	1.32	2.36E-03	-1.60	1.28E-05	-0.81	0.12	thioredoxin	protein coding
ENSG00000166128	RAB8B	15	1.32	1.69E-03	-1.15	1.83E-03	-0.80	0.12	RAB8B, member RAS oncogene family	protein coding
ENSG00000229644	NAMPTP1	10	1.31	0.03	-1.11	0.03	-0.85	NA	nicotinamide phosphoribosyltransferase pseudogene 1	processed pseudogene
ENSG00000186827	TNFRSF4	1	1.31	3.25E-04	-1.78	5.35E-09	-0.97	0.03	TNF receptor superfamily member 4	protein coding
ENSG00000128203	ASPHD2	22	1.30	8.04E-05	-1.13	1.08E-04	-1.08	0.01	aspartate beta-hydroxylase domain containing 2	protein coding
ENSG00000204482	LST1	6	1.29	0.01	-0.63	0.16	-0.68	NA	leukocyte specific transcript 1	protein coding
ENSG00000008516	MMP25	16	1.29	1.03E-03	-1.94	2.82E-09	-0.65	NA	matrix metallopeptidase 25	protein coding
ENSG00000105835	NAMPT	7	1.27	0.02	-1.25	0.01	-1.08	0.09	nicotinamide phosphoribosyltransferase	protein coding
ENSG00000156675	RAB11FIP1	8	1.27	4.51E-03	-0.93	0.02	-0.88	0.10	RAB11 family interacting protein 1	protein coding
ENSG00000162645	GBP2	1	1.27	0.04	-3.13	1.69E-11	-1.22	NA	guanylate binding protein 2	protein coding

ENSG00000103522	IL21R	16	1.27	0.03	-1.93	6.37E-05	-1.01	0.14	interleukin 21 receptor	protein coding
ENSG00000123240	OPTN	10	1.26	0.01	-1.63	5.87E-05	-1.11	0.06	optineurin	protein coding
ENSG00000197329	PELI1	2	1.26	8.46E-04	-1.03	2.19E-03	-0.96	NA	pellino E3 ubiquitin protein ligase 1	protein coding
ENSG00000137747	TMPRSS13	11	1.26	0.03	-1.97	2.10E-05	-1.30	0.06	transmembrane protease, serine 13	protein coding
ENSG00000164236	ANKRD33B	5	1.26	0.03	-1.56	9.60E-04	-0.76	NA	ankyrin repeat domain 33B	protein coding
ENSG00000135821	GLUL	1	1.25	0.01	-0.96	0.03	-1.08	0.07	glutamate-ammonia ligase	protein coding
ENSG00000116514	RNF19B	1	1.25	1.73E-03	-0.95	0.01	-1.04	0.03	ring finger protein 19B	protein coding
ENSG00000164430	MB21D1	6	1.24	0.01	-1.53	1.31E-04	-1.37	0.02	Mab-21 domain containing 1	protein coding
ENSG00000196843	ARID5A	2	1.23	1.44E-03	-1.08	1.56E-03	-1.01	0.03	AT-rich interaction domain 5A	protein coding
ENSG00000137947	GTF2B	1	1.23	1.48E-03	-0.71	0.05	-0.84	0.08	general transcription factor IIB	protein coding
ENSG00000143384	MCL1	1	1.23	8.79E-05	-0.47	0.14	-0.69	0.08	BCL2 family apoptosis regulator	protein coding
ENSG00000262179	RP1-302G2,5	6	1.22	0.01	-1.17	3.21E-03	-0.71	NA	Homo sapiens uncharacterized LOC101929726 (LOC101929726), transcript variant 2, mRNA,	protein coding
ENSG00000235750	KIAA0040	1	1.22	1.54E-03	-1.04	2.32E-03	-1.01	0.03	KIAA0040	protein coding
ENSG00000162616	DNAJB4	1	1.22	0.03	-0.96	0.06	-1.06	0.11	DnaJ heat shock protein family (Hsp40) member B4	protein coding
ENSG00000204642	HLA-F	6	1.22	0.01	-2.08	3.25E-09	-0.77	0.14	major histocompatibility complex, class I, F	protein coding
ENSG00000174485	DENND4A	15	1.21	0.02	-1.66	8.03E-05	-1.10	0.07	DENN domain containing 4A	protein coding
ENSG00000135070	ISCA1	9	1.21	4.74E-03	-0.60	0.14	-0.88	0.09	iron-sulfur cluster assembly 1	protein coding
ENSG00000196735	HLA-DQA1	6	1.21	0.01	-1.92	1.20E-06	-0.87	0.13	major histocompatibility complex, class II, DQ alpha 1	protein coding
ENSG00000131979	GCH1	14	1.20	0.04	-1.80	1.09E-04	-1.19	0.08	GTP cyclohydrolase 1	protein coding
ENSG00000160606	TLCD1	17	1.20	0.02	-1.07	0.02	-0.85	0.15	TLC domain containing 1	protein coding
ENSG00000172171	TEFM	17	1.20	4.39E-03	-0.52	0.19	-0.65	NA	transcription elongation factor, mitochondrial	protein coding
ENSG00000038210	PI4K2B	4	1.20	0.04	-0.81	0.12	-0.79	0.22	phosphatidylinositol 4-kinase type 2 beta	protein coding
ENSG00000115604	IL18R1	2	1.20	0.01	-0.99	0.01	-0.90	0.09	interleukin 18 receptor 1	protein coding
ENSG00000166949	SMAD3	15	1.20	1.18E-03	-1.25	8.49E-05	-1.00	0.03	SMAD family member 3	protein coding
ENSG00000182809	CRIP2	14	1.20	0.01	-1.46	2.97E-04	-0.52	NA	cysteine rich protein 2	protein coding
ENSG00000149131	SERPING1	11	1.20	3.31E-03	-1.74	2.47E-07	-0.78	0.12	serpin family G member 1	protein coding
ENSG00000112759	SLC29A1	6	1.20	5.46E-04	-0.51	0.13	-0.50	0.23	solute carrier family 29 member 1 (Augustine blood group)	protein coding
ENSG00000258738	RP11-73E17,2	14	1.19	1.41E-03	-1.52	1.10E-06	-0.82	NA		antisense
ENSG00000128641	MYO1B	2	1.18	0.01	-1.50	1.07E-04	-0.90	0.10	myosin IB	protein coding
ENSG00000027075	PRKCH	14	1.18	9.89E-04	-1.31	1.90E-05	-0.85	0.06	protein kinase C eta	protein coding
ENSG00000039068	CDH1	16	1.17	2.13E-03	-0.45	0.23	-0.51	0.25	cadherin 1	protein coding
ENSG00000240065	PSMB9	6	1.17	0.02	-1.49	1.76E-04	-0.56	0.29	proteasome subunit beta 9	protein coding
ENSG00000196189	SEMA4A	1	1.17	3.41E-03	-1.68	3.77E-07	-0.90	0.07	semaphorin 4A	protein coding
ENSG00000172785	CBWD1	9	1.17	0.02	-0.57	0.22	-1.14	NA	COBW domain containing 1	protein coding
ENSG00000106803	SEC61B	9	1.16	6.82E-04	-0.52	0.12	-0.47	0.25	Sec61 translocon beta subunit	protein coding
ENSG00000213024	NUP62	19	1.15	4.51E-03	-0.97	0.01	-0.86	0.08	nucleoporin 62	protein coding
ENSG00000057704	TMCC3	12	1.14	0.03	-1.63	9.99E-05	-0.68	0.24	transmembrane and coiled-coil domain family 3	protein coding
ENSG00000122862	SRGN	10	1.14	0.01	-1.21	5.00E-04	-0.63	0.18	serglycin	protein coding
ENSG00000069399	BCL3	19	1.14	2.84E-03	-0.91	0.01	-0.96	0.04	B-cell CLL/lymphoma 3	protein coding
ENSG00000128335	APOL2	22	1.13	0.03	-1.27	4.03E-03	-0.99	0.10	apolipoprotein L2	protein coding

ENSG00000180992	MRPL14	6	1.12	4.81E-04	-0.45	0.15	-0.48	NA	mitochondrial ribosomal protein L14	protein coding
ENSG00000020633	RUNX3	1	1.12	3.41E-03	-1.07	1.26E-03	-1.02	0.03	runt related transcription factor 3	protein coding
ENSG00000099860	GADD45B	19	1.12	0.03	-0.77	0.09	-1.26	0.03	growth arrest and DNA damage inducible beta	protein coding
ENSG00000010818	HIVEP2	6	1.12	0.04	-1.40	1.74E-03	-1.03	0.10	human immunodeficiency virus type I enhancer binding protein 2	protein coding
ENSG00000119523	ALG2	9	1.12	0.01	-0.41	0.35	-0.64	0.20	ALG2, alpha-1,3/1,6-mannosyltransferase	protein coding
ENSG00000070190	DAPP1	4	1.12	2.65E-04	-0.77	0.01	-0.77	0.05	dual adaptor of phosphotyrosine and 3-phosphoinositides 1	protein coding
ENSG00000155760	FZD7	2	1.12	0.05	-1.28	4.96E-03	-0.92	NA	frizzled class receptor 7	protein coding
ENSG00000268804	LINC02132	16	1.11	0.03	-1.98	4.99E-07	-0.72	NA	long intergenic non-protein coding RNA 2132	lincRNA
ENSG00000203760	CENPW	6	1.11	0.04	-1.25	0.01	-0.16	NA	centromere protein W	protein coding
ENSG00000120526	NUDCD1	8	1.11	0.02	-0.88	0.04	-0.89	0.11	NudC domain containing 1	protein coding
ENSG00000080371	RAB21	12	1.11	1.42E-03	-0.39	0.26	-0.73	0.09	RAB21, member RAS oncogene family	protein coding
ENSG00000171552	BCL2L1	20	1.10	0.01	-1.44	3.07E-05	-0.86	0.08	BCL2 like 1	protein coding
ENSG00000152749	GPR180	13	1.10	0.04	-0.60	0.24	-0.60	0.31	G protein-coupled receptor 180	protein coding
ENSG00000155962	CLIC2	X	1.10	0.01	-1.07	1.96E-03	-0.81	NA	chloride intracellular channel 2	protein coding
ENSG00000179344	HLA-DQB1	6	1.09	3.88E-03	-0.80	0.02	-0.65	0.14	major histocompatibility complex, class II, DQ beta 1	protein coding
ENSG00000117475	BLZF1	1	1.09	0.03	-0.93	0.03	-0.87	0.12	basic leucine zipper nuclear factor 1	protein coding
ENSG00000169604	ANTXR1	2	1.09	0.05	-0.92	0.04	-0.33	NA	anthrax toxin receptor 1	protein coding
ENSG00000160293	VAV2	9	1.09	0.02	-0.70	0.11	-0.84	0.13	vav guanine nucleotide exchange factor 2	protein coding
ENSG00000196116	TDRD7	9	1.09	0.04	-1.15	0.01	-0.76	0.20	tudor domain containing 7	protein coding
ENSG00000198355	PIM3	22	1.09	4.75E-03	-0.58	0.11	-0.65	0.15	Pim-3 proto-oncogene, serine/threonine kinase	protein coding
ENSG00000025708	TYMP	22	1.09	0.03	-1.21	3.29E-03	-0.67	0.23	thymidine phosphorylase	protein coding
ENSG00000169181	GSG1L	16	1.08	0.05	-0.86	0.06	-0.29	NA	GSG1 like	protein coding
ENSG00000108639	SYNGR2	17	1.08	4.74E-05	-0.56	0.03	-0.32	0.31	synaptogyrin 2	protein coding
ENSG00000164465	DCBLD1	6	1.08	0.03	-0.97	0.02	-1.29	NA	discoidin, CUB and LCCL domain containing 1	protein coding
ENSG00000072682	P4HA2	5	1.08	0.04	-1.51	4.50E-04	-0.22	NA	prolyl 4-hydroxylase subunit alpha 2	protein coding
ENSG00000100220	RTCB	22	1.08	3.76E-04	-0.57	0.05	-0.62	0.10	RNA 2',3'-cyclic phosphate and 5'-OH ligase	protein coding
ENSG00000168672	FAM84B	8	1.07	0.03	-1.24	2.46E-03	-0.80	NA	family with sequence similarity 84 member B	protein coding
ENSG00000254087	LYN	8	1.07	4.09E-04	-0.46	0.12	-0.52	0.15	LYN proto-oncogene, Src family tyrosine kinase	protein coding
ENSG00000049449	RCN1	11	1.07	1.38E-04	-1.12	3.49E-06	-0.53	NA	reticulocalbin 1	protein coding
ENSG00000204264	PSMB8	6	1.06	0.01	-1.47	8.07E-06	-0.53	0.25	proteasome subunit beta 8	protein coding
ENSG00000105499	PLA2G4C	19	1.06	0.02	-1.73	1.19E-06	-0.84	NA	phospholipase A2 group IVC	protein coding
ENSG00000123609	NMI	2	1.05	0.05	-0.94	0.04	-0.93	0.12	N-myc and STAT interactor	protein coding
ENSG00000104299	INTS9	8	1.05	0.01	-1.42	1.28E-05	-0.86	0.07	integrator complex subunit 9	protein coding
ENSG00000204592	HLA-E	6	1.04	0.02	-0.66	0.09	-0.60	0.22	major histocompatibility complex, class I, E	protein coding
ENSG00000076641	PAG1	8	1.04	1.48E-03	-0.69	0.02	-0.73	0.08	phosphoprotein membrane anchor with glycosphingolipid microdomains 1	protein coding
ENSG00000137216	TMEM63B	6	1.04	0.02	-0.81	0.04	-0.84	0.11	transmembrane protein 63B	protein coding
ENSG00000271856	LINC01215	3	1.04	3.02E-03	-1.16	9.40E-05	-0.82	0.06	long intergenic non-protein coding RNA 1215	lincRNA
ENSG00000105939	ZC3HAV1	7	1.04	0.01	-0.26	0.52	-0.94	0.04	zinc finger CCCH-type containing, antiviral 1	protein coding
ENSG00000262874	C19orf84	19	1.03	0.03	-2.54	5.47E-11	-0.81	NA	chromosome 19 open reading frame 84	protein coding
ENSG00000110324	IL10RA	11	1.03	0.01	-0.57	0.15	-0.72	0.14	interleukin 10 receptor subunit alpha	protein coding

ENSG00000163565	IFI16	1	1.03	0.05	-0.90	0.04	-0.77	0.18	interferon gamma inducible protein 16	protein coding
ENSG00000136816	TOR1B	9	1.03	0.02	-0.58	0.16	-0.76	0.14	torsin family 1 member B	protein coding
ENSG00000179104	TMTC2	12	1.03	0.04	-1.24	2.64E-03	-0.73	NA	transmembrane and tetratricopeptide repeat containing 2	protein coding
ENSG00000026751	SLAMF7	1	1.03	0.02	-1.48	5.26E-05	-0.79	0.13	SLAM family member 7	protein coding
ENSG00000103811	CTSH	15	1.03	0.01	-1.60	1.79E-07	-0.47	0.28	cathepsin H	protein coding
ENSG00000087253	LPCAT2	16	1.02	0.02	-1.32	2.86E-04	-0.60	NA	lysophosphatidylcholine acyltransferase 2	protein coding
ENSG00000168310	IRF2	4	1.02	4.99E-04	-1.41	1.08E-08	-0.66	0.08	interferon regulatory factor 2	protein coding
ENSG00000179840	PIK3CD-AS1	1	1.02	0.02	-1.81	1.41E-07	-0.54	NA	PIK3CD antisense RNA 1	antisense
ENSG00000154642	C21orf91	21	1.02	0.01	-0.67	0.06	-0.64	0.16	chromosome 21 open reading frame 91	protein coding
ENSG00000197122	SRC	20	1.02	4.87E-03	-0.87	0.01	-0.74	0.09	SRC proto-oncogene, non-receptor tyrosine kinase	protein coding
ENSG00000112773	FAM46A	6	1.01	2.18E-03	-0.77	0.01	-0.65	0.10	family with sequence similarity 46 member A	protein coding
ENSG00000154640	BTG3	21	1.01	0.02	-0.58	0.13	-0.92	NA	BTG anti-proliferation factor 3	protein coding
ENSG00000125898	FAM110A	20	1.01	1.71E-04	-0.86	2.75E-04	-0.54	NA	family with sequence similarity 110 member A	protein coding
ENSG00000176102	CSTF3	11	1.01	0.01	-0.03	0.96	-0.76	0.10	cleavage stimulation factor subunit 3	protein coding
ENSG00000151651	ADAM8	10	1.00	0.01	-1.51	6.20E-07	-0.75	NA	ADAM metallopeptidase domain 8	protein coding
ENSG00000133639	BTG1	12	1.00	0.01	-0.29	0.45	-0.74	0.10	BTG anti-proliferation factor 1	protein coding
ENSG00000137449	CPEB2	4	1.00	0.04	-0.87	0.04	-0.52	NA	cytoplasmic polyadenylation element binding protein 2	protein coding
ENSG00000064651	SLC12A2	5	0.99	0.04	-1.51	7.49E-05	-0.89	0.10	solute carrier family 12 member 2	protein coding
ENSG00000154721	JAM2	21	0.99	0.01	-1.39	2.79E-05	-0.77	NA	junctional adhesion molecule 2	protein coding
ENSG00000145365	TIFA	4	0.99	8.79E-05	-0.47	0.05	-0.60	0.07	TRAF interacting protein with forkhead associated domain	protein coding
ENSG00000140548	ZNF710	15	0.99	3.97E-03	-1.29	5.98E-06	-0.77	0.07	zinc finger protein 710	protein coding
ENSG00000239713	APOBEC3G	22	0.98	3.12E-04	-1.30	1.29E-08	-0.73	0.03	apolipoprotein B mRNA editing enzyme catalytic subunit 3G	protein coding
ENSG00000067221	STOML1	15	0.98	0.01	-0.46	0.17	-0.58	NA	stomatin like 1	protein coding
ENSG00000172345	STARD5	15	0.98	0.02	-0.69	0.07	-1.38	NA	StAR related lipid transfer domain containing 5	protein coding
ENSG00000106560	GIMAP2	7	0.98	4.81E-04	-0.66	0.01	-0.52	NA	GTPase, IMAP family member 2	protein coding
ENSG00000171631	P2RY6	11	0.97	0.02	-1.15	6.00E-04	-0.71	0.13	pyrimidinergic receptor P2Y6	protein coding
ENSG00000069974	RAB27A	15	0.96	0.03	-1.28	3.47E-04	-0.65	NA	RAB27A, member RAS oncogene family	protein coding
ENSG00000140105	WARS	14	0.96	0.01	-0.22	0.57	-0.60	0.14	tryptophanyl-tRNA synthetase	protein coding
ENSG00000124508	BTN2A2	6	0.96	0.01	-1.42	9.77E-07	-0.77	0.07	butyrophilin subfamily 2 member A2	protein coding
ENSG00000102218	RP2	X	0.95	0.01	-0.46	0.20	-0.53	0.22	retinitis pigmentosa 2 (X-linked recessive)	protein coding
ENSG00000108829	LRRC59	17	0.95	0.02	-0.33	0.41	-0.53	0.24	leucine rich repeat containing 59	protein coding
ENSG00000086061	DNAJA1	9	0.95	0.01	-0.93	2.93E-03	-0.72	0.10	DnaJ heat shock protein family (Hsp40) member A1	protein coding
ENSG00000072415	MPP5	14	0.95	3.88E-03	-0.24	0.48	-0.64	NA	membrane palmitoylated protein 5	protein coding
ENSG00000138670	RASGEF1B	4	0.95	0.03	-0.76	0.04	-0.79	0.11	RasGEF domain family member 1B	protein coding
ENSG00000206503	HLA-A	6	0.95	2.76E-03	-1.08	6.97E-05	-0.38	0.30	major histocompatibility complex, class I, A	protein coding
ENSG00000117143	UAP1	1	0.95	0.05	-0.59	0.18	-0.93	0.10	UDP-N-acetylglucosamine pyrophosphorylase 1	protein coding
ENSG00000178078	STAP2	19	0.94	0.03	-1.18	6.50E-04	-0.34	NA	signal transducing adaptor family member 2	protein coding
ENSG00000198369	SPRED2	2	0.94	0.03	-1.09	2.00E-03	-0.71	0.14	sprouty related EVH1 domain containing 2	protein coding
ENSG00000106266	SNX8	7	0.94	1.18E-03	-0.98	7.49E-05	-0.29	NA	sorting nexin 8	protein coding
ENSG00000139180	NDUFA9	12	0.94	0.02	-0.45	0.23	-0.67	NA	NADH:ubiquinone oxidoreductase subunit A9	protein coding
ENSG00000171161	ZNF672	1	0.93	4.81E-04	-1.03	8.51E-06	-0.53	0.11	zinc finger protein 672	protein coding

ENSG00000109971	HSPA8	11	0.93	0.03	-0.98	0.01	-0.54	0.25	heat shock protein family A (Hsp70) member 8	protein coding
ENSG00000096384	HSP90AB1	6	0.93	0.02	-0.59	0.09	-0.58	0.19	heat shock protein 90 alpha family class B member 1	protein coding
ENSG00000065054	SLC9A3R2	16	0.93	6.74E-04	-0.59	0.02	-0.55	0.10	SLC9A3 regulator 2	protein coding
ENSG00000134802	SLC43A3	11	0.93	0.01	-1.15	1.52E-04	-0.55	0.19	solute carrier family 43 member 3	protein coding
ENSG00000118503	TNFAIP3	6	0.92	3.10E-03	-0.85	1.69E-03	-0.78	0.04	TNF alpha induced protein 3	protein coding
ENSG00000102221	JADE3	X	0.92	4.87E-03	-0.59	0.05	-0.50	0.19	jade family PHD finger 3	protein coding
ENSG00000158470	B4GALT5	20	0.92	2.36E-03	-0.46	0.11	-0.69	0.07	beta-1,4-galactosyltransferase 5	protein coding
ENSG00000160710	ADAR	1	0.91	0.04	-0.42	0.34	-0.55	0.26	adenosine deaminase, RNA specific	protein coding
ENSG00000177426	TGIF1	18	0.91	0.05	-1.15	2.38E-03	-0.55	NA	TGFB induced factor homeobox 1	protein coding
ENSG00000213516	RBMXL1	1	0.91	0.02	-0.57	0.11	-0.39	NA	RNA binding motif protein, X-linked like 1	protein coding
ENSG00000085063	CD59	11	0.90	1.32E-03	-0.85	4.69E-04	-0.39	0.23	CD59 molecule	protein coding
ENSG00000153029	MR1	1	0.89	0.05	-0.38	0.39	-0.66	0.19	major histocompatibility complex, class I-related	protein coding
ENSG00000254685	FPGT	1	0.89	0.01	-0.75	0.01	-0.33	NA	fucose-1-phosphate guanylyltransferase	protein coding
ENSG00000188042	ARL4C	2	0.89	6.82E-04	-0.83	3.42E-04	-0.68	0.04	ADP ribosylation factor like GTPase 4C	protein coding
ENSG00000136732	GYPC	2	0.89	0.01	-1.07	3.81E-04	-0.31	NA	glycophorin C (Gerbich blood group)	protein coding
ENSG00000115956	PLEK	2	0.89	2.27E-06	-0.47	0.01	-0.35	0.14	pleckstrin	protein coding
ENSG00000185262	UBALD2	17	0.88	0.01	-0.65	0.04	-0.44	0.27	UBA like domain containing 2	protein coding
ENSG00000161791	FMNL3	12	0.87	4.35E-03	-0.38	0.20	-0.64	0.09	formin like 3	protein coding
ENSG00000176087	SLC35A4	5	0.87	0.01	-0.80	3.52E-03	-0.42	0.25	solute carrier family 35 member A4	protein coding
ENSG00000106078	COBL	7	0.87	0.05	-1.46	2.86E-05	-0.79	0.12	cordon-bleu WH2 repeat protein	protein coding
ENSG00000100911	PSME2	14	0.87	0.02	-0.86	0.01	-0.31	0.46	proteasome activator subunit 2	protein coding
ENSG00000173926	MARCH3	5	0.86	0.05	-1.27	2.85E-04	-1.20	NA	membrane associated ring-CH-type finger 3	protein coding
ENSG00000135211	TMEM60	7	0.86	2.68E-03	-0.16	0.60	-0.21	NA	transmembrane protein 60	protein coding
ENSG00000197872	FAM49A	2	0.86	2.32E-03	-0.14	0.67	-0.61	0.08	family with sequence similarity 49 member A	protein coding
ENSG00000173917	HOXB2	17	0.86	1.32E-03	-1.21	4.28E-08	-1.14	NA	homeobox B2	protein coding
ENSG00000144655	CSRNP1	3	0.86	0.01	-0.27	0.44	-0.61	0.13	cysteine and serine rich nuclear protein 1	protein coding
ENSG00000145901	TNIP1	5	0.85	2.63E-04	-0.13	0.63	-0.43	0.14	TNFAIP3 interacting protein 1	protein coding
ENSG00000116670	MAD2L2	1	0.85	1.46E-05	-0.72	4.12E-05	-0.38	0.12	MAD2 mitotic arrest deficient-like 2 (yeast)	protein coding
ENSG00000173264	GPR137	11	0.85	9.80E-04	-0.84	1.49E-04	-0.16	0.61	G protein-coupled receptor 137	protein coding
ENSG00000196387	ZNF140	12	0.84	0.01	-0.35	0.25	-0.51	NA	zinc finger protein 140	protein coding
ENSG00000174125	TLR1	4	0.84	1.36E-04	-0.68	5.09E-04	-0.43	NA	toll like receptor 1	protein coding
ENSG00000167136	ENDOG	9	0.84	0.03	-1.29	4.91E-05	-0.54	0.22	endonuclease G	protein coding
ENSG00000135363	LMO2	11	0.84	0.01	-1.93	3.01E-13	-0.62	NA	LIM domain only 2	protein coding
ENSG00000173517	PEAK1	15	0.84	0.05	-1.44	1.85E-05	-0.89	0.07	pseudopodium enriched atypical kinase 1	protein coding
ENSG00000234745	HLA-B	6	0.83	2.81E-03	-0.40	0.14	-0.22	0.49	major histocompatibility complex, class I, B	protein coding
ENSG00000167208	SNX20	16	0.83	1.44E-04	-0.94	5.19E-07	-0.49	0.08	sorting nexin 20	protein coding
ENSG00000158457	TSPAN33	7	0.83	0.01	-0.31	0.36	-0.34	0.36	tetraspanin 33	protein coding
ENSG00000100097	LGALS1	22	0.82	0.01	-1.34	5.13E-07	-0.14	0.73	galectin 1	protein coding
ENSG00000106591	MRPL32	7	0.82	0.01	-0.35	0.27	-0.65	NA	mitochondrial ribosomal protein L32	protein coding
ENSG00000100084	HIRA	22	0.82	0.05	-0.59	0.11	-0.66	NA	histone cell cycle regulator	protein coding
ENSG00000180817	PPA1	10	0.82	0.05	-0.56	0.13	-0.47	0.30	pyrophosphatase (inorganic) 1	protein coding
ENSG00000173171	MTX1	1	0.81	0.03	-0.02	0.97	-0.15	NA	metaxin 1	protein coding

ENSG00000120675	DNAJC15	13	0.81	0.02	-0.25	0.50	-0.33	NA	DnaJ heat shock protein family (Hsp40) member C15	protein coding
ENSG00000087086	FTL	19	0.81	0.01	-0.97	2.90E-04	-0.45	0.22	ferritin light chain	protein coding
ENSG00000128973	CLN6	15	0.81	0.01	-1.09	4.06E-05	-0.44	NA	ceroid-lipofuscinosis, neuronal 6, late infantile, variant	protein coding
ENSG00000143156	NME7	1	0.81	0.02	-0.36	0.26	-0.25	NA	NME/NM23 family member 7	protein coding
ENSG00000115919	KYNU	2	0.81	0.02	-0.75	0.01	-0.42	0.28	kynureninase	protein coding
ENSG00000227507	LTB	6	0.81	2.91E-05	-1.28	3.21E-15	-0.25	0.30	lymphotoxin beta	protein coding
ENSG00000160602	NEK8	17	0.81	0.05	-1.06	1.43E-03	-0.77	0.10	NIMA related kinase 8	protein coding
ENSG00000100403	ZC3H7B	22	0.80	0.02	-0.63	0.03	-0.76	0.06	zinc finger CCCH-type containing 7B	protein coding
ENSG00000259431	THTPA	14	0.80	0.03	-0.83	0.01	-0.54	NA	thiamine triphosphatase	protein coding
ENSG00000166710	B2M	15	0.80	0.01	-0.42	0.17	-0.27	0.46	beta-2-microglobulin	protein coding
ENSG00000092203	TOX4	14	0.80	0.03	-0.75	0.02	-0.60	0.14	TOX high mobility group box family member 4	protein coding
ENSG00000062194	GPBP1	5	0.79	0.04	-0.59	0.08	-0.77	0.08	GC-rich promoter binding protein 1	protein coding
ENSG00000115271	GCA	2	0.79	0.01	-1.02	6.97E-05	-0.53	NA	grancalcin	protein coding
ENSG00000173163	COMMD1	2	0.79	0.01	-0.59	0.02	-0.30	NA	copper metabolism domain containing 1	protein coding
ENSG00000085117	CD82	11	0.79	0.01	-0.39	0.17	-0.25	0.45	CD82 molecule	protein coding
ENSG00000124789	NUP153	6	0.79	0.01	-0.76	4.40E-03	-0.58	0.11	nucleoporin 153	protein coding
ENSG00000213719	CLIC1	6	0.79	0.04	-0.56	0.10	-0.26	0.53	chloride intracellular channel 1	protein coding
ENSG00000120533	ENY2	8	0.79	0.01	-0.30	0.33	-0.54	0.13	ENY2, transcription and export complex 2 subunit	protein coding
ENSG00000198736	MSRB1	16	0.79	0.01	-0.29	0.32	-0.42	0.21	methionine sulfoxide reductase B1	protein coding
ENSG00000015475	BID	22	0.78	0.01	-0.64	0.01	-0.41	0.22	BH3 interacting domain death agonist	protein coding
ENSG00000196664	TLR7	X	0.78	0.03	-0.30	0.42	-0.41	0.30	toll like receptor 7	protein coding
ENSG00000168610	STAT3	17	0.78	0.02	-0.88	1.32E-03	-0.62	0.11	signal transducer and activator of transcription 3	protein coding
ENSG00000151748	SAV1	14	0.78	1.07E-03	-0.50	0.02	-0.47	0.11	salvador family WW domain containing protein 1	protein coding
ENSG00000172936	MYD88	3	0.78	0.05	-0.54	0.13	-0.41	0.33	myeloid differentiation primary response 88	protein coding
ENSG00000083799	CYLD	16	0.78	2.68E-03	-0.58	0.01	-0.59	0.07	CYLD lysine 63 deubiquitinase	protein coding
ENSG00000263563	UBBP4	17	0.78	0.01	-0.48	0.08	-0.33	NA	ubiquitin B pseudogene 4	transcribed unprocessed pseudogene
ENSG00000104825	NFKBIB	19	0.78	0.02	-0.51	0.09	-0.60	0.12	NFKB inhibitor beta	protein coding
ENSG00000276045	ORAI1	12	0.77	3.34E-03	-0.43	0.08	-0.07	0.84	ORAI calcium release-activated calcium modulator 1	protein coding
ENSG00000132507	EIF5A	17	0.77	2.76E-03	-0.35	0.16	-0.24	0.42	eukaryotic translation initiation factor 5A	protein coding
ENSG00000065427	KARS	16	0.77	0.02	-0.50	0.10	-0.39	0.30	lysyl-tRNA synthetase	protein coding
ENSG00000110756	HPS5	11	0.77	0.01	-0.54	0.04	-0.57	0.10	HPS5, biogenesis of lysosomal organelles complex 2 subunit 2	protein coding
ENSG00000163389	POGLUT1	3	0.77	0.05	-1.14	2.58E-04	-0.73	0.10	protein O-glucosyltransferase 1	protein coding
ENSG00000178741	COX5A	15	0.77	0.04	-0.64	0.05	-0.31	0.45	cytochrome c oxidase subunit 5A	protein coding
ENSG00000003400	CASP10	2	0.76	0.03	-0.99	7.55E-04	-0.67	0.10	caspase 10	protein coding
ENSG00000145088	EAF2	3	0.76	0.03	-2.27	4.52E-16	-0.20	NA	ELL associated factor 2	protein coding
ENSG00000106153	CHCHD2	7	0.76	0.02	-0.16	0.65	-0.13	0.73	coiled-coil-helix-coiled-coil-helix domain containing 2	protein coding
ENSG00000253276	CCDC71L	7	0.75	5.63E-05	-0.78	1.11E-06	-0.59	0.01	coiled-coil domain containing 71-like	protein coding
ENSG00000138639	ARHGAP24	4	0.75	0.01	-1.19	7.47E-07	-0.36	NA	Rho GTPase activating protein 24	protein coding
ENSG00000127666	TICAM1	19	0.75	0.01	-0.33	0.22	-0.56	0.10	toll like receptor adaptor molecule 1	protein coding

ENSG00000067182	TNFRSF1A	12	0.75	0.01	-0.32	0.24	-0.43	0.19	TNF receptor superfamily member 1A	protein coding
ENSG00000103018	CYB5B	16	0.75	3.98E-03	-0.02	0.96	-0.20	0.50	cytochrome b5 type B	protein coding
ENSG00000168389	MFSD2A	1	0.74	0.03	-0.50	0.10	-0.48	0.20	major facilitator superfamily domain containing 2A	protein coding
ENSG00000105518	TMEM205	19	0.74	0.01	-0.52	0.05	-0.30	NA	transmembrane protein 205	protein coding
ENSG00000138785	INTS12	4	0.74	0.05	-0.64	0.05	-0.71	0.10	integrator complex subunit 12	protein coding
ENSG00000092820	EZR	6	0.74	0.03	-1.05	1.37E-04	-0.53	0.16	ezrin	protein coding
ENSG00000132963	POMP	13	0.74	0.03	-0.23	0.53	-0.40	0.29	proteasome maturation protein	protein coding
ENSG00000031081	ARHGAP31	3	0.74	1.55E-03	-0.04	0.89	-0.38	0.17	Rho GTPase activating protein 31	protein coding
ENSG00000176531	PHLDB3	19	0.73	0.01	-0.74	1.53E-03	-0.42	NA	pleckstrin homology like domain family B member 3	protein coding
ENSG00000162144	CYB561A3	11	0.73	0.01	-0.28	0.29	-0.06	0.86	cytochrome b561 family member A3	protein coding
ENSG00000172115	CYCS	7	0.73	0.03	-0.32	0.34	-0.39	0.29	cytochrome c, somatic	protein coding
ENSG00000129255	MPDU1	17	0.73	2.17E-03	-0.64	2.02E-03	-0.29	0.28	mannose-P-dolichol utilization defect 1	protein coding
ENSG00000181026	AEN	15	0.73	3.61E-03	0.10	0.72	-0.31	0.28	apoptosis enhancing nuclease	protein coding
ENSG00000104325	DECR1	8	0.73	0.02	-0.69	0.01	-0.47	0.19	2,4-dienoyl-CoA reductase 1	protein coding
ENSG00000183617	MRPL54	19	0.73	0.02	-0.14	0.69	-0.09	NA	mitochondrial ribosomal protein L54	protein coding
ENSG00000173801	JUP	17	0.72	0.01	-1.46	1.28E-10	-0.55	0.11	junction plakoglobin	protein coding
ENSG00000126457	PRMT1	19	0.72	5.67E-04	-0.51	0.01	-0.19	0.42	protein arginine methyltransferase 1	protein coding
ENSG00000131174	COX7B	X	0.72	0.01	-0.10	0.75	-0.28	0.38	cytochrome c oxidase subunit 7B	protein coding
ENSG00000150459	SAP18	13	0.71	0.03	-0.03	0.94	-0.43	0.23	Sin3A associated protein 18	protein coding
ENSG00000102393	GLA	X	0.71	0.02	-0.50	0.07	-0.20	0.57	galactosidase alpha	protein coding
ENSG00000100226	GTPBP1	22	0.71	0.05	-0.80	0.01	-0.55	0.17	GTP binding protein 1	protein coding
ENSG00000123595	RAB9A	X	0.71	4.75E-03	-0.36	0.13	-0.37	0.19	RAB9A, member RAS oncogene family	protein coding
ENSG00000164163	ABCE1	4	0.71	0.01	-0.61	0.01	-0.40	0.22	ATP binding cassette subfamily E member 1	protein coding
ENSG00000136560	TANK	2	0.70	0.03	-0.60	0.03	-0.64	0.08	TRAF family member associated NFKB activator	protein coding
ENSG00000083635	NUFIP1	13	0.70	0.01	-0.02	0.95	-0.38	NA	NUFIP1, FMR1 interacting protein 1	protein coding
ENSG00000100567	PSMA3	14	0.70	0.03	-0.59	0.03	-0.42	0.23	proteasome subunit alpha 3	protein coding
ENSG00000169504	CLIC4	1	0.70	4.64E-03	-0.10	0.74	-0.19	0.50	chloride intracellular channel 4	protein coding
ENSG00000156515	HK1	10	0.70	0.05	-0.50	0.10	-0.44	0.25	hexokinase 1	protein coding
ENSG00000126945	HNRNPH2	X	0.69	0.02	-1.21	3.42E-07	-0.51	0.14	heterogeneous nuclear ribonucleoprotein H2	protein coding
ENSG00000162734	PEA15	1	0.69	3.11E-03	-0.30	0.19	-0.33	0.22	phosphoprotein enriched in astrocytes 15	protein coding
ENSG00000129084	PSMA1	11	0.69	0.01	-0.34	0.16	-0.24	NA	proteasome subunit alpha 1	protein coding
ENSG00000109606	DHX15	4	0.68	0.02	-0.44	0.10	-0.51	0.13	DEAH-box helicase 15	protein coding
ENSG00000100804	PSMB5	14	0.68	0.04	0.28	0.39	0.04	0.92	proteasome subunit beta 5	protein coding
ENSG00000018699	TTC27	2	0.68	0.02	-0.75	1.44E-03	-0.48	NA	tetratricopeptide repeat domain 27	protein coding
ENSG00000147168	IL2RG	X	0.68	4.81E-04	-1.17	2.54E-13	-0.36	0.14	interleukin 2 receptor subunit gamma	protein coding
ENSG00000013364	MVP	16	0.68	0.04	-0.46	0.11	-0.19	0.59	major vault protein	protein coding
ENSG00000182481	KPNA2	17	0.67	0.02	-0.08	0.81	-0.27	0.40	karyopherin subunit alpha 2	protein coding
ENSG00000137522	RNF121	11	0.67	0.02	-0.88	2.03E-04	-0.45	0.17	ring finger protein 121	protein coding
ENSG00000149547	EI24	11	0.67	0.01	-0.21	0.45	-0.37	0.23	EI24, autophagy associated transmembrane protein	protein coding
ENSG00000100749	VRK1	14	0.67	0.02	-0.44	0.09	-0.18	NA	vaccinia related kinase 1	protein coding
ENSG00000185291	IL3RA	X	0.67	3.91E-03	-0.93	1.09E-06	-0.27	0.30	interleukin 3 receptor subunit alpha	protein coding
ENSG00000139505	MTMR6	13	0.67	0.02	-0.54	0.03	-0.28	0.39	myotubularin related protein 6	protein coding

ENSG00000133065	SLC41A1	1	0.67	0.04	-1.20	3.61E-06	-0.53	NA	solute carrier family 41 member 1	protein coding
ENSG00000051108	HERPUD1	16	0.67	0.01	0.07	0.81	-0.27	0.34	homocysteine inducible ER protein with ubiquitin like domain 1	protein coding
ENSG00000164305	CASP3	4	0.67	0.01	-0.33	0.16	-0.46	0.12	caspase 3	protein coding
ENSG00000138279	ANXA7	10	0.66	2.47E-04	-0.65	4.12E-05	-0.34	0.12	annexin A7	protein coding
ENSG00000231925	TAPBP	6	0.66	2.17E-03	-0.65	4.71E-04	-0.34	0.18	TAP binding protein	protein coding
ENSG00000173812	EIF1	17	0.66	0.01	-0.18	0.51	-0.36	0.22	eukaryotic translation initiation factor 1	protein coding
ENSG00000143106	PSMA5	1	0.66	0.02	-0.43	0.10	-0.29	0.36	proteasome subunit alpha 5	protein coding
ENSG00000143514	TP53BP2	1	0.66	0.02	-0.60	0.01	-0.53	0.10	tumor protein p53 binding protein 2	protein coding
ENSG00000116473	RAP1A	1	0.66	4.81E-04	-0.45	0.01	-0.41	0.08	RAP1A, member of RAS oncogene family	protein coding
ENSG00000196126	HLA-DRB1	6	0.65	0.05	-0.57	0.04	-0.34	0.33	major histocompatibility complex, class II, DR beta 1	protein coding
ENSG00000040531	CTNS	17	0.65	0.04	-0.33	0.29	-0.50	0.17	cystinosin, lysosomal cystine transporter	protein coding
ENSG00000099875	MKNK2	19	0.65	0.03	-0.55	0.04	-0.44	0.20	MAP kinase interacting serine/threonine kinase 2	protein coding
ENSG00000125651	GTF2F1	19	0.65	3.92E-03	-0.17	0.50	-0.31	0.24	general transcription factor IIF subunit 1	protein coding
ENSG00000145780	FEM1C	5	0.65	0.03	-0.37	0.18	-0.46	NA	fem-1 homolog C	protein coding
ENSG00000043143	JADE2	5	0.65	0.02	-1.44	8.54E-11	-0.50	0.13	jade family PHD finger 2	protein coding
ENSG00000121774	KHDRBS1	1	0.65	0.02	-1.58	1.63E-13	-0.50	0.12	KH RNA binding domain containing, signal transduction associated 1	protein coding
ENSG00000143368	SF3B4	1	0.65	9.18E-04	-0.14	0.50	-0.24	0.29	splicing factor 3b subunit 4	protein coding
ENSG00000136490	LIMD2	17	0.65	4.34E-04	-0.87	1.34E-08	-0.40	0.08	LIM domain containing 2	protein coding
ENSG00000143390	RFX5	1	0.65	0.05	-1.16	6.15E-06	-0.67	0.08	regulatory factor X5	protein coding
ENSG00000108622	ICAM2	17	0.64	0.01	-0.50	0.03	-0.30	0.30	intercellular adhesion molecule 2	protein coding
ENSG00000205208	C4orf46	4	0.64	0.05	-1.31	2.89E-07	-0.34	NA	chromosome 4 open reading frame 46	protein coding
ENSG00000139496	NUP58	13	0.64	0.02	-0.43	0.09	-0.46	0.15	nucleoporin 58	protein coding
ENSG00000109332	UBE2D3	4	0.64	0.03	-0.24	0.42	-0.36	0.27	ubiquitin conjugating enzyme E2 D3	protein coding
ENSG00000112578	BYSL	6	0.64	0.01	0.50	0.02	-0.27	NA	bystin like	protein coding
ENSG00000136908	DPM2	9	0.63	0.01	-0.31	0.14	-0.28	NA	dolichyl-phosphate mannosyltransferase subunit 2, regulatory	protein coding
ENSG00000277791	PSMB3	17	0.63	0.02	-0.02	0.95	-0.13	0.68	proteasome subunit beta 3	protein coding
ENSG00000146834	MEPCE	7	0.63	3.41E-03	-0.89	5.36E-07	-0.35	0.17	methylphosphate capping enzyme	protein coding
ENSG00000181481	RNF135	17	0.63	0.02	-0.11	0.69	-0.37	NA	ring finger protein 135	protein coding
ENSG00000120725	SIL1	5	0.63	0.02	-0.65	3.08E-03	-0.36	NA	SIL1 nucleotide exchange factor	protein coding
ENSG00000139192	TAPBPL	12	0.63	0.05	-1.33	4.80E-08	-0.47	NA	TAP binding protein like	protein coding
ENSG00000154978	VOPP1	7	0.63	3.16E-03	-0.39	0.05	-0.06	0.83	vesicular, overexpressed in cancer, prosurvival protein 1	protein coding
ENSG00000111912	NCOA7	6	0.63	0.03	-0.67	0.01	-0.68	0.05	nuclear receptor coactivator 7	protein coding
ENSG00000133265	HSPBP1	19	0.62	0.03	-0.16	0.62	-0.14	NA	HSPA (Hsp70) binding protein 1	protein coding
ENSG00000122884	P4HA1	10	0.62	0.03	-0.32	0.24	-0.39	0.23	prolyl 4-hydroxylase subunit alpha 1	protein coding
ENSG00000160563	MED27	9	0.62	0.01	-0.13	0.59	-0.14	NA	mediator complex subunit 27	protein coding
ENSG00000104812	GYS1	19	0.62	0.02	-1.10	3.14E-07	-0.45	0.14	glycogen synthase 1	protein coding
ENSG00000111669	TPI1	12	0.62	0.03	-0.60	0.01	-0.14	0.65	triosephosphate isomerase 1	protein coding
ENSG00000002919	SNX11	17	0.62	0.03	-0.12	0.72	-0.31	NA	sorting nexin 11	protein coding
ENSG00000163659	TIPARP	3	0.62	0.02	-0.64	3.42E-03	-0.54	0.08	TCDD inducible poly(ADP-ribose) polymerase	protein coding
ENSG00000170315	UBB	17	0.62	0.03	-0.26	0.36	-0.08	0.82	ubiquitin B	protein coding

ENSG00000103274	NUBP1	16	0.61	7.70E-04	-0.17	0.37	-0.38	0.09	nucleotide binding protein 1	protein coding
ENSG00000165732	DDX21	10	0.61	0.04	-0.24	0.43	-0.51	0.14	DExD-box helicase 21	protein coding
ENSG00000159335	PTMS	12	0.61	0.03	-0.99	7.57E-06	-0.36	0.24	parathymosin	protein coding
ENSG00000140157	NIPA2	15	0.61	0.03	-0.14	0.65	-0.23	0.47	non imprinted in Prader-Willi/Angelman syndrome 2	protein coding
ENSG00000204574	ABCF1	6	0.61	2.04E-03	-0.20	0.33	-0.32	0.17	ATP binding cassette subfamily F member 1	protein coding
ENSG00000143575	HAX1	1	0.61	0.05	-0.10	0.78	-0.15	0.67	HCLS1 associated protein X-1	protein coding
ENSG00000149136	SSRP1	11	0.61	0.01	-0.20	0.38	-0.37	0.15	structure specific recognition protein 1	protein coding
ENSG00000171135	JAGN1	3	0.61	0.01	-0.10	0.70	-0.08	0.78	jagunal homolog 1	protein coding
ENSG00000165916	PSMC3	11	0.60	0.01	-0.41	0.03	-0.26	0.29	proteasome 26S subunit, ATPase 3	protein coding
ENSG00000183726	TMEM50A	1	0.60	0.03	0.08	0.80	-0.19	0.52	transmembrane protein 50A	protein coding
ENSG00000157191	NECAP2	1	0.60	1.44E-03	-0.26	0.15	-0.12	0.60	NECAP endocytosis associated 2	protein coding
ENSG00000100401	RANGAP1	22	0.60	0.03	-0.27	0.31	-0.40	0.18	Ran GTPase activating protein 1	protein coding
ENSG00000272886	DCP1A	3	0.60	0.05	-0.13	0.70	-0.43	0.21	decapping mRNA 1A	protein coding
ENSG00000117450	PRDX1	1	0.60	0.04	-0.49	0.05	-0.19	0.55	peroxiredoxin 1	protein coding
ENSG00000163754	GYG1	3	0.60	0.04	-0.38	0.15	-0.21	0.50	glycogenin 1	protein coding
ENSG00000115165	CYTIP	2	0.60	0.04	-0.36	0.19	-0.39	0.24	cytohesin 1 interacting protein	protein coding
ENSG00000163154	TNFAIP8L2	1	0.60	0.02	-0.68	9.11E-04	-0.05	NA	TNF alpha induced protein 8 like 2	protein coding
ENSG00000197629	MPEG1	11	0.60	4.51E-03	-1.50	3.82E-20	-0.34	0.17	macrophage expressed 1	protein coding
ENSG00000125037	EMC3	3	0.60	0.03	0.17	0.57	-0.17	0.59	ER membrane protein complex subunit 3	protein coding
ENSG00000213523	SRA1	5	0.60	0.01	-0.11	0.67	-0.10	NA	steroid receptor RNA activator 1	protein coding
ENSG00000134001	EIF2S1	14	0.60	0.03	0.01	0.97	-0.43	0.16	eukaryotic translation initiation factor 2 subunit alpha	protein coding
ENSG00000125835	SNRPB	20	0.59	0.03	0.16	0.56	-0.03	0.92	small nuclear ribonucleoprotein polypeptides B and B1	protein coding
ENSG00000159377	PSMB4	1	0.59	0.02	-0.04	0.89	-0.09	0.76	proteasome subunit beta 4	protein coding
ENSG00000142227	EMP3	19	0.59	0.05	-0.84	5.34E-04	-0.19	0.58	epithelial membrane protein 3	protein coding
ENSG00000111224	PARP11	12	0.59	0.04	-0.72	2.38E-03	-0.36	0.25	poly(ADP-ribose) polymerase family member 11	protein coding
ENSG00000105447	GRWD1	19	0.59	0.01	-0.03	0.93	-0.16	0.57	glutamate rich WD repeat containing 1	protein coding
ENSG00000055070	SZRD1	1	0.59	0.03	-0.03	0.93	-0.29	0.34	SUZ RNA binding domain containing 1	protein coding
ENSG00000163131	CTSS	1	0.59	0.02	-0.77	1.37E-04	-0.21	0.43	cathepsin S	protein coding
ENSG00000076604	TRAF4	17	0.59	0.02	-0.13	0.66	-0.52	0.08	TNF receptor associated factor 4	protein coding
ENSG00000153391	INO80C	18	0.59	0.04	-0.38	0.13	-0.20	NA	INO80 complex subunit C	protein coding
ENSG00000074266	EED	11	0.59	0.04	-1.33	1.97E-09	-0.49	NA	embryonic ectoderm development	protein coding
ENSG00000162139	NEU3	11	0.58	0.04	-0.43	0.09	-0.63	NA	neuraminidase 3	protein coding
ENSG00000131475	VPS25	17	0.58	0.01	-0.13	0.61	-0.11	NA	vacuolar protein sorting 25 homolog	protein coding
ENSG00000164442	CITED2	6	0.58	0.04	-0.48	0.05	-0.31	0.31	Cbp/p300 interacting transactivator with Glu/Asp rich carboxy-terminal domain 2	protein coding
ENSG00000174021	GNG5	1	0.58	0.05	-0.24	0.41	-0.34	0.29	G protein subunit gamma 5	protein coding
ENSG00000158042	MRPL17	11	0.58	0.03	0.12	0.67	-0.09	0.78	mitochondrial ribosomal protein L17	protein coding
ENSG00000147684	NDUFB9	8	0.58	0.03	0.05	0.89	0.00	NA	NADH:ubiquinone oxidoreductase subunit B9	protein coding
ENSG00000134250	NOTCH2	1	0.58	0.01	-0.60	2.75E-03	-0.28	0.29	notch 2	protein coding
ENSG00000232119	MCTS1	X	0.58	0.04	-0.41	0.09	-0.02	NA	MCTS1, re-initiation and release factor	protein coding
ENSG00000107263	RAPGEF1	9	0.58	0.03	-0.77	3.47E-04	-0.49	0.11	Rap guanine nucleotide exchange factor 1	protein coding

ENSG00000154582	ELOC	8	0.58	0.03	-0.17	0.55	-0.52	NA	elongin C	protein coding
ENSG00000143553	SNAPIN	1	0.58	0.05	-0.01	0.97	-0.26	NA	SNAP associated protein	protein coding
ENSG00000168701	TMEM208	16	0.57	0.04	0.20	0.48	-0.08	NA	transmembrane protein 208	protein coding
ENSG00000155115	GTF3C6	6	0.57	0.02	-0.11	0.68	-0.03	NA	general transcription factor IIIC subunit 6	protein coding
ENSG00000138758	11-sep	4	0.57	0.02	-0.22	0.35	-0.24	0.36	septin 11	protein coding
ENSG00000084623	EIF3I	1	0.57	0.03	0.00	0.99	-0.03	0.92	eukaryotic translation initiation factor 3 subunit I	protein coding
ENSG00000047249	ATP6V1H	8	0.57	0.02	-0.21	0.37	-0.26	0.33	ATPase H+ transporting V1 subunit H	protein coding
ENSG00000169567	HINT1	5	0.57	0.03	0.06	0.84	-0.03	0.93	histidine triad nucleotide binding protein 1	protein coding
ENSG00000134352	IL6ST	5	0.57	0.05	0.02	0.96	-0.32	0.30	interleukin 6 signal transducer	protein coding
ENSG00000231389	HLA-DPA1	6	0.56	0.02	-0.43	0.04	-0.12	0.66	major histocompatibility complex, class II, DP alpha 1	protein coding
ENSG00000014138	POLA2	11	0.56	0.03	-1.31	1.65E-11	-0.51	NA	DNA polymerase alpha 2, accessory subunit	protein coding
ENSG00000095380	NANS	9	0.56	0.05	-0.67	4.32E-03	-0.30	0.33	N-acetylneuraminate synthase	protein coding
ENSG00000143155	TIPRL	1	0.56	0.03	-0.07	0.82	-0.15	NA	TOR signaling pathway regulator	protein coding
ENSG00000065518	NDUFB4	3	0.56	0.03	-0.13	0.62	-0.25	0.36	NADH:ubiquinone oxidoreductase subunit B4	protein coding
ENSG00000156860	FBRS	16	0.56	3.34E-03	-0.49	3.36E-03	-0.46	0.05	fibrosin	protein coding
ENSG00000137806	NDUFAF1	15	0.56	0.04	-0.63	3.78E-03	-0.38	NA	NADH:ubiquinone oxidoreductase complex assembly factor 1	protein coding
ENSG00000115233	PSMD14	2	0.56	0.02	-0.01	0.96	-0.17	0.50	proteasome 26S subunit, non-ATPase 14	protein coding
ENSG00000196924	FLNA	X	0.56	0.02	-0.10	0.71	-0.22	0.39	filamin A	protein coding
ENSG00000137106	GRHPR	9	0.56	0.01	-0.29	0.17	-0.19	0.46	glyoxylate and hydroxypyruvate reductase	protein coding
ENSG00000123131	PRDX4	X	0.55	0.04	-0.24	0.36	-0.09	NA	peroxiredoxin 4	protein coding
ENSG00000168439	STIP1	11	0.55	0.04	-0.23	0.38	-0.35	0.24	stress induced phosphoprotein 1	protein coding
ENSG00000114480	GBE1	3	0.55	0.03	-0.47	0.03	-0.26	0.34	1,4-alpha-glucan branching enzyme 1	protein coding
ENSG00000115091	ACTR3	2	0.55	0.05	-0.36	0.15	-0.24	0.41	ARP3 actin related protein 3 homolog	protein coding
ENSG00000173786	CNP	17	0.55	0.03	-0.20	0.43	-0.38	0.17	2',3'-cyclic nucleotide 3' phosphodiesterase	protein coding
ENSG00000019582	CD74	5	0.55	0.01	-0.13	0.61	-0.11	0.68	CD74 molecule	protein coding
ENSG00000144746	ARL6IP5	3	0.55	0.03	-0.05	0.88	-0.06	0.83	ADP ribosylation factor like GTPase 6 interacting protein 5	protein coding
ENSG00000132591	ERAL1	17	0.55	0.02	0.19	0.42	-0.06	NA	Era like 12S mitochondrial rRNA chaperone 1	protein coding
ENSG00000113811	SELENOK	3	0.55	0.03	-0.02	0.94	-0.26	NA	selenoprotein K	protein coding
ENSG00000127191	TRAF2	9	0.55	0.01	-0.40	0.04	-0.26	0.29	TNF receptor associated factor 2	protein coding
ENSG00000079931	MOXD1	6	0.54	0.03	-1.42	6.71E-14	-0.23	0.40	monooxygenase DBH like 1	protein coding
ENSG00000114354	TFG	3	0.54	0.05	-0.28	0.26	-0.34	0.25	TRK-fused gene	protein coding
ENSG00000265241	RBM8A	1	0.54	0.02	0.03	0.93	-0.24	0.32	RNA binding motif protein 8A	protein coding
ENSG00000113558	SKP1	5	0.54	0.03	0.08	0.78	-0.03	0.92	S-phase kinase associated protein 1	protein coding
ENSG00000165283	STOML2	9	0.54	0.02	0.18	0.45	-0.05	0.87	stomatin like 2	protein coding
ENSG00000104549	SQLE	8	0.54	0.02	-0.43	0.03	-0.24	NA	squalene epoxidase	protein coding
ENSG00000184162	NR2C2AP	19	0.54	0.02	-0.16	0.50	-0.05	NA	nuclear receptor 2C2 associated protein	protein coding
ENSG00000074800	ENO1	1	0.53	0.04	-0.05	0.89	-0.04	0.90	enolase 1	protein coding
ENSG00000154719	MRPL39	21	0.53	0.02	-0.08	0.76	-0.13	NA	mitochondrial ribosomal protein L39	protein coding
ENSG00000084733	RAB10	2	0.53	0.03	0.01	0.97	-0.19	0.50	RAB10, member RAS oncogene family	protein coding
ENSG00000101367	MAPRE1	20	0.53	0.02	-0.15	0.53	-0.10	0.71	microtubule associated protein RP/EB family member 1	protein coding

ENSG00000184209	SNRNP35	12	0.53	0.04	-0.30	0.20	-0.30	NA	small nuclear ribonucleoprotein U11/U12 subunit 35	protein coding
ENSG00000074416	MGLL	3	0.53	0.04	-1.47	3.43E-14	-0.37	0.19	monoglyceride lipase	protein coding
ENSG00000060642	PIGV	1	0.53	0.04	-0.66	1.57E-03	-0.23	NA	phosphatidylinositol glycan anchor biosynthesis class V	protein coding
ENSG00000102309	PIN4	X	0.53	0.02	-0.75	2.37E-05	-0.14	NA	peptidylprolyl cis/trans isomerase, NIMA-interacting 4	protein coding
ENSG00000117448	AKR1A1	1	0.53	0.01	0.30	0.13	-0.05	0.86	aldo-keto reductase family 1 member A1	protein coding
ENSG00000198742	SMURF1	7	0.53	0.03	-0.40	0.07	-0.65	0.02	SMAD specific E3 ubiquitin protein ligase 1	protein coding
ENSG00000079246	XRCC5	2	0.53	0.04	-0.07	0.81	-0.12	0.70	X-ray repair cross complementing 5	protein coding
ENSG00000164053	ATRIP	3	0.53	0.02	-0.28	0.16	-0.47	NA	ATR interacting protein	protein coding
ENSG00000150753	CCT5	5	0.52	0.01	-0.21	0.29	-0.24	0.29	chaperonin containing TCP1 subunit 5	protein coding
ENSG00000155368	DBI	2	0.52	0.03	-0.43	0.04	-0.20	0.42	diazepam binding inhibitor, acyl-CoA binding protein	protein coding
ENSG00000133935	C14orf1	14	0.52	0.02	0.05	0.86	-0.11	NA	chromosome 14 open reading frame 1	protein coding
ENSG00000134287	ARF3	12	0.52	0.02	-0.20	0.36	0.02	0.95	ADP ribosylation factor 3	protein coding
ENSG00000141543	EIF4A3	17	0.52	0.05	-0.11	0.71	-0.31	0.27	eukaryotic translation initiation factor 4A3	protein coding
ENSG00000092330	TINF2	14	0.52	0.04	0.12	0.67	-0.22	0.42	TERF1 interacting nuclear factor 2	protein coding
ENSG00000165502	RPL36AL	14	0.52	0.04	-0.11	0.70	-0.18	0.52	ribosomal protein L36a like	protein coding
ENSG00000068323	TFE3	X	0.51	0.01	-0.39	0.04	-0.54	0.03	transcription factor binding to IGHM enhancer 3	protein coding
ENSG00000170860	LSM3	3	0.51	0.04	-0.01	0.98	-0.19	NA	LSM3 homolog, U6 small nuclear RNA and mRNA degradation associated	protein coding
ENSG00000087077	TRIP6	7	0.51	0.03	-0.25	0.25	-0.27	0.28	thyroid hormone receptor interactor 6	protein coding
ENSG00000013275	PSMC4	19	0.51	0.01	-0.12	0.60	-0.16	0.50	proteasome 26S subunit, ATPase 4	protein coding
ENSG00000234127	TRIM26	6	0.51	0.04	-0.59	3.33E-03	-0.28	NA	tripartite motif containing 26	protein coding
ENSG00000117758	STX12	1	0.51	0.02	-0.41	0.04	-0.13	0.60	syntaxin 12	protein coding
ENSG00000123444	KBTBD4	11	0.51	0.03	-0.19	0.40	-0.10	NA	kelch repeat and BTB domain containing 4	protein coding
ENSG00000149428	HYOU1	11	0.51	0.01	0.11	0.62	-0.26	0.25	hypoxia up-regulated 1	protein coding
ENSG00000104320	NBN	8	0.51	0.04	-0.40	0.07	-0.40	0.15	nibrin	protein coding
ENSG00000273749	CYFIP1	15	0.51	2.23E-03	-0.64	3.91E-06	-0.26	0.18	cytoplasmic FMR1 interacting protein 1	protein coding
ENSG00000163159	VPS72	1	0.51	0.03	0.03	0.93	-0.27	NA	vacuolar protein sorting 72 homolog	protein coding
ENSG00000023902	PLEKHO1	1	0.51	0.01	-0.48	2.51E-03	-0.48	0.03	pleckstrin homology domain containing O1	protein coding
ENSG00000132792	CTNNBL1	20	0.51	0.03	-0.11	0.65	-0.14	0.58	catenin beta like 1	protein coding
ENSG00000186818	LILRB4	19	0.50	0.04	-1.09	1.86E-08	-0.36	0.20	leukocyte immunoglobulin like receptor B4	protein coding
ENSG00000084234	APLP2	11	0.50	3.41E-03	0.25	0.12	-0.19	0.32	amyloid beta precursor like protein 2	protein coding
ENSG00000116288	PARK7	1	0.50	0.05	-0.06	0.85	0.04	0.89	Parkinsonism associated deglycase	protein coding
ENSG00000214717	ZBED1	X	0.50	5.80E-04	-0.23	0.10	-0.23	0.19	zinc finger BED-type containing 1	protein coding
ENSG00000101310	SEC23B	20	0.50	0.03	-0.20	0.39	-0.22	0.39	Sec23 homolog B, coat complex II component	protein coding
ENSG00000198522	GPN1	2	0.50	0.04	-0.10	0.71	0.10	NA	GPN-loop GTPase 1	protein coding
ENSG00000136141	LRCH1	13	0.50	0.03	-0.45	0.02	-0.30	NA	leucine rich repeats and calponin homology domain containing 1	protein coding
ENSG00000163399	ATP1A1	1	0.50	0.02	0.04	0.87	-0.04	0.86	ATPase Na+/K+ transporting subunit alpha 1	protein coding
ENSG00000163468	CCT3	1	0.50	0.02	-0.04	0.88	-0.08	0.73	chaperonin containing TCP1 subunit 3	protein coding
ENSG00000136930	PSMB7	9	0.49	0.03	-0.11	0.66	-0.04	0.88	proteasome subunit beta 7	protein coding
ENSG00000131507	NDFIP1	5	0.49	0.04	0.00	0.99	-0.04	0.88	Nedd4 family interacting protein 1	protein coding
ENSG00000078808	SDF4	1	0.49	1.65E-03	0.17	0.28	-0.12	0.52	stromal cell derived factor 4	protein coding

ENSG00000159352	PSMD4	1	0.49	0.01	-0.35	0.03	-0.05	0.82	proteasome 26S subunit, non-ATPase 4	protein coding
ENSG00000163719	MTMR14	3	0.49	0.02	-0.61	3.03E-04	-0.27	0.24	myotubularin related protein 14	protein coding
ENSG00000169976	SF3B5	6	0.49	0.03	-0.06	0.82	-0.03	0.92	splicing factor 3b subunit 5	protein coding
ENSG00000092010	PSME1	14	0.49	0.05	-0.38	0.08	-0.10	0.72	proteasome activator subunit 1	protein coding
ENSG00000111859	NEDD9	6	0.49	0.02	-0.63	1.53E-04	-0.33	0.15	neural precursor cell expressed, developmentally down-regulated 9	protein coding
ENSG00000109790	KLHL5	4	0.48	0.04	-0.30	0.15	-0.35	NA	kelch like family member 5	protein coding
ENSG00000103932	RPAP1	15	0.48	0.04	0.03	0.91	-0.19	NA	RNA polymerase II associated protein 1	protein coding
ENSG00000178719	GRINA	8	0.48	4.36E-03	-0.29	0.07	-0.04	0.85	glutamate ionotropic receptor NMDA type subunit associated protein 1	protein coding
ENSG00000205352	PRR13	12	0.48	0.01	-0.23	0.17	-0.12	0.54	proline rich 13	protein coding
ENSG00000173418	NAA20	20	0.48	0.04	-0.11	0.65	-0.08	NA	N(alpha)-acetyltransferase 20, NatB catalytic subunit	protein coding
ENSG00000181924	COA4	11	0.48	0.03	0.13	0.56	-0.14	NA	cytochrome c oxidase assembly factor 4 homolog	protein coding
ENSG00000010270	STARD3NL	7	0.48	0.04	0.18	0.44	-0.18	0.47	STARD3 N-terminal like	protein coding
ENSG00000101363	MANBAL	20	0.48	0.04	-0.16	0.50	-0.35	NA	mannosidase beta like	protein coding
ENSG00000163964	PIGX	3	0.47	0.02	-0.13	0.52	-0.20	0.36	phosphatidylinositol glycan anchor biosynthesis class X	protein coding
ENSG00000141741	MIEN1	17	0.47	0.02	-0.21	0.27	-0.21	NA	migration and invasion enhancer 1	protein coding
ENSG00000180353	HCLS1	3	0.47	0.01	-0.83	2.11E-09	-0.11	0.59	hematopoietic cell-specific Lyn substrate 1	protein coding
ENSG00000265148	TSPOAP1-AS1	17	0.47	0.03	-0.84	7.16E-07	-0.34	NA	TSPOAP1 antisense RNA 1	antisense
ENSG00000165168	CYBB	X	0.47	0.05	-0.40	0.05	-0.07	0.82	cytochrome b-245 beta chain	protein coding
ENSG00000116863	ADPRHL2	1	0.47	0.03	-0.10	0.66	-0.11	NA	ADP-ribosylhydrolase like 2	protein coding
ENSG00000102172	SMS	X	0.46	0.04	-0.51	0.01	-0.16	0.52	spermine synthase	protein coding
ENSG00000185753	CXorf38	X	0.46	0.04	-0.08	0.75	-0.41	0.11	chromosome X open reading frame 38	protein coding
ENSG00000128708	HAT1	2	0.46	0.02	-0.03	0.89	-0.09	NA	histone acetyltransferase 1	protein coding
ENSG00000131467	PSME3	17	0.46	0.01	0.10	0.62	-0.06	0.78	proteasome activator subunit 3	protein coding
ENSG00000033050	ABCF2	7	0.45	0.01	0.22	0.20	-0.08	0.69	ATP binding cassette subfamily F member 2	protein coding
ENSG00000169180	XPO6	16	0.45	0.02	-0.44	0.01	-0.18	0.39	exportin 6	protein coding
ENSG00000169813	HNRNPF	10	0.45	0.03	-0.06	0.79	-0.12	0.61	heterogeneous nuclear ribonucleoprotein F	protein coding
ENSG00000097033	SH3GLB1	1	0.45	0.04	-0.01	0.97	-0.19	0.42	SH3 domain containing GRB2 like endophilin B1	protein coding
ENSG00000117395	EBNA1BP2	1	0.45	0.03	-0.06	0.80	-0.05	NA	EBNA1 binding protein 2	protein coding
ENSG00000196419	XRCC6	22	0.45	0.04	0.14	0.54	-0.04	0.89	X-ray repair cross complementing 6	protein coding
ENSG00000166925	TSC22D4	7	0.45	0.02	-0.30	0.08	-0.22	0.30	TSC22 domain family member 4	protein coding
ENSG00000196352	CD55	1	0.45	0.03	-0.48	4.64E-03	-0.16	0.47	CD55 molecule (Cromer blood group)	protein coding
ENSG00000046653	GPM6B	X	0.45	2.68E-03	-0.76	1.99E-10	-0.28	NA	glycoprotein M6B	protein coding
ENSG00000011132	APBA3	19	0.44	0.02	-0.03	0.91	-0.28	0.19	amyloid beta precursor protein binding family A member 3	protein coding
ENSG00000132475	H3F3B	17	0.44	0.05	-0.04	0.88	-0.24	0.33	H3 histone family member 3B	protein coding
ENSG00000100462	PRMT5	14	0.44	0.02	-0.04	0.87	-0.12	0.57	protein arginine methyltransferase 5	protein coding
ENSG00000118260	CREB1	2	0.44	0.01	-0.19	0.24	-0.14	0.45	cAMP responsive element binding protein 1	protein coding
ENSG00000182220	ATP6AP2	X	0.44	0.03	0.30	0.10	0.05	0.83	ATPase H+ transporting accessory protein 2	protein coding
ENSG00000115839	RAB3GAP1	2	0.44	0.03	-0.27	0.15	-0.22	0.32	RAB3 GTPase activating protein catalytic subunit 1	protein coding
ENSG00000197147	LRRC8B	1	0.44	0.03	-0.32	0.06	-0.19	NA	leucine rich repeat containing 8 family member B	protein coding
ENSG00000102103	PQBP1	X	0.43	0.01	-0.06	0.77	-0.05	0.80	polyglutamine binding protein 1	protein coding

ENSG00000135624	CCT7	2	0.43	0.05	-0.58	1.06E-03	-0.03	0.90	chaperonin containing TCP1 subunit 7	protein coding
ENSG00000124226	RNF114	20	0.43	0.03	-0.17	0.39	-0.23	0.29	ring finger protein 114	protein coding
ENSG00000102144	PGK1	X	0.43	0.05	-0.20	0.35	0.02	0.93	phosphoglycerate kinase 1	protein coding
ENSG00000100764	PSMC1	14	0.43	0.03	0.13	0.53	-0.22	0.29	proteasome 26S subunit, ATPase 1	protein coding
ENSG00000141959	PFKL	21	0.43	0.02	-0.27	0.10	-0.16	0.42	phosphofructokinase, liver type	protein coding
ENSG00000100225	FBXO7	22	0.43	0.05	-0.05	0.84	-0.13	0.60	F-box protein 7	protein coding
ENSG00000129460	NGDN	14	0.43	0.01	0.13	0.45	-0.12	NA	neuroguidin	protein coding
ENSG00000148572	NRBF2	10	0.43	0.03	0.04	0.86	-0.10	NA	nuclear receptor binding factor 2	protein coding
ENSG00000143570	SLC39A1	1	0.42	0.03	0.12	0.54	-0.02	0.94	solute carrier family 39 member 1	protein coding
ENSG00000143420	ENSA	1	0.42	0.03	-0.42	0.01	-0.19	0.37	endosulfine alpha	protein coding
ENSG00000158796	DEDD	1	0.42	3.11E-03	-0.15	0.26	-0.27	0.11	death effector domain containing	protein coding
ENSG00000165271	NOL6	9	0.42	0.05	0.28	0.13	-0.27	0.25	nucleolar protein 6	protein coding
ENSG00000121067	SPOP	17	0.41	0.02	-0.11	0.57	-0.04	0.85	speckle type BTB/POZ protein	protein coding
ENSG00000092199	HNRNPC	14	0.41	0.05	-0.05	0.85	-0.17	0.46	heterogeneous nuclear ribonucleoprotein C (C1/C2)	protein coding
ENSG00000275700	AATF	17	0.41	0.05	-0.31	0.09	-0.04	NA	apoptosis antagonizing transcription factor	protein coding
ENSG00000174173	TRMT10C	3	0.41	0.03	-0.17	0.37	-0.21	NA	tRNA methyltransferase 10C, mitochondrial RNase P subunit	protein coding
ENSG00000141965	FEM1A	19	0.41	0.02	0.06	0.76	-0.20	0.30	fem-1 homolog A	protein coding
ENSG00000173692	PSMD1	2	0.41	0.03	0.02	0.92	-0.12	0.59	proteasome 26S subunit, non-ATPase 1	protein coding
ENSG00000004455	AK2	1	0.41	0.04	0.20	0.31	0.01	0.98	adenylate kinase 2	protein coding
ENSG00000198561	CTNND1	11	0.40	0.02	0.29	0.07	-0.20	0.30	catenin delta 1	protein coding
ENSG00000126067	PSMB2	1	0.40	0.03	0.04	0.84	-0.10	0.64	proteasome subunit beta 2	protein coding
ENSG00000087191	PSMC5	17	0.40	0.02	-0.05	0.81	-0.18	0.33	proteasome 26S subunit, ATPase 5	protein coding
ENSG00000150977	RILPL2	12	0.39	0.03	0.12	0.54	-0.27	0.19	Rab interacting lysosomal protein like 2	protein coding
ENSG00000163832	ELP6	3	0.39	0.03	-0.35	0.03	-0.04	NA	elongator acetyltransferase complex subunit 6	protein coding
ENSG00000141510	TP53	17	0.39	0.05	-0.02	0.95	-0.01	0.98	tumor protein p53	protein coding
ENSG00000074319	TSG101	11	0.39	0.02	-0.33	0.02	-0.19	0.30	tumor susceptibility 101	protein coding
ENSG00000125970	RALY	20	0.39	9.80E-04	-0.30	4.04E-03	-0.09	0.52	RALY heterogeneous nuclear ribonucleoprotein	protein coding
ENSG00000167986	DDB1	11	0.39	0.05	0.21	0.27	0.01	0.97	damage specific DNA binding protein 1	protein coding
ENSG00000090273	NUDC	1	0.38	0.02	0.08	0.68	-0.03	0.89	nuclear distribution C, dynein complex regulator	protein coding
ENSG00000130402	ACTN4	19	0.38	0.01	-0.35	0.01	-0.16	0.33	actinin alpha 4	protein coding
ENSG00000183431	SF3A3	1	0.38	0.04	-0.16	0.36	-0.13	0.50	splicing factor 3a subunit 3	protein coding
ENSG00000140740	UQCRC2	16	0.37	0.04	-0.30	0.06	-0.05	0.81	ubiquinol-cytochrome c reductase core protein II	protein coding
ENSG00000125734	GPR108	19	0.37	0.03	-0.21	0.20	-0.12	0.54	G protein-coupled receptor 108	protein coding
ENSG00000119203	CPSF3	2	0.37	0.04	-0.08	0.69	-0.15	0.42	cleavage and polyadenylation specific factor 3	protein coding
ENSG00000149182	ARFGAP2	11	0.37	0.04	-0.01	0.96	0.05	0.83	ADP ribosylation factor GTPase activating protein 2	protein coding
ENSG00000115866	DARS	2	0.37	0.05	-0.27	0.10	-0.07	0.74	aspartyl-tRNA synthetase	protein coding
ENSG00000107862	GBF1	10	0.37	0.01	0.23	0.05	-0.21	0.18	golgi brefeldin A resistant guanine nucleotide exchange factor 1	protein coding
ENSG00000204160	ZDHHC18	1	0.36	0.01	-0.08	0.63	-0.25	0.14	zinc finger DHHC-type containing 18	protein coding
ENSG00000161847	RAVER1	19	0.36	0.02	0.19	0.19	-0.19	0.29	ribonucleoprotein, PTB binding 1	protein coding
ENSG00000151779	NBAS	2	0.36	0.03	-0.26	0.09	-0.06	0.74	neuroblastoma amplified sequence	protein coding
ENSG00000164889	SLC4A2	7	0.36	0.05	0.03	0.90	-0.16	0.41	solute carrier family 4 member 2	protein coding

ENSG00000167881	SRP68	17	0.36	0.05	0.21	0.19	-0.01	0.95	signal recognition particle 68	protein coding
ENSG00000103496	STX4	16	0.36	0.05	-0.37	0.01	-0.38	0.07	syntaxin 4	protein coding
ENSG00000119487	MAPKAP1	9	0.36	0.01	-0.07	0.68	-0.14	0.38	mitogen-activated protein kinase associated protein 1	protein coding
ENSG00000147654	EBAG9	8	0.35	0.05	-0.42	3.28E-03	-0.19	NA	estrogen receptor binding site associated, antigen, 9	protein coding
ENSG00000173039	RELA	11	0.34	0.02	0.09	0.59	-0.18	0.28	RELA proto-oncogene, NF-kB subunit	protein coding
ENSG00000126698	DNAJC8	1	0.34	0.04	-0.09	0.57	-0.16	0.35	DnaJ heat shock protein family (Hsp40) member C8	protein coding
ENSG00000204463	BAG6	6	0.33	0.04	-0.08	0.64	-0.02	0.94	BCL2 associated athanogene 6	protein coding
ENSG00000164253	WDR41	5	0.33	0.03	-0.08	0.64	-0.06	0.73	WD repeat domain 41	protein coding
ENSG00000140350	ANP32A	15	0.33	0.04	-0.03	0.87	-0.09	0.61	acidic nuclear phosphoprotein 32 family member A	protein coding
ENSG00000162521	RBBP4	1	0.33	0.04	-0.21	0.13	-0.09	0.60	RB binding protein 4, chromatin remodeling factor	protein coding
ENSG00000105401	CDC37	19	0.33	0.03	0.04	0.81	-0.20	0.23	cell division cycle 37	protein coding
ENSG00000110497	AMBRA1	11	0.33	0.04	0.25	0.05	-0.10	0.58	autophagy and beclin 1 regulator 1	protein coding
ENSG00000189091	SF3B3	16	0.32	0.04	0.24	0.09	0.01	0.95	splicing factor 3b subunit 3	protein coding
ENSG00000126581	BECN1	17	0.32	0.01	0.24	0.02	-0.12	0.36	beclin 1	protein coding
ENSG00000108344	PSMD3	17	0.32	0.03	0.11	0.48	0.03	0.87	proteasome 26S subunit, non-ATPase 3	protein coding
ENSG00000132128	LRRC41	1	0.32	0.05	0.01	0.95	-0.11	0.51	leucine rich repeat containing 41	protein coding
ENSG00000172943	PHF8	X	0.31	0.03	-0.26	0.03	-0.12	0.42	PHD finger protein 8	protein coding
ENSG00000144580	CNOT9	2	0.31	0.02	0.05	0.76	-0.07	0.64	CCR4-NOT transcription complex subunit 9	protein coding
ENSG00000116688	MFN2	1	0.31	0.05	-0.04	0.82	-0.12	0.48	mitofusin 2	protein coding
ENSG00000101337	TM9SF4	20	0.30	0.03	0.36	1.59E-03	-0.08	0.64	transmembrane 9 superfamily member 4	protein coding
ENSG00000179562	GCC1	7	0.30	0.04	0.17	0.20	-0.03	0.88	GRIP and coiled-coil domain containing 1	protein coding
ENSG00000105058	FAM32A	19	0.29	0.05	0.24	0.06	-0.10	0.52	family with sequence similarity 32 member A	protein coding
ENSG00000106244	PDAP1	7	0.27	0.04	0.03	0.87	-0.14	0.30	PDGFA associated protein 1	protein coding
ENSG00000092201	SUPT16H	14	0.25	0.05	-0.12	0.33	-0.09	0.54	SPT16 homolog, facilitates chromatin remodeling subunit	protein coding
ENSG00000143157	POGK	1	-0.23	0.03	0.40	8.22E-07	0.22	0.07	pogo transposable element with KRAB domain	protein coding
ENSG00000101126	ADNP	20	-0.25	0.05	0.03	0.86	0.05	0.73	activity dependent neuroprotector homeobox	protein coding
ENSG00000225733	FGD5-AS1	3	-0.26	0.05	0.53	6.98E-08	0.11	0.41	FGD5 antisense RNA 1	antisense
ENSG00000067955	CBFB	16	-0.32	0.04	0.35	0.01	0.31	0.08	core-binding factor beta subunit	protein coding
ENSG00000197102	DYNC1H1	14	-0.34	0.04	0.34	0.01	0.10	0.56	dynein cytoplasmic 1 heavy chain 1	protein coding
ENSG00000153250	RBMS1	2	-0.34	0.03	0.07	0.70	0.00	1.00	RNA binding motif single stranded interacting protein 1	protein coding
ENSG00000066422	ZBTB11	3	-0.36	0.04	0.56	4.69E-05	0.31	0.12	zinc finger and BTB domain containing 11	protein coding
ENSG00000187257	RSBN1L	7	-0.37	0.04	0.27	0.10	0.13	0.51	round spermatid basic protein 1 like	protein coding
ENSG00000086102	NFX1	9	-0.38	0.01	0.05	0.78	0.08	0.68	nuclear transcription factor, X-box binding 1	protein coding
ENSG00000110851	PRDM4	12	-0.39	0.02	0.20	0.23	0.08	NA	PR/SET domain 4	protein coding
ENSG00000188895	MSL1	17	-0.39	0.02	0.27	0.08	0.06	0.79	male specific lethal 1 homolog	protein coding
ENSG00000163950	SLBP	4	-0.40	0.01	0.32	0.01	0.26	0.12	stem-loop binding protein	protein coding
ENSG00000102606	ARHGEF7	13	-0.41	0.03	0.22	0.23	0.24	0.27	Rho guanine nucleotide exchange factor 7	protein coding
ENSG00000179456	ZBTB18	1	-0.42	0.01	0.03	0.90	0.10	0.59	zinc finger and BTB domain containing 18	protein coding
ENSG00000110066	KMT5B	11	-0.42	0.03	0.21	0.25	0.31	0.17	lysine methyltransferase 5B	protein coding
ENSG00000017797	RALBP1	18	-0.42	0.04	0.76	2.01E-06	0.37	0.11	ralA binding protein 1	protein coding
ENSG00000138604	GLCE	15	-0.42	0.04	0.26	0.17	0.27	NA	glucuronic acid epimerase	protein coding

ENSG00000163960	UBXN7	3	-0.42	0.01	0.49	2.41E-04	0.28	0.14	UBX domain protein 7	protein coding
ENSG00000215114	UBXN2B	8	-0.43	0.01	0.78	4.92E-09	0.29	NA	UBX domain protein 2B	protein coding
ENSG00000140577	CRTC3	15	-0.44	0.03	0.41	0.02	0.39	0.10	CREB regulated transcription coactivator 3	protein coding
ENSG00000165512	ZNF22	10	-0.45	0.03	-0.04	0.87	0.19	0.39	zinc finger protein 22	protein coding
ENSG00000109787	KLF3	4	-0.45	0.05	0.41	0.03	0.35	NA	Kruppel like factor 3	protein coding
ENSG00000169967	MAP3K2	2	-0.45	0.02	0.40	0.02	0.20	0.36	mitogen-activated protein kinase kinase kinase 2	protein coding
ENSG00000083844	ZNF264	19	-0.46	0.02	0.65	2.87E-05	0.23	NA	zinc finger protein 264	protein coding
ENSG00000152443	ZNF776	19	-0.46	0.04	0.53	4.77E-03	0.24	NA	zinc finger protein 776	protein coding
ENSG00000107140	TESK1	9	-0.46	0.02	0.23	0.23	0.07	0.77	testis-specific kinase 1	protein coding
ENSG00000077684	JADE1	4	-0.46	0.03	-0.15	0.49	0.34	NA	jade family PHD finger 1	protein coding
ENSG00000141298	SSH2	17	-0.47	0.04	0.48	0.01	0.29	0.25	slingshot protein phosphatase 2	protein coding
ENSG00000143614	GATAD2B	1	-0.47	0.05	0.52	0.01	0.38	NA	GATA zinc finger domain containing 2B	protein coding
ENSG00000182606	TRAK1	3	-0.47	0.04	0.19	0.42	0.10	0.71	trafficking kinesin protein 1	protein coding
ENSG00000152520	PAN3	13	-0.48	0.04	-0.26	0.24	0.17	0.50	PAN3 poly(A) specific ribonuclease subunit	protein coding
ENSG00000130305	NSUN5	7	-0.48	0.02	0.68	4.17E-05	0.46	NA	NOP2/Sun RNA methyltransferase family member 5	protein coding
ENSG00000033867	SLC4A7	3	-0.48	0.04	-0.19	0.42	0.43	NA	solute carrier family 4 member 7	protein coding
ENSG00000095585	BLNK	10	-0.49	0.01	0.60	2.32E-04	0.31	0.17	B-cell linker	protein coding
ENSG00000198363	ASPH	8	-0.49	0.04	0.33	0.12	0.22	0.39	aspartate beta-hydroxylase	protein coding
ENSG00000172845	SP3	2	-0.49	1.28E-03	0.34	0.01	0.27	0.14	Sp3 transcription factor	protein coding
ENSG00000266094	RASSF5	1	-0.50	0.03	0.36	0.08	0.41	0.12	Ras association domain family member 5	protein coding
ENSG00000141582	CBX4	17	-0.50	0.05	-0.08	0.78	0.29	0.29	chromobox 4	protein coding
ENSG00000177888	ZBTB41	1	-0.51	0.04	0.65	1.25E-03	0.24	NA	zinc finger and BTB domain containing 41	protein coding
ENSG00000121741	ZMYM2	13	-0.51	0.02	0.22	0.28	0.05	0.86	zinc finger MYM-type containing 2	protein coding
ENSG00000163428	LRRC58	3	-0.51	0.04	0.64	2.01E-03	0.43	0.14	leucine rich repeat containing 58	protein coding
ENSG00000135974	C2orf49	2	-0.51	0.03	0.33	0.12	0.15	NA	chromosome 2 open reading frame 49	protein coding
ENSG00000170456	DENND5B	12	-0.51	0.04	0.35	0.11	0.24	0.37	DENN domain containing 5B	protein coding
ENSG00000141232	TOB1	17	-0.52	0.01	0.10	0.66	0.20	NA	transducer of ERBB2, 1	protein coding
ENSG00000135966	TGFBRAP1	2	-0.52	0.05	0.55	0.01	0.37	0.20	transforming growth factor beta receptor associated protein 1	protein coding
ENSG00000158158	CNNM4	2	-0.52	0.02	0.47	0.01	0.34	NA	cyclin and CBS domain divalent metal cation transport mediator 4	protein coding
ENSG00000166529	ZSCAN21	7	-0.52	0.04	0.72	4.84E-04	0.32	NA	zinc finger and SCAN domain containing 21	protein coding
ENSG00000198894	CIPC	14	-0.52	0.05	0.84	5.13E-05	0.22	NA	CLOCK interacting pacemaker	protein coding
ENSG00000163602	RYBP	3	-0.52	0.01	0.72	6.86E-06	0.50	0.03	RING1 and YY1 binding protein	protein coding
ENSG00000151498	ACAD8	11	-0.53	0.03	0.11	0.70	0.18	NA	acyl-CoA dehydrogenase family member 8	protein coding
ENSG00000081177	EXD2	14	-0.53	0.04	0.09	0.75	0.74	NA	exonuclease 3'-5' domain containing 2	protein coding
ENSG00000108055	SMC3	10	-0.53	0.03	0.49	0.02	0.24	0.36	structural maintenance of chromosomes 3	protein coding
ENSG00000136108	CKAP2	13	-0.54	0.04	1.00	1.23E-06	0.51	0.09	cytoskeleton associated protein 2	protein coding
ENSG00000197930	ERO1A	14	-0.54	0.03	0.34	0.12	0.28	0.29	endoplasmic reticulum oxidoreductase 1 alpha	protein coding
ENSG00000213390	ARHGAP19	10	-0.54	0.03	0.04	0.91	0.45	NA	Rho GTPase activating protein 19	protein coding
ENSG00000153487	ING1	13	-0.54	0.03	0.29	0.23	0.27	NA	inhibitor of growth family member 1	protein coding
ENSG00000112406	HECA	6	-0.55	0.03	0.46	0.03	0.27	0.31	hdc homolog, cell cycle regulator	protein coding

ENSG00000099940	SNAP29	22	-0.55	0.03	0.96	2.09E-06	0.49	0.10	synaptosome associated protein 29	protein coding
ENSG00000198586	TLK1	2	-0.55	0.02	-0.19	0.43	0.09	0.75	tousled like kinase 1	protein coding
ENSG00000173011	TADA2B	4	-0.55	0.03	1.01	1.47E-07	0.43	0.12	transcriptional adaptor 2B	protein coding
ENSG00000134262	AP4B1	1	-0.55	0.02	0.14	0.57	0.45	NA	adaptor related protein complex 4 beta 1 subunit	protein coding
ENSG00000145029	NICN1	3	-0.55	0.03	-0.22	0.41	0.10	NA	nicolin 1	protein coding
ENSG00000164975	SNAPC3	9	-0.56	0.03	0.47	0.03	0.36	NA	small nuclear RNA activating complex polypeptide 3	protein coding
ENSG00000100711	ZFYVE21	14	-0.56	0.04	0.58	0.01	0.32	NA	zinc finger FYVE-type containing 21	protein coding
ENSG00000112234	FBXL4	6	-0.56	0.04	0.64	4.02E-03	0.22	NA	F-box and leucine rich repeat protein 4	protein coding
ENSG00000100485	SOS2	14	-0.56	0.02	0.68	7.14E-04	0.40	0.15	SOS Ras/Rho guanine nucleotide exchange factor 2	protein coding
ENSG00000140455	USP3	15	-0.57	4.81E-04	0.53	1.28E-04	0.49	NA	ubiquitin specific peptidase 3	protein coding
ENSG00000130766	SESN2	1	-0.57	0.03	0.55	0.01	0.66	NA	sestrin 2	protein coding
ENSG00000121104	FAM117A	17	-0.57	0.04	0.19	0.51	0.33	0.28	family with sequence similarity 117 member A	protein coding
ENSG00000168936	TMEM129	4	-0.58	0.04	0.40	0.10	0.32	0.29	transmembrane protein 129	protein coding
ENSG00000106415	GLCCI1	7	-0.58	0.03	-0.66	3.34E-03	0.32	0.28	glucocorticoid induced 1	protein coding
ENSG00000120868	APAF1	12	-0.58	0.01	0.21	0.33	0.26	0.28	apoptotic peptidase activating factor 1	protein coding
ENSG00000109118	PHF12	17	-0.58	0.04	0.41	0.10	0.14	0.66	PHD finger protein 12	protein coding
ENSG00000108262	GIT1	17	-0.58	0.03	0.45	0.05	0.24	0.39	GIT ArfGAP 1	protein coding
ENSG00000170802	FOXN2	2	-0.59	0.02	0.34	0.15	0.51	0.09	forkhead box N2	protein coding
ENSG00000185798	WDR53	3	-0.59	0.04	0.74	1.16E-03	0.18	NA	WD repeat domain 53	protein coding
ENSG00000181472	ZBTB2	6	-0.59	4.81E-04	0.77	4.94E-08	0.54	NA	zinc finger and BTB domain containing 2	protein coding
ENSG00000057935	MTA3	2	-0.59	0.05	0.78	1.52E-03	0.17	NA	metastasis associated 1 family member 3	protein coding
ENSG00000126775	ATG14	14	-0.59	0.03	0.59	0.01	-0.02	NA	autophagy related 14	protein coding
ENSG00000012983	MAP4K5	14	-0.60	0.02	0.57	0.01	0.04	NA	mitogen-activated protein kinase kinase kinase kinase 5	protein coding
ENSG00000163755	HPS3	3	-0.60	0.05	0.58	0.02	0.40	NA	HPS3, biogenesis of lysosomal organelles complex 2 subunit 1	protein coding
ENSG00000139718	SETD1B	12	-0.60	0.03	1.06	1.94E-06	0.11	0.75	SET domain containing 1B	protein coding
ENSG00000143479	DYRK3	1	-0.60	0.01	1.21	1.02E-13	0.36	NA	dual specificity tyrosine phosphorylation regulated kinase 3	protein coding
ENSG00000172530	BANP	16	-0.61	0.01	-0.08	0.78	0.34	NA	BTG3 associated nuclear protein	protein coding
ENSG00000113851	CRBN	3	-0.61	3.98E-03	0.48	0.01	0.36	NA	cereblon	protein coding
ENSG00000173875	ZNF791	19	-0.61	0.01	0.70	5.03E-04	0.38	NA	zinc finger protein 791	protein coding
ENSG00000070476	ZXDC	3	-0.61	0.02	0.26	0.31	0.16	NA	ZXD family zinc finger C	protein coding
ENSG00000122678	POLM	7	-0.61	0.04	0.10	0.77	0.04	NA	DNA polymerase mu	protein coding
ENSG00000243646	IL10RB	21	-0.62	0.03	0.69	2.65E-03	0.41	NA	interleukin 10 receptor subunit beta	protein coding
ENSG00000117155	SSX2IP	1	-0.62	0.04	0.36	0.20	0.35	NA	SSX family member 2 interacting protein	protein coding
ENSG00000181827	RFX7	15	-0.62	3.31E-03	0.64	3.90E-04	0.50	0.06	regulatory factor X7	protein coding
ENSG00000188997	KCTD21	11	-0.63	0.02	0.54	0.02	0.33	NA	potassium channel tetramerization domain containing 21	protein coding
ENSG00000108312	UBTF	17	-0.63	0.02	0.11	0.73	0.52	0.10	upstream binding transcription factor, RNA polymerase I	protein coding
ENSG00000132405	TBC1D14	4	-0.63	0.04	0.56	0.03	0.48	0.16	TBC1 domain family member 14	protein coding
ENSG00000179918	SEPHS2	16	-0.63	0.05	0.40	0.18	0.67	0.07	selenophosphate synthetase 2	protein coding
ENSG00000134698	AGO4	1	-0.63	0.05	-0.23	0.48	0.19	NA	argonaute 4, RISC catalytic component	protein coding
ENSG00000184939	ZFP90	16	-0.63	0.02	1.39	3.26E-12	0.42	NA	ZFP90 zinc finger protein	protein coding

ENSG00000149091	DGKZ	11	-0.64	0.04	0.90	3.97E-04	0.35	0.30	diacylglycerol kinase zeta	protein coding
ENSG00000145990	GFOD1	6	-0.64	0.02	0.03	0.92	0.30	NA	glucose-fructose oxidoreductase domain containing 1	protein coding
ENSG00000166938	DIS3L	15	-0.64	0.01	0.34	0.14	0.34	NA	DIS3 like exosome 3'-5' exoribonuclease	protein coding
ENSG00000134324	LPIN1	2	-0.65	0.02	0.69	2.65E-03	0.43	0.17	lipin 1	protein coding
ENSG00000100027	YPEL1	22	-0.65	0.03	0.24	0.44	0.23	NA	yippee like 1	protein coding
ENSG00000111670	GNPTAB	12	-0.65	0.02	0.19	0.51	0.38	0.22	N-acetylglucosamine-1-phosphate transferase alpha and beta subunits	protein coding
ENSG00000149485	FADS1	11	-0.65	0.04	0.37	0.22	0.62	NA	fatty acid desaturase 1	protein coding
ENSG00000173950	XXYLT1	3	-0.65	0.01	0.44	0.04	0.50	NA	xyloside xylosyltransferase 1	protein coding
ENSG00000162889	MAPKAPK2	1	-0.65	0.03	0.39	0.16	0.52	0.12	mitogen-activated protein kinase-activated protein kinase 2	protein coding
ENSG00000132334	PTPRE	10	-0.65	0.04	0.38	0.19	0.49	0.16	protein tyrosine phosphatase, receptor type E	protein coding
ENSG00000174839	DENND6A	3	-0.66	0.03	0.42	0.13	-0.07	NA	DENN domain containing 6A	protein coding
ENSG00000181666	HKR1	19	-0.66	0.03	0.76	1.94E-03	0.21	NA	HKR1, GLI-Kruppel zinc finger family member	protein coding
ENSG00000146281	PM20D2	6	-0.67	0.02	1.01	2.46E-06	0.55	NA	peptidase M20 domain containing 2	protein coding
ENSG00000183688	RFLNB	17	-0.67	0.05	0.32	0.32	0.74	0.06	refilin B	protein coding
ENSG00000146263	MMS22L	6	-0.67	0.01	0.05	0.90	0.22	NA	MMS22 like, DNA repair protein	protein coding
ENSG00000159110	IFNAR2	21	-0.67	0.01	0.45	0.06	0.40	0.18	interferon alpha and beta receptor subunit 2	protein coding
ENSG00000149212	SESN3	11	-0.68	0.04	0.04	0.93	0.36	NA	sestrin 3	protein coding
ENSG00000182504	CEP97	3	-0.68	0.02	0.64	0.01	0.37	NA	centrosomal protein 97	protein coding
ENSG00000178904	DPY19L3	19	-0.68	0.03	0.02	0.96	0.18	NA	dpy-19 like 3 (C, elegans)	protein coding
ENSG00000106479	ZNF862	7	-0.68	0.05	0.71	0.01	0.11	NA	zinc finger protein 862	protein coding
ENSG00000205730	ITPRIPL2	16	-0.68	0.01	-0.25	0.33	0.31	NA	inositol 1,4,5-trisphosphate receptor interacting protein like 2	protein coding
ENSG00000167202	TBC1D2B	15	-0.68	0.04	0.38	0.23	0.34	0.35	TBC1 domain family member 2B	protein coding
ENSG00000162437	RAVER2	1	-0.68	0.03	1.14	4.30E-06	0.42	NA	ribonucleoprotein, PTB binding 2	protein coding
ENSG00000153140	CETN3	5	-0.69	0.04	-0.44	0.16	0.37	NA	centrin 3	protein coding
ENSG00000162636	FAM102B	1	-0.70	0.01	0.16	0.59	0.61	NA	family with sequence similarity 102 member B	protein coding
ENSG00000260822	GS1-358P8,4	X	-0.70	0.03	0.81	2.01E-03	0.53	NA		sense overlapping
ENSG00000204859	ZBTB48	1	-0.70	0.05	0.42	0.20	0.21	NA	zinc finger and BTB domain containing 48	protein coding
ENSG00000136451	VEZF1	17	-0.70	9.96E-09	0.52	5.81E-06	0.38	0.02	vascular endothelial zinc finger 1	protein coding
ENSG00000162378	ZYG11B	1	-0.70	0.04	0.97	5.90E-04	0.58	NA	zyg-11 family member B, cell cycle regulator	protein coding
ENSG00000082438	COBLL1	2	-0.70	0.02	0.35	0.20	0.37	0.26	cordon-bleu WH2 repeat protein like 1	protein coding
ENSG00000189308	LIN54	4	-0.71	0.03	0.50	0.07	0.43	NA	lin-54 DREAM MuvB core complex component	protein coding
ENSG00000141858	SAMD1	19	-0.71	0.02	0.33	0.26	0.47	NA	sterile alpha motif domain containing 1	protein coding
ENSG00000167106	FAM102A	9	-0.71	0.04	-0.05	0.90	0.59	NA	family with sequence similarity 102 member A	protein coding
ENSG00000103064	SLC7A6	16	-0.71	0.03	-0.52	0.08	0.28	0.43	solute carrier family 7 member 6	protein coding
ENSG00000177084	POLE	12	-0.71	0.01	-0.05	0.89	0.33	0.29	DNA polymerase epsilon, catalytic subunit	protein coding
ENSG00000196693	ZNF33B	10	-0.71	4.98E-03	0.58	0.01	0.30	NA	zinc finger protein 33B	protein coding
ENSG00000186951	PPARA	22	-0.71	0.01	0.27	0.32	0.37	0.23	peroxisome proliferator activated receptor alpha	protein coding
ENSG00000101190	TCFL5	20	-0.72	0.05	0.76	0.01	0.24	NA	transcription factor like 5	protein coding
ENSG00000167470	MIDN	19	-0.72	2.34E-03	0.12	0.68	0.36	NA	midnolin	protein coding
ENSG00000171492	LRRC8D	1	-0.72	0.04	0.49	0.13	0.68	0.10	leucine rich repeat containing 8 family member D	protein coding

ENSG00000163818	LZTFL1	3	-0.73	0.01	0.67	0.01	0.44	NA	leucine zipper transcription factor like 1	protein coding
ENSG00000168297	PXK	3	-0.73	0.03	0.88	1.10E-03	0.63	0.10	PX domain containing serine/threonine kinase like	protein coding
ENSG00000160271	RALGDS	9	-0.73	0.02	0.05	0.89	-0.01	NA	ral guanine nucleotide dissociation stimulator	protein coding
ENSG00000197008	ZNF138	7	-0.73	0.05	0.32	0.38	0.34	NA	zinc finger protein 138	protein coding
ENSG00000110721	CHKA	11	-0.73	0.02	0.32	0.30	0.51	NA	choline kinase alpha	protein coding
ENSG00000176014	TUBB6	18	-0.74	0.01	0.17	0.60	0.60	0.09	tubulin beta 6 class V	protein coding
ENSG00000178966	RMI1	9	-0.74	0.04	0.63	0.04	0.75	NA	RecQ mediated genome instability 1	protein coding
ENSG00000177169	ULK1	12	-0.74	0.05	0.53	0.12	0.30	0.46	unc-51 like autophagy activating kinase 1	protein coding
ENSG00000169826	CSGALNACT2	10	-0.74	4.81E-04	0.89	8.12E-07	0.25	0.31	chondroitin sulfate N-acetylgalactosaminyltransferase 2	protein coding
ENSG00000117625	RCOR3	1	-0.74	0.03	-0.24	0.52	0.31	NA	REST corepressor 3	protein coding
ENSG00000085644	ZNF213	16	-0.75	0.05	0.48	0.16	0.43	NA	zinc finger protein 213	protein coding
ENSG00000133874	RNF122	8	-0.75	0.03	0.77	0.01	0.76	NA	ring finger protein 122	protein coding
ENSG00000096654	ZNF184	6	-0.75	0.01	0.68	0.01	0.44	NA	zinc finger protein 184	protein coding
ENSG00000128923	MINDY2	15	-0.75	0.03	-0.14	0.72	0.43	NA	MINDY lysine 48 deubiquitinase 2	protein coding
ENSG00000103199	ZNF500	16	-0.75	0.02	0.59	0.04	0.27	NA	zinc finger protein 500	protein coding
ENSG00000114739	ACVR2B	3	-0.75	0.02	0.75	0.01	0.61	NA	activin A receptor type 2B	protein coding
ENSG00000059145	UNKL	16	-0.75	0.03	0.95	1.07E-03	0.22	NA	unkempt family like zinc finger	protein coding
ENSG00000164048	ZNF589	3	-0.76	0.05	0.29	0.45	0.06	0.89	zinc finger protein 589	protein coding
ENSG00000111554	MDM1	12	-0.76	0.01	0.70	0.01	0.15	NA	Mdm1 nuclear protein	protein coding
ENSG00000179833	SERTAD2	2	-0.76	0.04	0.69	0.03	0.62	0.14	SERTA domain containing 2	protein coding
ENSG00000198825	INPP5F	10	-0.77	0.02	-0.83	2.65E-03	0.18	0.63	inositol polyphosphate-5-phosphatase F	protein coding
ENSG00000140450	ARRDC4	15	-0.78	0.04	0.76	0.01	0.29	NA	arrestin domain containing 4	protein coding
ENSG00000011523	CEP68	2	-0.78	0.02	0.60	0.04	0.67	0.08	centrosomal protein 68	protein coding
ENSG00000084093	REST	4	-0.78	0.02	0.52	0.08	0.40	0.27	RE1 silencing transcription factor	protein coding
ENSG00000105643	ARRDC2	19	-0.79	0.05	0.07	0.88	0.37	NA	arrestin domain containing 2	protein coding
ENSG00000095015	MAP3K1	5	-0.80	4.51E-03	0.80	8.79E-04	0.61	0.08	mitogen-activated protein kinase kinase kinase 1	protein coding
ENSG00000107819	SFXN3	10	-0.80	0.02	-0.23	0.53	0.27	NA	sideroflexin 3	protein coding
ENSG00000181035	SLC25A42	19	-0.80	2.71E-03	0.75	1.22E-03	0.46	NA	solute carrier family 25 member 42	protein coding
ENSG00000165322	ARHGAP12	10	-0.80	0.04	0.40	0.29	0.41	NA	Rho GTPase activating protein 12	protein coding
ENSG00000121989	ACVR2A	2	-0.80	0.03	0.40	0.24	0.43	NA	activin A receptor type 2A	protein coding
ENSG00000167977	KCTD5	16	-0.81	1.69E-04	0.89	1.59E-06	0.58	0.03	potassium channel tetramerization domain containing 5	protein coding
ENSG00000064999	ANKS1A	6	-0.81	0.01	0.53	0.05	0.64	0.08	ankyrin repeat and sterile alpha motif domain containing 1A	protein coding
ENSG00000206560	ANKRD28	3	-0.82	0.02	-0.28	0.42	0.33	NA	ankyrin repeat domain 28	protein coding
ENSG00000118276	B4GALT6	18	-0.82	0.03	0.09	0.84	0.48	NA	beta-1,4-galactosyltransferase 6	protein coding
ENSG00000165813	CCDC186	10	-0.83	4.12E-03	0.46	0.09	0.43	0.20	coiled-coil domain containing 186	protein coding
ENSG00000134242	PTPN22	1	-0.83	0.02	-1.12	1.20E-04	0.41	NA	protein tyrosine phosphatase, non-receptor type 22	protein coding
ENSG00000061273	HDAC7	12	-0.83	0.04	-0.04	0.93	0.44	NA	histone deacetylase 7	protein coding
ENSG00000047346	FAM214A	15	-0.83	0.03	0.04	0.93	0.14	0.75	family with sequence similarity 214 member A	protein coding
ENSG00000113369	ARRDC3	5	-0.83	0.05	0.84	0.02	0.51	NA	arrestin domain containing 3	protein coding
ENSG00000198743	SLC5A3	21	-0.83	4.79E-04	-0.02	0.94	0.28	0.32	solute carrier family 5 member 3	protein coding

ENSG00000124496	TRERF1	6	-0.83	0.02	0.15	0.72	0.56	NA	transcriptional regulating factor 1	protein coding
ENSG00000148411	NACC2	9	-0.83	0.01	0.63	0.03	0.54	NA	NACC family member 2	protein coding
ENSG00000047056	WDR37	10	-0.84	0.03	0.23	0.57	0.66	0.12	WD repeat domain 37	protein coding
ENSG00000099992	TBC1D10A	22	-0.84	0.02	0.54	0.10	0.54	NA	TBC1 domain family member 10A	protein coding
ENSG00000114120	SLC25A36	3	-0.84	0.01	0.88	5.86E-04	0.66	NA	solute carrier family 25 member 36	protein coding
ENSG00000198901	PRC1	15	-0.84	0.04	-0.04	0.94	0.62	NA	protein regulator of cytokinesis 1	protein coding
ENSG00000127980	PEX1	7	-0.85	0.04	0.24	0.58	0.15	NA	peroxisomal biogenesis factor 1	protein coding
ENSG00000185730	ZNF696	8	-0.85	0.04	0.79	0.03	0.38	NA	zinc finger protein 696	protein coding
ENSG00000073060	SCARB1	12	-0.85	2.26E-03	0.27	0.35	0.51	NA	scavenger receptor class B member 1	protein coding
ENSG00000100055	CYTH4	22	-0.86	0.04	1.13	8.45E-04	0.66	0.15	cytohesin 4	protein coding
ENSG00000112195	TREML2	6	-0.86	0.04	0.37	0.37	0.76	0.11	triggering receptor expressed on myeloid cells like 2	protein coding
ENSG00000198324	FAM109A	12	-0.86	0.05	-0.38	0.38	0.35	NA	family with sequence similarity 109 member A	protein coding
ENSG00000175463	TBC1D10C	11	-0.87	0.03	-0.59	0.10	0.60	NA	TBC1 domain family member 10C	protein coding
ENSG00000133059	DSTYK	1	-0.87	0.02	0.84	0.01	0.74	NA	dual serine/threonine and tyrosine protein kinase	protein coding
ENSG00000111696	NT5DC3	12	-0.87	0.05	0.74	0.05	0.36	NA	5'-nucleotidase domain containing 3	protein coding
ENSG00000179859	AC025335,1	17	-0.88	0.03	0.59	0.11	0.34	NA		protein coding
ENSG00000147872	PLIN2	9	-0.88	2.18E-03	0.06	0.88	0.34	NA	perilipin 2	protein coding
ENSG00000144161	ZC3H8	2	-0.88	0.03	0.51	0.17	0.03	NA	zinc finger CCCH-type containing 8	protein coding
ENSG00000125648	SLC25A23	19	-0.88	0.03	-0.43	0.30	0.28	NA	solute carrier family 25 member 23	protein coding
ENSG00000099250	NRP1	10	-0.88	0.05	0.46	0.28	0.88	0.09	neuropilin 1	protein coding
ENSG00000181908	AP003774,4	11	-0.88	0.05	0.65	0.10	0.65	NA		lincRNA
ENSG00000169991	IFFO2	1	-0.89	0.04	0.88	0.01	0.84	NA	intermediate filament family orphan 2	protein coding
ENSG00000138050	THUMPD2	2	-0.89	0.02	0.59	0.08	0.49	NA	THUMP domain containing 2	protein coding
ENSG00000229417	NPM1P25	10	-0.89	0.02	0.93	4.49E-03	0.51	NA	nucleophosmin 1 pseudogene 25	processed pseudogene
ENSG00000118308	LRMP	12	-0.90	0.02	-0.09	0.85	0.39	0.35	lymphoid restricted membrane protein	protein coding
ENSG00000152133	GPATCH11	2	-0.91	0.02	0.28	0.50	0.64	0.15	G-patch domain containing 11	protein coding
ENSG00000165424	ZCCHC24	10	-0.91	0.01	0.81	0.01	0.69	NA	zinc finger CCHC-type containing 24	protein coding
ENSG00000198799	LRIG2	1	-0.92	0.05	0.53	0.22	0.21	NA	leucine rich repeats and immunoglobulin like domains 2	protein coding
ENSG00000137414	FAM8A1	6	-0.92	0.04	-0.10	0.85	0.56	NA	family with sequence similarity 8 member A1	protein coding
ENSG00000152409	JMY	5	-0.92	0.02	0.71	0.04	0.62	NA	junction mediating and regulatory protein, p53 cofactor	protein coding
ENSG00000158717	RNF166	16	-0.93	0.02	0.30	0.45	0.55	0.21	ring finger protein 166	protein coding
ENSG00000173621	LRFN4	11	-0.94	0.04	0.28	0.56	0.68	NA	leucine rich repeat and fibronectin type III domain containing 4	protein coding
ENSG00000163328	GPR155	2	-0.94	0.01	-0.01	0.99	0.49	NA	G protein-coupled receptor 155	protein coding
ENSG00000198551	ZNF627	19	-0.94	0.04	0.84	0.03	0.92	NA	zinc finger protein 627	protein coding
ENSG00000132613	MTSS1L	16	-0.94	0.02	0.16	0.74	0.46	NA	MTSS1L, I-BAR domain containing	protein coding
ENSG00000143816	WNT9A	1	-0.94	0.03	1.21	7.00E-04	0.74	NA	Wnt family member 9A	protein coding
ENSG00000128165	ADM2	22	-0.96	0.04	-0.24	0.64	0.78	NA	adrenomedullin 2	protein coding
ENSG00000118263	KLF7	2	-0.96	0.02	0.22	0.63	0.42	NA	Kruppel like factor 7	protein coding
ENSG00000176454	LPCAT4	15	-0.96	0.05	-0.51	0.27	0.24	NA	lysophosphatidylcholine acyltransferase 4	protein coding
ENSG00000175087	PDIK1L	1	-0.96	3.41E-03	0.69	0.02	0.67	NA	PDLIM1 interacting kinase 1 like	protein coding

ENSG00000198740	ZNF652	17	-0.97	0.01	1.13	3.67E-04	0.62	NA	zinc finger protein 652	protein coding
ENSG00000251022	THAP9-AS1	4	-0.98	0.01	1.16	3.22E-04	-0.12	NA	THAP9 antisense RNA 1	antisense
ENSG00000185432	METTL7A	12	-0.98	0.05	-0.35	0.50	0.68	0.22	methyltransferase like 7A	protein coding
ENSG00000131791	PRKAB2	1	-0.98	0.01	0.76	0.02	0.19	NA	protein kinase AMP-activated non-catalytic subunit beta 2	protein coding
ENSG00000131242	RAB11FIP4	17	-0.99	0.03	1.12	3.96E-03	0.76	NA	RAB11 family interacting protein 4	protein coding
ENSG00000136931	NR5A1	9	-1.00	0.02	0.57	0.17	0.76	NA	nuclear receptor subfamily 5 group A member 1	protein coding
ENSG00000114796	KLHL24	3	-1.00	0.03	1.44	1.58E-04	0.92	0.09	kelch like family member 24	protein coding
ENSG00000176903	PNMA1	14	-1.00	3.86E-03	0.72	0.02	0.61	NA	paraneoplastic Ma antigen 1	protein coding
ENSG00000180891	CUEDC1	17	-1.01	0.01	0.28	0.47	0.52	0.23	CUE domain containing 1	protein coding
ENSG00000129911	KLF16	19	-1.01	0.02	0.96	0.01	0.80	0.10	Kruppel like factor 16	protein coding
ENSG00000162929	KIAA1841	2	-1.02	0.05	0.03	0.96	0.12	NA	KIAA1841	protein coding
ENSG00000105245	NUMBL	19	-1.02	0.02	0.81	0.03	0.67	NA	NUMB like, endocytic adaptor protein	protein coding
ENSG00000124813	RUNX2	6	-1.02	0.01	0.58	0.09	0.49	0.25	runt related transcription factor 2	protein coding
ENSG00000101298	SNPH	20	-1.02	0.05	0.39	0.45	0.90	NA	syntaphilin	protein coding
ENSG00000198105	ZNF248	10	-1.02	0.02	0.16	0.74	0.33	NA	zinc finger protein 248	protein coding
ENSG00000164938	TP53INP1	8	-1.02	0.02	0.54	0.21	0.65	NA	tumor protein p53 inducible nuclear protein 1	protein coding
ENSG00000178695	KCTD12	13	-1.04	2.34E-03	0.63	0.05	0.96	0.02	potassium channel tetramerization domain containing 12	protein coding
ENSG00000157404	KIT	4	-1.05	0.03	-0.03	0.97	0.93	NA	KIT proto-oncogene receptor tyrosine kinase	protein coding
ENSG00000101751	POLI	18	-1.06	0.03	0.20	0.72	-0.14	NA	DNA polymerase iota	protein coding
ENSG00000133056	PIK3C2B	1	-1.06	0.02	-0.16	0.77	0.62	NA	phosphatidylinositol-4-phosphate 3-kinase catalytic subunit type 2 beta	protein coding
ENSG00000180539	C9orf139	9	-1.07	0.04	-1.25	0.01	-0.07	NA	chromosome 9 open reading frame 139	antisense
ENSG00000152804	HHEX	10	-1.07	0.03	0.95	0.03	0.88	0.12	hematopoietically expressed homeobox	protein coding
ENSG00000120784	ZFP30	19	-1.07	0.01	0.62	0.12	0.35	NA	ZFP30 zinc finger protein	protein coding
ENSG00000197299	BLM	15	-1.08	0.04	-0.11	0.87	0.81	NA	Bloom syndrome RecQ like helicase	protein coding
ENSG00000146674	IGFBP3	7	-1.09	0.03	0.56	0.23	0.92	0.10	insulin like growth factor binding protein 3	protein coding
ENSG00000036530	CYP46A1	14	-1.09	0.02	1.23	1.40E-03	0.05	NA	cytochrome P450 family 46 subfamily A member 1	protein coding
ENSG00000152518	ZFP36L2	2	-1.10	0.03	0.81	0.07	0.93	0.11	ZFP36 ring finger protein like 2	protein coding
ENSG00000132359	RAP1GAP2	17	-1.10	2.36E-03	0.44	0.23	0.38	0.36	RAP1 GTPase activating protein 2	protein coding
ENSG00000165300	SLITRK5	13	-1.10	0.03	0.17	0.77	0.97	NA	SLIT and NTRK like family member 5	protein coding
ENSG00000185112	FAM43A	3	-1.11	8.79E-05	0.24	0.45	0.70	NA	family with sequence similarity 43 member A	protein coding
ENSG00000128274	A4GALT	22	-1.12	0.01	1.12	2.02E-03	1.08	NA	alpha 1,4-galactosyltransferase	protein coding
ENSG00000155926	SLA	8	-1.12	0.04	-0.03	0.96	0.78	NA	Src like adaptor	protein coding
ENSG00000175197	DDIT3	12	-1.15	0.03	0.52	0.31	0.68	NA	DNA damage inducible transcript 3	protein coding
ENSG00000007968	E2F2	1	-1.16	0.04	0.49	0.38	0.93	0.14	E2F transcription factor 2	protein coding
ENSG00000162373	BEND5	1	-1.17	0.03	0.48	0.38	0.63	NA	BEN domain containing 5	protein coding
ENSG00000134531	EMP1	12	-1.17	0.03	-0.58	0.29	-0.02	NA	epithelial membrane protein 1	protein coding
ENSG00000175155	YPEL2	17	-1.17	1.49E-03	0.74	0.03	0.74	NA	yippee like 2	protein coding
ENSG00000255389	C6orf3	6	-1.17	0.03	0.63	0.21	0.71	NA		antisense
ENSG00000183496	MEX3B	15	-1.18	0.03	-0.85	0.08	0.46	NA	mex-3 RNA binding family member B	protein coding
ENSG00000177706	FAM20C	7	-1.18	0.05	0.44	0.48	0.97	NA	FAM20C, golgi associated secretory pathway kinase	protein coding
ENSG00000241852	C8orf58	8	-1.19	0.02	-0.62	0.23	0.44	NA	chromosome 8 open reading frame 58	protein coding
ENSG00000105327	BBC3	19	-1.21	0.05	0.66	0.25	0.89	NA	BCL2 binding component 3	protein coding

ENSG00000131149	GSE1	16	-1.23	0.01	0.97	0.02	0.88	NA	Gse1 coiled-coil protein	protein coding
ENSG00000163823	CCR1	3	-1.24	0.03	-0.15	0.83	1.02	NA	C-C motif chemokine receptor 1	protein coding
ENSG00000126351	THRA	17	-1.25	0.03	0.96	0.06	0.70	NA	thyroid hormone receptor, alpha	protein coding
ENSG00000127528	KLF2	19	-1.25	0.03	0.04	0.96	0.27	NA	Kruppel like factor 2	protein coding
ENSG00000213626	LBH	2	-1.26	2.20E-03	-0.35	0.43	0.88	NA	limb bud and heart development	protein coding
ENSG00000154856	APCDD1	18	-1.28	0.03	0.32	0.62	1.23	NA	APC down-regulated 1	protein coding
ENSG00000198948	MFAP3L	4	-1.28	0.04	-0.50	0.45	0.67	NA	microfibrillar associated protein 3 like	protein coding
ENSG00000103066	PLA2G15	16	-1.28	0.02	1.41	2.05E-03	1.19	0.07	phospholipase A2 group XV	protein coding
ENSG00000139318	DUSP6	12	-1.29	4.81E-04	-0.19	0.68	-0.12	NA	dual specificity phosphatase 6	protein coding
ENSG00000135750	KCNK1	1	-1.30	0.01	1.16	0.01	0.91	NA	potassium two pore domain channel subfamily K member 1	protein coding
ENSG00000276043	UHRF1	19	-1.31	0.02	-0.41	0.47	0.76	NA	ubiquitin like with PHD and ring finger domains 1	protein coding
ENSG00000213965	NUDT19	19	-1.33	0.02	1.94	1.93E-05	1.55	NA	nudix hydrolase 19	protein coding
ENSG00000277048	NA	NA	-1.34	0.02	0.30	0.62	0.29	NA	NA	NA
ENSG00000279496	NA	NA	-1.34	0.01	1.49	7.54E-04	0.94	NA	NA	NA
ENSG00000156804	FBXO32	8	-1.34	0.02	0.72	0.20	0.85	NA	F-box protein 32	protein coding
ENSG00000185669	SNAI3	16	-1.35	0.04	0.46	0.49	1.07	NA	snail family transcriptional repressor 3	protein coding
ENSG00000116852	KIF21B	1	-1.35	2.09E-04	-0.42	0.29	0.62	NA	kinesin family member 21B	protein coding
ENSG00000123384	LRP1	12	-1.37	0.02	-0.76	0.17	-0.47	NA	LDL receptor related protein 1	protein coding
ENSG00000067191	CACNB1	17	-1.37	0.01	0.07	0.92	0.08	NA	calcium voltage-gated channel auxiliary subunit beta 1	protein coding
ENSG00000113758	DBN1	5	-1.37	0.03	0.82	0.17	1.13	NA	drebrin 1	protein coding
ENSG00000069702	TGFBR3	1	-1.40	0.01	0.83	0.12	1.11	NA	transforming growth factor beta receptor 3	protein coding
ENSG00000196220	SRGAP3	3	-1.42	0.05	1.29	0.04	0.70	NA	SLIT-ROBO Rho GTPase activating protein 3	protein coding
ENSG00000109881	CCDC34	11	-1.42	0.02	0.34	0.62	1.14	NA	coiled-coil domain containing 34	protein coding
ENSG00000090447	TFAP4	16	-1.43	0.01	1.09	0.03	1.14	NA	transcription factor AP-4	protein coding
ENSG00000168010	ATG16L2	11	-1.44	0.05	0.36	0.65	-0.57	NA	autophagy related 16 like 2	protein coding
ENSG00000101695	RNF125	18	-1.46	0.03	0.05	0.95	0.90	NA	ring finger protein 125	protein coding
ENSG00000166825	ANPEP	15	-1.47	0.02	-0.17	0.83	0.17	NA	alanyl aminopeptidase, membrane	protein coding
ENSG00000198835	GJC2	1	-1.47	0.01	0.65	0.21	0.98	NA	gap junction protein gamma 2	protein coding
ENSG00000124882	EREG	4	-1.50	0.04	-1.02	0.13	-1.11	NA	epiregulin	protein coding
ENSG00000102760	RGCC	13	-1.51	0.03	-0.75	0.28	0.37	NA	regulator of cell cycle	protein coding
ENSG00000134955	SLC37A2	11	-1.54	1.52E-03	-0.41	0.47	0.84	NA	solute carrier family 37 member 2	protein coding
ENSG00000004399	PLXND1	3	-1.54	3.11E-03	-0.21	0.73	0.89	NA	plexin D1	protein coding
ENSG00000178732	GP5	3	-1.58	3.11E-03	-0.18	0.79	1.23	NA	glycoprotein V platelet	protein coding
ENSG00000085265	FCN1	9	-1.63	0.04	1.14	0.11	0.36	NA	ficolin 1	protein coding
ENSG00000205336	ADGRG1	16	-1.63	0.02	0.04	0.96	0.45	NA	adhesion G protein-coupled receptor G1	protein coding
ENSG00000099953	MMP11	22	-1.66	0.01	0.93	0.11	1.41	NA	matrix metallopeptidase 11	protein coding
ENSG00000236717	RP11-100G15,10	9	-1.69	0.03	-0.35	0.69	1.03	NA		unprocessed pseudogene
ENSG00000138061	CYP1B1	2	-1.75	1.45E-03	-1.18	0.02	0.35	NA	cytochrome P450 family 1 subfamily B member 1	protein coding
ENSG00000187474	FPR3	19	-1.76	0.01	0.90	0.15	0.67	NA	formyl peptide receptor 3	protein coding
ENSG00000091972	CD200	3	-1.76	0.01	0.99	0.14	1.07	NA	CD200 molecule	protein coding
ENSG00000202198	7SK	6	-1.80	0.02	-0.34	0.69	0.02	NA	7SK RNA	misc RNA

ENSG00000108551	RASD1	17	-1.90	1.41E-05	0.94	0.02	1.38	NA	ras related dexamethasone induced 1	protein coding
ENSG00000258227	CLEC5A	7	-2.60	8.85E-05	0.57	0.45	0.73	NA	C-type lectin domain family 5 member A	protein coding
ENSG00000137801	THBS1	15	-2.77	4.71E-07	-1.44	0.01	-0.16	NA	thrombospondin 1	protein coding
ENSG00000170458	CD14	5	-2.79	3.21E-06	1.40	0.02	0.51	NA	CD14 molecule	protein coding
ENSG00000038427	VCAN	5	-3.66	3.73E-11	-0.47	0.51	-0.01	NA	versican	protein coding
