# Supplementary material for: Cytokine production by activated plasmacytoid dendritic cells and natural killer cells is suppressed by an IRAK4 inhibitor
Source: Arthritis Res Ther. 2018 Oct 24;20:238. doi: 10.1186/s13075-018-1702-0 (PMC6235225; doi:10.1186/s13075-018-1702-0)
Supplement: Supplementary file 8 — Table S3. Upstream regulators. (PDF 291 kb) [file 13075_2018_1702_MOESM8_ESM.pdf]

Additional file 8. Upstream regulators

Table S3. Significantly enriched upstream regulators* for RNAIC vs MOCK stimulated pDCs based on all differentially expressed genes (FDR<0.05) with log2FC>1 (n=339). *Pathway Studio ** One-sided Mann-Whitney U-test

Upstream regulator	Total number of downstream genes (n)	Overlap (n)	Overlap (%)	Overlapping Entities	p-value**	Jaccard similarity
IRF1	127	34	26	CXCL9;CXCL10;TNF;CD83;IRF2;IRF1;SOCS1;DDX58;TP53;TAP1;ICAM1;TAPBP;PMAIP1;CYBB;PSMB9;BBC3;HLA-A;IFIT1;NFKBIE;IFIT3;TNFSF10;PTGS2;IFNB1;BIRC3;TLR7;CD274;CDKN1A;OPTN;THBS1;GBP2;GBP1;CXCL8;MYD88;IL12A	2.80E-13	0.05
STAT1	205	42	20	HSPA1A;TNF;HSP90AB1;SOCS1;RUNX2;MVP;ICAM1;PML;CYBB;TRIP6;CD14;IFIT2;SOD2;SMAD3;TNFSF10;CD40;SCARB1;IFNB1;FAS;MCL1;CDKN1A;CXCL2;CXCL8;IL12A;CXCL9;CXCL10;IRF1;SOCS3;TAP1;BCL2L1;BCL2A1;PSMB9;ULK1;TYMP;PTGS2;TLR7;CD274;ADAR;GBP2;GBP1;MYD88;SLC1A3	6.97E-12	0.05
RELB	62	20	31	IFNB1;PTGS2;BIRC3;TNF;TLR7;RELA;RELB;TP53;BECN1;CXCL2;PSMA5;TRAF2;BCL2L1;BCL2A1;HIF1A;CXCL8;GADD45B;IL12A;NFKBIA;SOD2	7.58E-10	0.03
NFKB1	86	23	26	CXCL9;PTGS2;IFNB1;BIRC3;CXCL10;FAS;TNFAIP3;TNF;MCL1;OPTN;BCL2L1;BCL2A1;CREB1;BCL3;CD82;FSCN1;CXCL8;HIF1A;IL12A;ATF3;NFKB1;NFKB2;SOD2	2.32E-09	0.03
RELA	252	42	16	TADA2B;HSPA1A;TNFAIP3;TNF;RUNX2;RELA;RELB;TP53;BECN1;ICAM1;PEA15;TRAF1;LTB;NFKBIA;NFKBIE;NFKB1;NFKB2;CD40;IFNB1;BIRC3;FAS;NOTCH2;MCL1;CDKN1A;CXCL2;CXCL8;IL12A;CXCL10;IRF1;DDX58;BCL2L1;BCL2A1;BCL3;HIF1A;HLA-A;CCL4;PTGS2;SDC4;CD274;ACTN4;CREB1;GADD45B	5.64E-09	0.05
SP1	1358	130	9	EREG;TNFAIP3;TNFAIP2;TNF;NBN;MFN2;TP53;TPI1;MVP;HDAC7;NFKBIA;NFKB1;TSG101;TXN;NPPC;FLNA;NAMPT;MMP11;MRC1;DUSP6;TYMP;MTA3;TGFBR3;THBS1;SLC29A1;THRA;PIM1;PLAT;PMAIP1;ANPEP;BBC3;SLC39A1;FAS;PPA1;PPARA;CXCL2;GTF2B;EZR;TINF2;WNT9A;CYCS;IFIH1;XRCC5;NR5A1;AATF;TNIP1;ABCF1;CKAP2;ACHE;ACTA2;KLF2;PRMT5;PLIN2;GCH1;PGK1;HSPA1A;TP63;CASP3;HSPA8;HSP90AB1;RUNX2;RELA;RUNX3;REST;BECN1;ICAM1;CCND2;ICAM2;CD14;IFI16;TNFSF9;TNFSF10;CD40;SCARB1;IGFBP3;PARK7;CDH1;NRP1;CDKN1A;CFLAR;CXCL8;IL12A;ATP1A1;NEU3;BCL2L1;HHEX;HIF1A;CENPW;PTGER4;PTGS2;NECTIN2;CA2;VCAN;LGALS1;SLC12A2;CD83;CSRP2;B4GALT5;CTNS;SLC9A3R2;CTNND1;LRP1;LTB;LTA;FSCN1;CYP1B1;CYLD;IL21R;SMAD3;SOD2;SP3;CD55;DBI;DDB1;SRC;SLC4A7;MCL1;DDIT3;IRF2;IRF1;SOCS3;RGCC;CD82;CCR7;CD274;KIT;CREB1;CREM;PTTG1;SLC1A3	3.05E-07	0.07
IRF3	53	15	27	IFNA2;TNFSF10;IFNB1;CXCL10;TNF;ZC3HAV1;TP53;CDKN1A;ICAM1;PMAIP1;CXCL8;IL12A;IFIT1;NFKBIE;IFIT2	7.23E-07	0.02
STAT3	418	52	12	HSPA1A;CASP3;HSP90AB1;SOCS1;RUNX2;RELA;TP53;ICAM1;CCND2;EED;CD14;IFIT1;NFKB1;CDH1;IL18R1;CDKN1A;IL2RG;NAMPT;CXCL8;IL6ST;IL12A;BCL2L1;PSMB5;BCL3;PSMB8;HIF1A;PSMB9;ECE1;PTGS2;THBS1;MYD88;DNAJB4;PIM1;PML;FSCN1;SOD2;NR4A3;FAS;MCL1;DDIT3;CXCL2;STAT3;ATF3;IRF1;SOCS3;JUNB;KLF2;CD274;PRMT5;CREM;PTTG1;SLC1A3	1.69E-06	0.05
JUN	421	52	12	HSPA1A;TP63;CASP3;TNF;RUNX2;TP53;RELB;REST;BECN1;ICAM1;PEA15;CCND2;POLI;NINJ1;IFNB1;TXN;CDKN1A;CFLAR;CXCL8;MMP11;SESN2;BCL2L1;BCL2A1;HIF1A;PPP1R15A;HK1;PTGS2;NECTIN2;THBS1;SLC29A1;TGIF1;GLUL;BBC3;SMAD3;BIRC3;SP3;FAS;PPARA;DDIT3;CXCL2;SMURF1;ATF3;CHKA;EZR;SOCS3;WNT9A;CD82;NR5A1;CCL4;CITED2;DDX21;CREB1	2.10E-06	0.05
FOXL2	21	9	40	FAS;CDKN1A;PTTG1;ACTA2;TNFRSF1A;CCND2;NR5A1;SOD2;SMAD3	3.52E-06	0.01
STAT2	27	10	35	TAP1;ADAR;SCARB1;GBP1;MYD88;IRF1;IFIT3;NFKB2;IFIT2;MCL1	4.30E-06	0.02
ATF2	104	20	19	CASP3;TNF;CSRP2;PLAT;RELA;TP53;CBFB;ICAM1;HIF1A;PPP1R15A;HK1;SMAD3;PTGS2;IFNB1;ACHE;DDIT3;CDKN1A;CREB1;CXCL8;ATF3	7.22E-06	0.03
CYLD	18	8	42	BCL3;STAT3;TNFRSF1A;MYD88;RIPK2;CYLD;DDX58;TRAF2	9.70E-06	0.01
CEBPB	366	45	12	TNF;HSP90AB1;TNFRSF1A;PLAT;RUNX2;RELA;TP53;ICAM1;CD14;NFKBIB;NFKB1;SOD2;SCARB1;FAS;IGFBP3;PPARA;ANP32A;CDH1;DDIT3;CDKN1A;BANP;CXCL8;STAT3;IL12A;ATF3;CXCL9;CXCL10;CHKA;MMP11;FBXO32;CD200;SOCS3;HCLS1;SESN2;RGCC;CCR7;CCL3;CCL4;PTGER4;PTGS2;FOXA1;ACTA2;PLIN2;GCH1;GADD45B	1.18E-05	0.05
PSME3	6	5	71	PSME3;PSME1;SMURF1;CDKN1A;KLF2	1.85E-05	0.01
HIF1A	328	41	12	ATRIP;HSPA1A;LGALS1;CASP3;TNF;NBN;RELA;EIF3I;BECN1;PMAIP1;LRP1;CYBB;SOD2;SMAD3;NR4A3;CD55;IGFBP3;PPARA;CDH1;MCL1;JMY;DDIT3;CDKN1A;NAMPT;CXCL8;CHKA;P4HA2;P4HA1;CYP46A1;BCL2L1;TCEB1;CD82;HIF1A;ECE1;PTGS2;ACTA2;CITED2;CD274;PIM2;SLC29A1;PGK1	1.98E-05	0.05
KLF6	53	13	24	PPARA;CDH1;TP53;SOCS3;CDKN1A;PMAIP1;CXCL2;COX5A;CXCL8;IL6ST;PTTG1;NFKBIA;ATF3	2.20E-05	0.02

HSPA1A	112	20	17	PIM1;HSPA1A;TNF;IRF1;RELA;TP53;TAP1;BECN1;CYCS;CYBB;HIF1A;CD14;NFKBIA;APAF1;SOD2;SMAD3;CD40;MCL1;APOBEC3G;ENDOG	2.25E-05	0.03
TRAF6	47	12	25	CD40;RIPK2;BIRC3;TRAF2;TGFBR3;DCP1A;MAP3K1;MAP3K2;HIF1A;STAT3;SRC;SMAD3	3.03E-05	0.02
BCL2	34	10	28	BECN1;AMBRA1;XRCC6;COX5A;HSPA8;XRCC5;TNFRSF1A;APAF1;TP53;MCL1	4.02E-05	0.02
STIP1	7	5	62	CDC37;STIP1;HSPA8;STAT3;HSPA1A	4.68E-05	0.01
NFKB2	22	8	34	CREB1;FAS;CXCL9;HLA-A;PTGS2;RELA;RELB;NFKB2	5.02E-05	0.01
AATF	17	7	38	CDKN1A;BBC3;CASP10;TSG101;PEA15;AATF;TP53	6.61E-05	0.01
ATF4	85	16	18	PTGS2;RUNX2;TP53;BECN1;DDIT3;ATF5;PMAIP1;CREB1;GCH1;HIF1A;STAT3;HERPUD1;BBC3;PPP1R15A;ULK1;ATF3	7.91E-05	0.02
IRF2	30	9	29	TNFSF10;IFNB1;TAPBP;CYBB;CD83;TLR7;IRF2;NFKBIE;RELA	8.54E-05	0.01
KLF4	174	25	14	TNF;HSP90AB1;SOCS1;MFN2;RUNX2;RELA;TP53;RUNX3;ICAM1;CCND2;CD14;ULK1;SMAD3;ABCF1;SCARB1;PTGS2;IFNB1;ACTA2;KIT;CDH1;SLC4A7;KPNA2;CDKN1A;CXCL8;ATF3	9.73E-05	0.03
TRAF3	19	7	35	CREB1;TNFRSF1A;ADAP2;TANK;SRC;TRAF2;MAP3K1	1.44E-04	0.01
SPI1	200	27	13	TNF;RUNX3;CTSS;CCND2;CYBB;CD14;IFIT3;TNFSF10;CD40;IFNB1;MCL1;IL12A;CXCL9;IRF1;CLEC5A;MRC1;BCL2L1;PSMB8;PSMB9;CCL3;HLA-DQB1;PTGS2;CITED2;KIT;BLNK;ADAR;DRAM1	1.50E-04	0.03
COMMD1	14	6	40	SLC12A2;COMMD1;HIF1A;SOCS1;HSPA1A;RELA	1.88E-04	0.01
DNAJA3	9	5	50	HSPA8;NFKBIA;STAT3;HSPA1A;TP53	1.89E-04	0.01
PMAIP1	5	4	66	PMAIP1;BCL2L1;BCL2A1;MCL1	2.12E-04	0.01
TAPBP	5	4	66	TAPBP;HLA-B;TAP1;HLA-A	2.12E-04	0.01
PSMB8	2	3	100	POMP;PSMB8;PSMB9	2.50E-04	4.82E-03
PIAS1	58	12	20	CXCL9;CXCL10;STIP1;SP3;TNF;RELA;TP53;CDKN1A;CXCL2;STAT3;NR5A1;NFKBIA	2.57E-04	0.02
GTF2B	35	9	25	CDKN1A;CFLAR;CCND2;SP3;HSPA1A;CREM;GTF2B;RELA;SOD2	3.00E-04	0.01
CTNNB1	242	30	12	VCAN;RUNX2;TP53;CCND2;PML;FSCN1;DKK4;IFIT1;IFNB1;GLCE;FAS;CDH1;NOTCH2;MCL1;NPPC;CXCL8;STAT3;DUSP6;BCL2L1;HLA-A;NR5A1;CCL4;PTGS2;SMC3;ACTA2;KLF2;CREB1;TGIF1;XIRP1;PTTG1	3.01E-04	0.04
REL	105	17	16	CXCL10;TNF;CD83;ICAM1;BCL2L1;HIF1A;NFKBIA;IFI16;IFIT3;SOD2;TNFSF10;IFNB1;CPSF3;MCL1;GBP2;CXCL8;IL12A	3.16E-04	0.02
FOS	188	25	13	TNF;TNFRSF1A;MMP11;RELA;RELB;TP53;CBFB;SOCS3;PRDX1;ICAM1;BCL2L1;CD82;NR5A1;PTGS2;SP3;ACHE;FAS;FOXA1;CDH1;DDIT3;CDKN1A;CFLAR;CXCL2;ENO1;CXCL8	3.32E-04	0.03
RBPJ	78	14	17	PTGS2;CASP3;ACTA2;NOTCH2;TP53;MCL1;SOCS3;CDKN1A;CTNND1;BCL2A1;HERPUD1;NFKBIA;NFKB1;NFKB2	3.70E-04	0.02
BCL2L1	22	7	30	BID;BECN1;MFN2;CYCS;APAF1;BCL2L1;TP53	3.86E-04	0.01
IRF9	16	6	35	GBP1;MYD88;IRF1;IFNB1;IFIT3;IFIT2	4.17E-04	0.01
TRAF1	6	4	57	BIRC3;TRAF2;TRAF1;TNFRSF1A	4.70E-04	0.01
MCM7	6	4	57	CEP68;ATRIP;TCEB1;HIF1A	4.70E-04	0.01
NFKBIB	6	4	57	NFKBIB;TNF;RELA;NFKB1	4.70E-04	0.01
BCL6	62	12	19	CD69;SOCS1;CDH1;CISH;TP53;SOCS3;ADAR;BAG6;CDKN1A;CCND2;BCL2L1;CCL3	4.86E-04	0.02
HSPB1	38	9	23	BID;CYCS;FAS;CASP3;STAT3;ACTA2;NFKBIA;APAF1;TP53	5.70E-04	0.01
CREB1	587	57	9	HSPA1A;TNF;HSP90AB1;TP53;REST;RFX5;ICAM1;CCND2;CD14;NFKBIA;CD40;SCARB1;IFNB1;CD69;CPSF3;IGFBP4;CXCL8;GYS1;CHCHD2;BCL2L1;PPP1R15A;HLA-A;HLA-B;PTGER4;PTGS2;HLA-E;FOXA1;NECTIN2;THBS1;PLAT;COX5A;FSCN1;CYP1B1;BBC3;SOD2;SMAD3;NR4A3;SLC39A1;CD55;MCL1;CXCL2;STAT3;GTF2B;ATF3;CXCL10;CYCS;JUNB;CCR7;CCL4;SMC3;ACHE;KPNA2;CREB1;GCH1;CREM;PGK1;SKP1	6.75E-04	0.05
TCF7L2	84	14	16	PTGS2;GLCE;SMC3;VCAN;HECA;CDH1;TP53;NUP62;TGIF1;FSCN1;STAT3;IL6ST;PTTG1;DKK4	7.97E-04	0.02
DDIT3	48	10	20	PTGS2;PPARA;IRF1;RELA;DDIT3;CDKN1A;ATF5;CXCL8;BBC3;PPP1R15A	8.07E-04	0.02
NFKBIA	18	6	31	TNF;CXCL8;NFKBIA;NFKBIE;NFKB1;NFKB2	8.19E-04	0.01
HIVEP1	7	4	50	IFNB1;IRF1;CDKN1A;TP53	8.93E-04	0.01
TRAF5	7	4	50	TRAF2;TRAF1;IL6ST;MAP3K1	8.93E-04	0.01
RNF31	7	4	50	CYLD;CD40;DDX58;TNFRSF1A	8.93E-04	0.01
ATG14	3	3	75	SNAP29;ATG14;SNAPIN	9.51E-04	4.81E-03
DSG3	3	3	75	JUP;EZR;SRC	9.51E-04	4.81E-03
DNAJA1	3	3	75	DNAJA1;HSPA8;HSPA1A	9.51E-04	4.81E-03
ICAM2	3	3	75	EZR;ICAM2;ICAM1	9.51E-04	4.81E-03
TNFRSF9	3	3	75	TRAF2;TRAF1;TNFRSF4	9.51E-04	4.81E-03
EIF3I	3	3	75	GCH1;EZR;EIF3I	9.51E-04	4.81E-03
ZBTB2	3	3	75	ZBTB2;CDKN1A;TP53	9.51E-04	4.81E-03
NRBF2	3	3	75	NRBF2;BECN1;ATG14	9.51E-04	4.81E-03
DNMT3A	67	12	17	APCDD1;PTGS2;FAS;TNF;CITED2;CDH1;TP53BP2;CDKN1A;IL2RG;TP53INP1;HK1;TNFSF9	9.94E-04	0.02

AR	180	23	12	VCAN;TNF;RUNX2;RELA;TP53;B2M;BCL2L1;NR5A1;IFI16;PTGS2;IGFBP3;PPARA;SNAI3;KLF2;KIT;CDH1;SRC;KPNA2;CDKN1A;CFLAR;CREB1;CXCL8;PTTG1	9.96E-04	0.03
PARK2	19	6	30	BECN1;AMBRA1;PARK7;OPTN;TRAF2;HSPA1A	1.11E-03	0.01
TBP	97	15	15	HSPA1A;LGALS1;TNF;RELA;ICAM1;SUPT16H;CYP1B1;PTGS2;IFNB1;IGFBP3;TXN;CDKN1A;CXCL2;CXCL8;GTF2B	1.15E-03	0.02
HSP90AB1	13	5	35	HIF1A;HSP90AB1;LRP1;APAF1;HSPA1A	1.22E-03	0.01
CIITA	13	5	35	PSMC5;HLA-DRB1;HLA-A;BTN2A2;HLA-DQA1	1.22E-03	0.01
BCL3	27	7	25	CRTC3;BIRC3;BCL3;TNF;CXCL8;NFKB1;NFKB2	1.41E-03	0.01
IRF5	20	6	28	TNFSF10;TNF;CD83;CDKN1A;IFNB1;IRF1	1.47E-03	0.01
FBXW7	20	6	28	KLF2;ENO1;NFE2L3;RUNX2;NFKB2;MCL1	1.47E-03	0.01
BBC3	8	4	44	BCL2L1;BBC3;BCL2A1;MCL1	1.53E-03	0.01
PIN1	90	14	15	NR4A3;PIM1;TP63;RUNX2;TP53;MCL1;RUNX3;APOBEC3G;PML;CREB1;PGK1;HIF1A;AATF;SMAD3	1.58E-03	0.02
SP3	293	32	10	TNF;PLAT;TP53;REST;SLC9A3R2;MVP;LRP1;IL21R;TNFSF9;SOD2;SMAD3;TNFSF10;CD40;SCARB1;SP3;IGFBP3;SLC4A7;NPPC;CDKN1A;ATF3;NEU3;CYP46A1;SOCS3;RGCC;HHEX;NR5A1;CENPW;TNIP1;PTGS2;GCH1;PGK1;PTTG1	1.71E-03	0.04
TAF1	14	5	33	FAS;CDKN1A;IFNB1;SRC;HSPA1A	1.73E-03	0.01
COPS8	14	5	33	MAPRE1;SKP1;DDB1;NFKBIA;TP53	1.73E-03	0.01
MUC1	21	6	27	JUP;HIF1A;CTNND1;ICAM1;RELA;TP53	1.92E-03	0.01
HDAC6	21	6	27	HIF1A;STAT3;CDKN1A;MYD88;MFN2;TP53	1.92E-03	0.01
HSF1	102	15	14	DNAJA1;HSPA1A;CDC37;TNF;HSPA8;HSP90AB1;SOCS3;HSPBP1;NFKBIA;SMAD3;PTGS2;STIP1;UBB;CXCL8;STAT3	1.92E-03	0.02
FOXO3	124	17	13	FBXO32;SESN3;GLUL;SOCS3;CCND2;BBC3;TANK;APAF1;SMAD3;SOD2;TNFSF10;IGFBP3;CITED2;TXN;CDKN1A;CFLAR;NAMPT	2.12E-03	0.02
SUMO1	29	7	23	PML;FAS;HIF1A;TNFRSF1A;IRF1;TP53;SMAD3	2.18E-03	0.01
PTGS2	4	3	60	PTGS2;CYCS;TP53	2.27E-03	4.80E-03
ARID3A	4	3	60	CDKN1A;PML;TP53	2.27E-03	4.80E-03
ATP1A1	4	3	60	PRKCH;ATP1A1;SRC	2.27E-03	4.80E-03
TAP1	4	3	60	TAP1;B2M;HLA-A	2.27E-03	4.80E-03
IFIT1	4	3	60	IFIT1;IFIT3;IFIT2	2.27E-03	4.80E-03
HSPBP1	4	3	60	HSPBP1;HSPA8;HSPA1A	2.27E-03	4.80E-03
SSRP1	4	3	60	SSRP1;SUPT16H;TP53	2.27E-03	4.80E-03
GATAD2B	4	3	60	PTGS2;GATAD2B;CXCL8	2.27E-03	4.80E-03
IRF8	46	9	19	APOBEC3G;CXCL9;IFNB1;PTGS2;CYBB;TNF;IRF1;IL12A;IFIT1	2.33E-03	0.01
BAG3	15	5	31	HSPA8;MVP;BCL2L1;HSPA1A;MCL1	2.39E-03	0.01
SMAD2	94	14	14	PTGS2;CSRP2;ACTA2;MMP11;CITED2;CDH1;MFN2;BECN1;CDKN1A;RGCC;JUNB;HHEX;FSCN1;STAT3	2.40E-03	0.02
CDK9	30	7	22	CDKN1A;PTGS2;CCND2;TNF;CXCL8;CYP1B1;IL12A	2.66E-03	0.01
BTRC	47	9	18	REST;JUP;LPIN1;NFKBIB;IL10RA;NFKBIE;NFKB1;TP53;SMAD3	2.72E-03	0.01
CLU	16	5	29	XRCC6;NFKBIA;MSRB1;BCL2L1;MMP25	3.21E-03	0.01
CFLAR	16	5	29	CASP3;CASP10;CFLAR;AATF;TRAF2	3.21E-03	0.01
CCNT1	10	4	36	MRC1;TNF;RN7SK;HSPA1A	3.62E-03	0.01
HSPD1	24	6	24	HLA-E;BCL2L1;HSPA1A;CASP3;HLA-A;TP53	3.87E-03	0.01
ETS1	200	23	11	EREG;CASP3;TNF;SOCS1;RUNX2;TP53;RUNX3;ICAM1;LTB;ANPEP;PPARA;MCL1;CDKN1A;SRGN;CXCL8;PRDX1;DUSP6;BCL2L1;PIM3;JUNB;ECE1;ZNF267;PTGS2	3.94E-03	0.03
HLA-B	1	2	100	HLA-B;HLA-A	3.97E-03	3.21E-03
TYMP	1	2	100	TYMP;LYN	3.97E-03	3.21E-03
