# Supplementary material for: Cytokine production by activated plasmacytoid dendritic cells and natural killer cells is suppressed by an IRAK4 inhibitor
Source: Arthritis Res Ther. 2018 Oct 24;20:238. doi: 10.1186/s13075-018-1702-0 (PMC6235225; doi:10.1186/s13075-018-1702-0)
Supplement: Supplementary file 9 — Figure S6. Overlap of differentially expressed genes in plasmacytoid dendritic cells. (PDF 135 kb) [file 13075_2018_1702_MOESM9_ESM.pdf]

Additional file 9. Overlap of differentially expressed genes in plasmacytoid dendritic cells

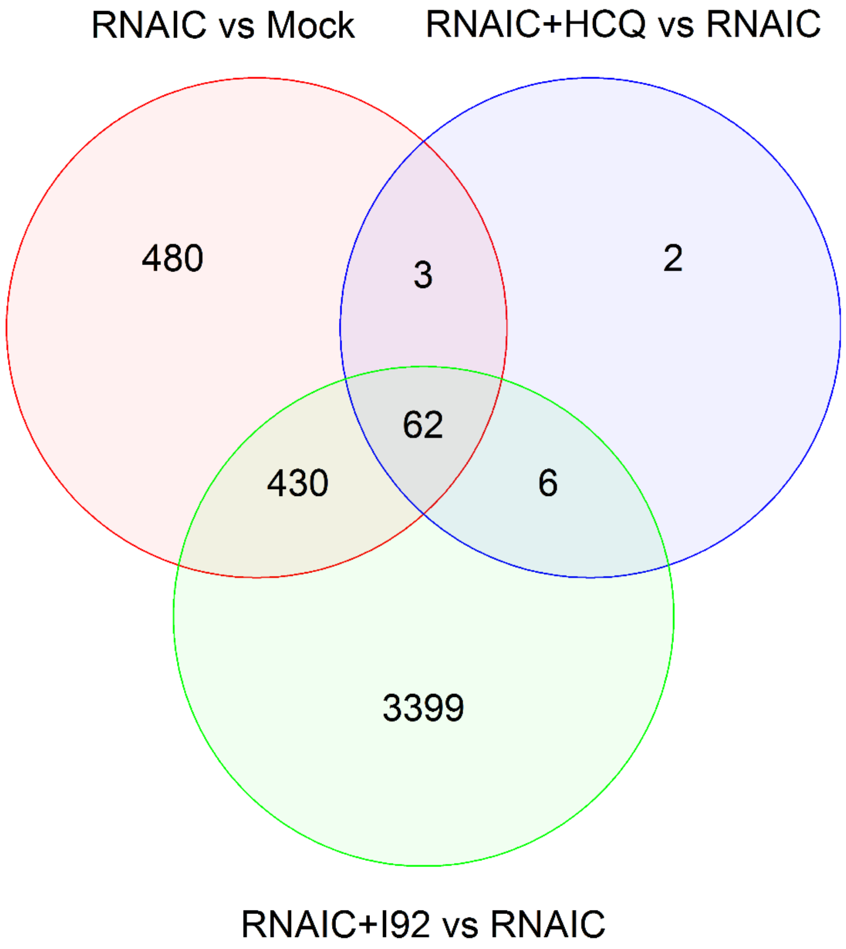

Additional figure S6. Venn diagram showing number of overlapping differentially expressed genes (DEGs) between the different plasmacytoid dendritic cell (pDC) treatment conditions. (FDR<0.05) Red: 975 DEGs in RNA-containing immune complexes (RNA-IC) vs mock stimulated pDCs. Blue: 73 DEGs in hydroxychloroquine (HCQ) treated, RNA-IC stimulated vs RNA-IC stimulated pDCs. Green: 3897 DEGs in the IRAK-4 inhibitor (I92) treated, RNA-IC stimulated vs RNA-IC stimulated pDCs.
