# Supplementary material for: Cytokine production by activated plasmacytoid dendritic cells and natural killer cells is suppressed by an IRAK4 inhibitor
Source: Arthritis Res Ther. 2018 Oct 24;20:238. doi: 10.1186/s13075-018-1702-0 (PMC6235225; doi:10.1186/s13075-018-1702-0)
Supplement: Supplementary file 10 — Table S4. Enriched biological function pathways. (PDF 249 kb) [file 13075_2018_1702_MOESM10_ESM.pdf]

Additional file 10. Enriched biological function pathways

Table S4. Significantly enriched biological function pathways* based on differentially expressed genes (FDR<0.05) for RNAIC vs MOCK stimulated pDCs and for RNAIC HCQ vs RNAIC I92 (n=125) * total number of entitites/genes annotated within pathway. *Pathway Studio
** One-sided Mann-Whitney U-test

**Biological function**	**Entities (n)***	**Expanded entities (n)**	**Overlapping entities (n)**	**Overlap (%)**	**Name of overlapping entities**	**p-value****	**Jaccard similarity**
Macroautophagy Decline	35	42	3	7	ATG14;AMBRA1;BECN1	5.16E-04	0.02
Metaphase/Anaphase Phase Transition	16	62	3	4	PSMB8;PSMB9;PTTG1	1.61E-03	0.02
Endogenous Peptide Antigen Presentation	16	46	2	4	PSMB8;PSMB9	0.01	0.01
Protein Folding	24	134	3	2	CCT7;PSMB8;PSMB9	0.01	0.01
ER Associated Degradation in Vesicular Transport	26	62	2	3	PSMB8;PSMB9	0.02	0.01
mTOR Signaling Overview	112	301	4	1	AMBRA1;ATG14;PSMB8;PSMB9	0.03	0.01
Ubiquitin Dependent Protein Degradation	13	82	2	2	PSMB8;PSMB9	0.04	0.01
