# Supplementary material for: Cytokine production by activated plasmacytoid dendritic cells and natural killer cells is suppressed by an IRAK4 inhibitor
Source: Arthritis Res Ther. 2018 Oct 24;20:238. doi: 10.1186/s13075-018-1702-0 (PMC6235225; doi:10.1186/s13075-018-1702-0)
Supplement: Supplementary file 11 — Table S5. Enriched signal processing pathways. (PDF 251 kb) [file 13075_2018_1702_MOESM11_ESM.pdf]

Additional file 11. Enriched signal processing pathways

Table S5. Significantly enriched signal processing pathways* based on differentially expressed genes (FDR<0.05) for RNAIC vs MOCK stimulated pDCs and for RNAIC HCQ vs RNAIC I92 (n=125) * total number of entitites/genes annotated within pathway. *Pathway Studio
** One-sided Mann-Whitney U-test

**Signaling Target**	**Entities (n)***	**Expanded entities (n)**	**Overlapping entities (n)**	**Overlap (%)**	**Name of overlapping entities**	**p-value****	**Jaccard similarity**
PTPRJ -> CTNND Signaling	2	2	1	50	CTNND1	0.01	0.01
KIT -> STAT Signaling	6	6	1	16	KIT	0.03	0.01
CCL15 Expression Targets	7	7	1	14	CCR1	0.04	0.01
IL4R -> STAT Signaling	7	7	1	14	IL2RG	0.04	0.01
IL13R -> STAT6 Signaling	7	7	1	14	IL2RG	0.04	0.01
VEGFR -> CTNND Signaling	7	7	1	14	CTNND1	0.04	0.01
CCL7 Expression Targets	8	8	1	12	CCR1	0.04	0.01
PTPRC -> STAT6 Expression Targets	8	8	1	12	LGALS1	0.04	0.01
IL21R -> STAT Signaling	8	8	1	12	IL2RG	0.04	0.01
IL9R -> STAT Signaling	8	8	1	12	IL2RG	0.04	0.01
NF-kB Canonical Signaling	62	151	3	1	LTB;PSMB8;PSMB9	0.04	0.01
CCL8 Expression Targets	9	9	1	11	CCR1	0.046	0.01
IL3R -> STAT Signaling	9	9	1	11	IL3RA	0.046	0.01
IL7R -> STAT Signaling	9	9	1	11	IL2RG	0.046	0.01
