# Supplementary material for: Cytokine production by activated plasmacytoid dendritic cells and natural killer cells is suppressed by an IRAK4 inhibitor
Source: Arthritis Res Ther. 2018 Oct 24;20:238. doi: 10.1186/s13075-018-1702-0 (PMC6235225; doi:10.1186/s13075-018-1702-0)
Supplement: Supplementary file 12 — Figure S7. RNA-seq analysis of cytokine expression in plasmacytoid dendritic cells stimulated for 6 h in the presence of IRAK4 inhibitor or hydroxychloroquine. (PDF 186 kb) [file 13075_2018_1702_MOESM12_ESM.pdf]

Additional file 12. RNASeq analysis of cytokine expression in plasmacytoid dendritic cells stimulated for 6 h in presence of IRAK4 inhibitor or hydroxychloroquine

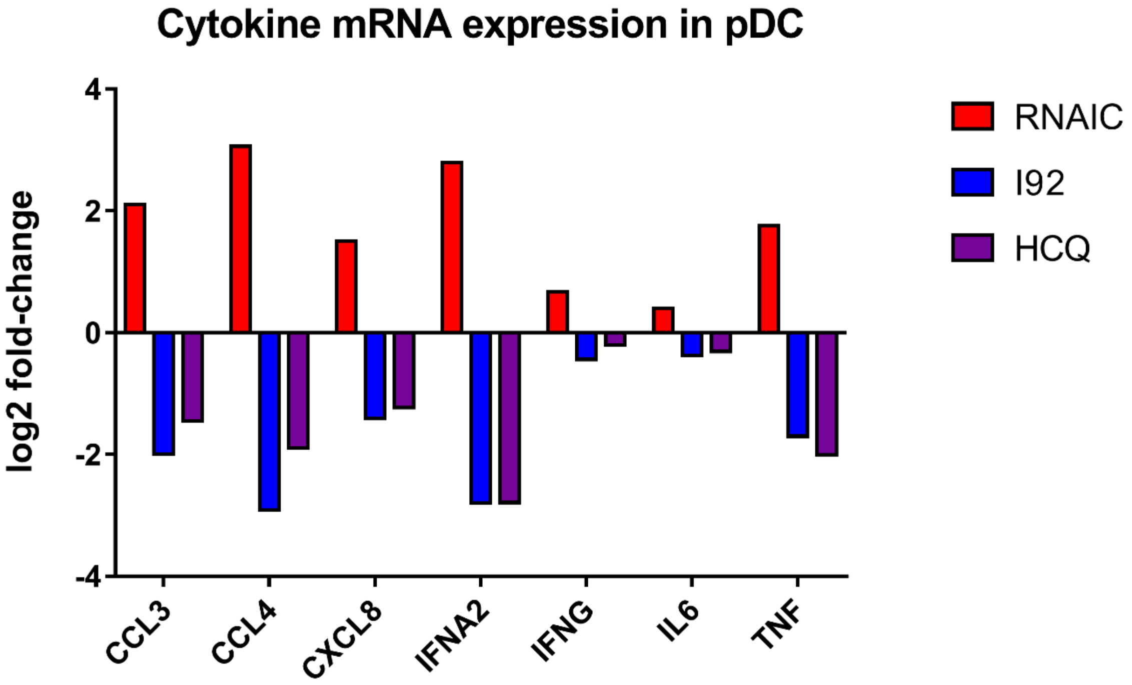

Additional figure S7. Cytokine mRNA expression at 6 h in plasmacytoid dendritic cells (pDCs) stimulated with RNA-containing immune complexes (RNA-IC), analyzed by RNA-Sequencing. From the left CCL3 (MIP1-α), CCL4 (MIP1-β), CXCL8 (IL-8), IFNA2 (IFN-α), IFNG (IFN-γ), IL6 (IL-6), TNF (TNF-α). Red: log2FC in RNA-IC stimulated compared to mock stimulated pDCs. Blue: log2FC in I92 treated vs untreated RNA-IC stimulated cells. Purple: log2FC in HCQ treated vs untreated RNA-IC stimulated cells.

CCL, C-C motif chemokine ligand; MIP, macrophage inflammatory protein; CXCL, C-X-C motif chemokine ligand; FC, fold change
