# Supplementary material for: Cytokine production by activated plasmacytoid dendritic cells and natural killer cells is suppressed by an IRAK4 inhibitor
Source: Arthritis Res Ther. 2018 Oct 24;20:238. doi: 10.1186/s13075-018-1702-0 (PMC6235225; doi:10.1186/s13075-018-1702-0)
Supplement: Supplementary file 13 — Figure S8. TNF-α production in NK cell cultures and NK cell/pDC cocultures. (PDF 179 kb) [file 13075_2018_1702_MOESM13_ESM.pdf]

Additional file 13. TNF-α production in NK cell cultures and NK cell/pDC co-cultures

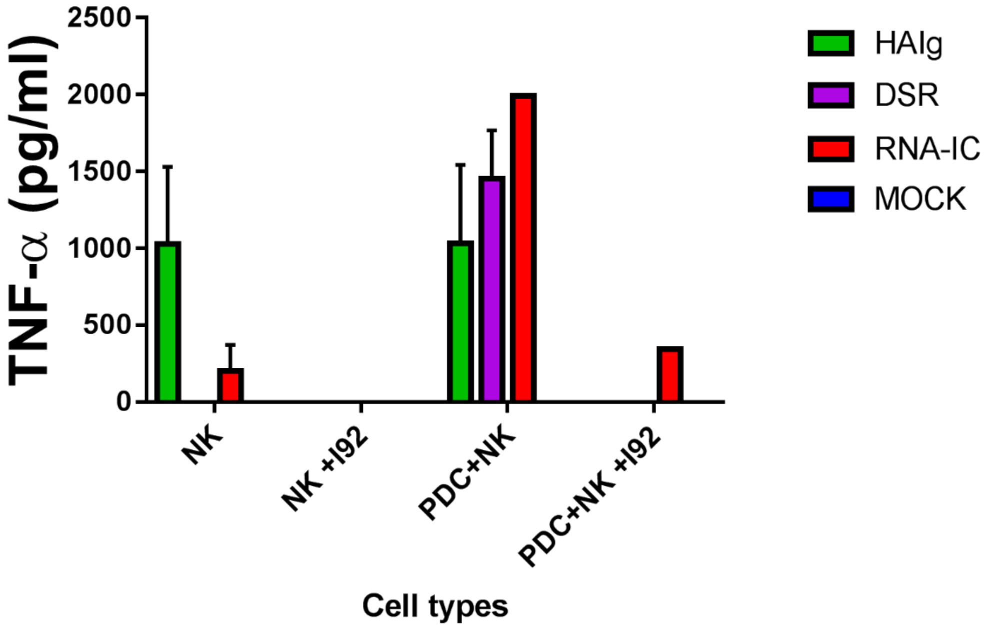

Additional figure S8. Tumor necrosis factor alpha **(**TNF-α) production by NK cells isolated from healthy donors, cultivated separately or in co-cultures with plasmacytoid dendritic cells (pDCs), stimulated with heat-aggregated IgG (HAIg, green), TLR7 agonist DSR6434 (purple), RNA-containing immune complexes (RNA-IC, red) or culture medium only (MOCK, blue), in the presence or absence of an Interleukin-1 Receptor Associated Kinase (IRAK) 4 inhibitor (I92). The levels of TNF-α were measured by an immunoassay after 20 h. Bars represent the mean with SEM of 2 donors.
