# Supplementary material for: Cytokine production by activated plasmacytoid dendritic cells and natural killer cells is suppressed by an IRAK4 inhibitor
Source: Arthritis Res Ther. 2018 Oct 24;20:238. doi: 10.1186/s13075-018-1702-0 (PMC6235225; doi:10.1186/s13075-018-1702-0)
Supplement: Supplementary file 14 — Figure S9. Flow cytometric analysis of TNF-α in NK cells. (PDF 165 kb) [file 13075_2018_1702_MOESM14_ESM.pdf]

Additional file 14. Flow cytometric analysis of TNF-α in NK-cells

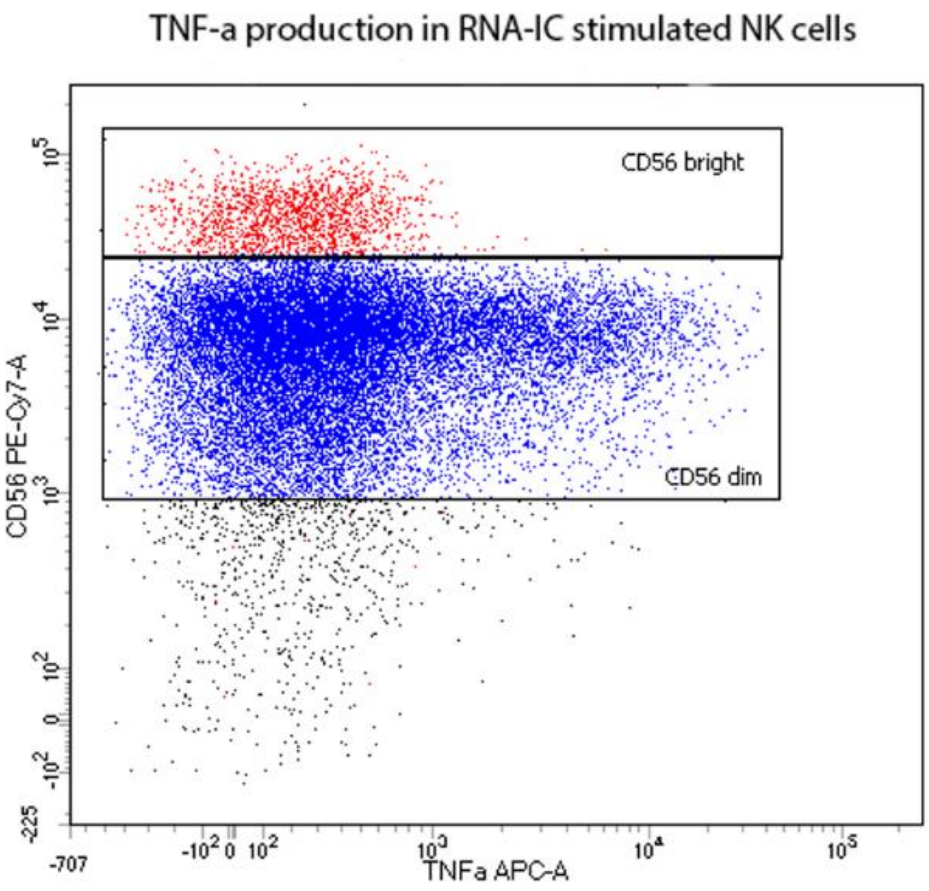

Additional figure S9. Flow cytometry showing intracellular TNF-α production in CD56 dim NK cells, but not in CD56 bright NK cells. NK cells from healthy donors were cultivated in the presence of RNA-containing immune complexes (RNA-IC) for 5 hours. Red: CD56 bright NK cells, blue: CD56 dim NK cells. No cytokines were detected in cell cultures in the absence of RNA-IC.

The dot plot represents one individual donor of four analyzed**.**
