# Supplementary material for: Cytokine production by activated plasmacytoid dendritic cells and natural killer cells is suppressed by an IRAK4 inhibitor
Source: Arthritis Res Ther. 2018 Oct 24;20:238. doi: 10.1186/s13075-018-1702-0 (PMC6235225; doi:10.1186/s13075-018-1702-0)
Supplement: Supplementary file 15 — Figure S10. Interleukin-8 production by stimulated blood cells from SLE patients. (PDF 104 kb) [file 13075_2018_1702_MOESM15_ESM.pdf]

Additional file 15. Interleukin-8 production by stimulated blood cells from SLE-patients

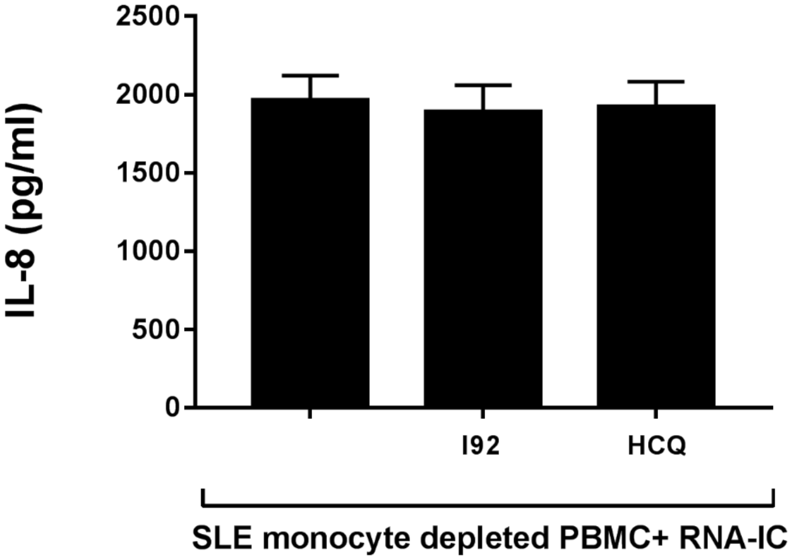

Additional figure S10. IL-8 production by monocyte-depleted peripheral blood mononuclear cells (PBMC) from SLE patients. The cells were stimulated with RNA-containing immune complexes (RNA-IC), in the presence or absence of IRAK4 inhibitor I92 or hydroxychloroquine (HCQ). IL-8 production in cell cultures was measured after 20 h by an immunoassay. IL-8 levels exceeded the highest measurable value of the standard curve in all but two donors. Bars represent the mean with SEM, based on 13 donors, in 10 independent experiments.
